# Accurate *de novo* design of membrane-traversing macrocycles

**DOI:** 10.1016/j.cell.2022.07.019

**Published:** 2022-09-15

**Authors:** Gaurav Bhardwaj, Jacob O’Connor, Stephen Rettie, Yen-Hua Huang, Theresa A. Ramelot, Vikram Khipple Mulligan, Gizem Gokce Alpkilic, Jonathan Palmer, Asim K. Bera, Matthew J. Bick, Maddalena Di Piazza, Xinting Li, Parisa Hosseinzadeh, Timothy W. Craven, Roberto Tejero, Anna Lauko, Ryan Choi, Calina Glynn, Linlin Dong, Robert Griffin, Wesley C. van Voorhis, Jose Rodriguez, Lance Stewart, Gaetano T. Montelione, David Craik, David Baker

**Affiliations:** 1 Institute for Protein Design, University of Washington, Seattle, WA 98195, USA; 2 Department of Medicinal Chemistry, University of Washington, Seattle, WA 98195, USA; 3 Biological Physics, Structure and Design program, University of Washington, Seattle, WA 98195, USA; 4 Department of Biochemistry, University of Washington, Seattle, WA 98195, USA; 5 Molecular Cell and Biology program, University of Washington, Seattle, WA 98195, USA; 6 Institute for Molecular Bioscience, Australian Research Council Centre of Excellence for Innovations in Peptide and Protein Science, The University of Queensland, Brisbane, QLD 4072, Australia; 7 Department of Chemistry and Chemical Biology and Center for Biotechnology and Interdisciplinary Sciences, Rensselaer Polytechnic Institute, Troy, NY 12180, USA; 8 Molecular Engineering and Sciences Program, University of Washington, Seattle, WA 98195, USA; 9 Departamento de Quίmica Fίsica, Universidad de Valencia, Avenida Dr. Moliner 50, Burjassot, 46100 Valencia, Spain; 10 Department of Medicine, Division of Allergy and Infectious Disease, University of Washington, Seattle, WA, USA; 11 Department of Chemistry and Biochemistry, University of California-Los Angeles, Los Angeles, CA, USA; 12 Takeda Pharmaceuticals Inc., Cambridge, MA, USA; 13 Howard Hughes Medical Institute, University of Washington, Seattle, WA 98195, USA

**Keywords:** computational design, peptide design, membrane permeability, oral bioavailability

## Abstract

We use computational design coupled with experimental characterization to systematically investigate the design principles for macrocycle membrane permeability and oral bioavailability. We designed 184 6–12 residue macrocycles with a wide range of predicted structures containing noncanonical backbone modifications and experimentally determined structures of 35; 29 are very close to the computational models. With such control, we show that membrane permeability can be systematically achieved by ensuring all amide (NH) groups are engaged in internal hydrogen bonding interactions. 84 designs over the 6–12 residue size range cross membranes with an apparent permeability greater than 1 × 10^−6^ cm/s. Designs with exposed NH groups can be made membrane permeable through the design of an alternative isoenergetic fully hydrogen-bonded state favored in the lipid membrane. The ability to robustly design membrane-permeable and orally bioavailable peptides with high structural accuracy should contribute to the next generation of designed macrocycle therapeutics.

## Highlights

• Computational design of diverse permeable macrocycles beyond the “rule-of-five” space
• X-ray and NMR structures of designed macrocycles match their computational models
• Designed macrocycles are permeable *in vitro* and orally bioavailable *in vivo*
• Designed chameleonic peptides show solvent-dependent conformational switching

## Graphical abstract

**Figure:**
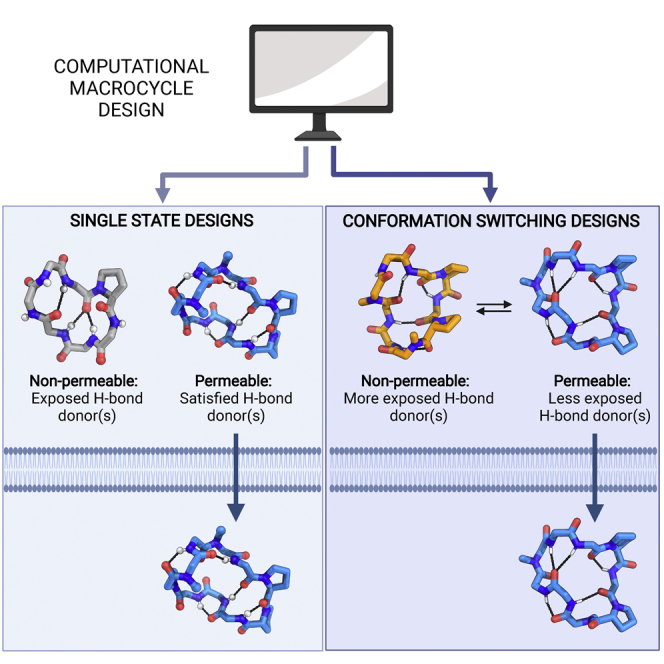

## Teaser

An investigation of the design principles of macrocyclic peptide membrane permeability and oral bioavailability enables the generation of synthetic macrocycles that fold into the predicted conformation, can cross membranes, and even adopt different conformations depending on polar versus nonpolar contexts.

## Introduction

Macrocyclic peptides have considerable potential as therapeutics with advantages over small molecules in the ability to disrupt protein-protein interactions, and over proteins in metabolic stability and ability to cross biological membranes (Muttenthaler et al., 2021). Peptides with intrinsic membrane permeability can access intracellular drug targets, translocate across intestinal epithelial cells enabling oral delivery, and penetrate the blood-brain barrier by traversing brain microvascular endothelial cells. Naturally occurring macrocycles, such as cyclosporine A and griselimycin, suggest potential mechanisms for permeability of large macrocycles that are beyond the traditional “rule of five” (Ro5) (Bockus and Lokey, 2017; Bockus et al., 2015; Hewitt et al., 2015; Nielsen et al., 2017). However, adapting the insights from a few naturally permeable macrocycles to develop new membrane-permeable peptides of diverse shapes and sizes has proven very difficult. Studies of naturally permeable macrocycles have identified some common features, such as low polar surface area and lack of unsatisfied hydrogen bond donors (Bockus et al., 2015; Peraro and Kritzer, 2018; Rand et al., 2012; Rezai et al., 2006). Conformational switching between different structures in aqueous and membrane environments has also been described as a potential approach to achieve passive membrane permeability (Peraro and Kritzer, 2018; Wang et al., 2018; White et al., 2011). The incorporation of these features into library-based methods has enabled the development of several new membrane-permeable cyclic peptides. However, previous work has largely been limited to cases of smaller peptides, usually 5–7 amino acids (Hewitt et al., 2015; Hill et al., 2014; Wang et al., 2014; White et al., 2011). Additionally, extending these principles to the design of novel membrane-permeable peptides with extensive chemical and structural diversity has remained challenging, as it requires an understanding of the relationships between structure, flexibility, and permeability and the ability to accurately control the sequence and structural features of macrocycles simultaneously (Mulligan, 2020).

Here, we take advantage of the ability of computational design to specify macrocycle structure to systematically explore the determinants of passive membrane permeability (Bhardwaj et al., 2016; Hosseinzadeh et al., 2017). We use a design-build-test approach where we design peptides containing different structural features, determine their crystal and solution structures, and evaluate their permeability. We consider three structural features: the satisfaction of all hydrogen bond donors through the formation of intrapeptide hydrogen bonds, the presence of *cis*-peptide bonds, and the ability to switch conformations between aqueous and lipid environments.

## Results

**Figure S1:**
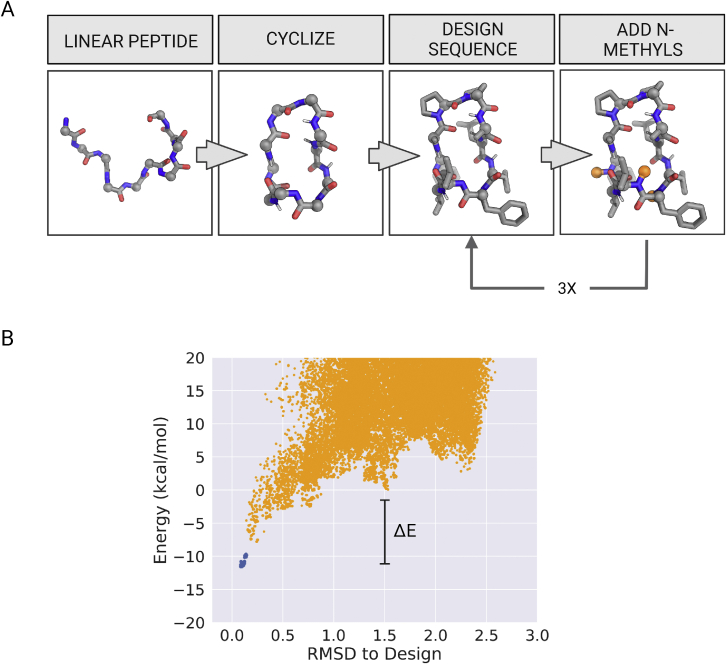
Design and selection of membrane-permeable peptides, related to Figures 1 and 3 and STAR Methods (A) Overall schematic of the *in silico* pipeline for the design of membrane-permeable peptides. Design process starts with a linear polyglycine peptide chain that is cyclized using Rosetta generalized kinematic closure (genKIC) protocol. Iterative rounds of amino acid sequence design and N-methylation of non-hydrogen-bonded NH groups are performed to design low-energy macrocycles with no unsatisfied backbone NH groups. The process is repeated to sample 10^5^–10^6^ design models that are clustered to identify permeable macrocycles with diverse shapes and sizes. (B) An example energy versus RMSD to design plot from structure prediction runs using Rosetta simple_cycpep_predict application. Diverse conformations for a given amino acid sequence are generated using generalized kinematic closure (genKIC) protocol and energy-minimized using Rosetta FastRelax protocol. Each orange point represents an independently predicted structure. Blue dots represent the local minimization of the designed macrocycle structure. Landscapes that funnel into the design structure as the lowest energy structure and have a big energy gap (ΔE) between the designed fold and other unfolded states are selected for experimental characterization.

We first investigated whether designed macrocycles with widely diverse lengths and structures sharing only the property of full internal satisfaction of all NH groups could robustly traverse lipid membranes. We extended the Rosetta generalized kinematic closure (genKIC) method to stochastically generate ensembles of ∼10^6^ N-to-C cyclic backbone conformations for 6–12 residue polyglycine peptides, sampling cyclic backbone conformations by selecting phi/psi torsions randomly from flat-bottom symmetric Ramachandran tables (Bhardwaj et al., 2016; Hosseinzadeh et al., 2017). From these large sets, we selected backbones that make at least two intramolecular hydrogen bonds and carried out Rosetta combinatorial sequence design restricting L- and D-amino acids to negative and positive phi regions of Ramachandran space, respectively, and incorporating conformationally constrained amino acids, such as L-proline, D-proline, and α-aminoisobutyric acid (AIB), at structurally compatible sites. To eliminate exposed and unsatisfied hydrogen bond donors, amino acids with an unsatisfied hydrogen bond donor in the backbone were mutated to their N-methylated variants, and only nonpolar amino acids were allowed during the sequence design step (see STAR Methods and Figure S1, Figure S10A). We selected low-energy designs with two or more intramolecular hydrogen bonds and five or fewer N-methylated amino acids. The conformational energy landscape for selected designs was characterized by generating 10^5^–10^6^ alternative conformations and evaluating the energy and backbone RMSD to the design model. We chose sequences with funnel-like energy landscapes converging on their corresponding design models (see STAR Methods and Figure S1, Figure S10B). Overall structural diversity was assessed using a backbone torsion angle-based clustering method (Hosseinzadeh et al., 2017). Each residue was assigned a torsion bin (A [right-handed helical region], B [right-handed strand region], X [mirror of A], Y [mirror of B], O [amino acids with phi < 0 and *cis*-peptide bond between the residue i and i+1], and Z [amino acids with phi > 0 and *cis*-peptide bond between the residues i and i+1]), and the resultant torsion bin strings (for example, XYABOX for a six residue peptide) were clustered. Because the choice of a starting residue is arbitrary in a cycle, and both passive membrane permeability and fold propensity are invariant to mirroring, clusters with bin strings that transform into each other under circular permutation or mirror inversion were combined, and members of the resulting nondegenerate clusters (which we represent by the lowest alphabetical order of the torsion bin string over all permutations and inversions) were selected for chemical synthesis and experimental characterization.

### Membrane permeability of 6–8 residue designed macrocycles

We first tested the ability of our design pipeline to control both macrocycle structure and membrane permeability on 6–8 residue macrocycles spanning a diversity of structures and N-methylation patterns. For 6, 7, and 8 residue macrocycles, we selected 8, 5, and 19 designs (representing 6, 5, and 16 clusters), respectively, with fully satisfied backbone NH groups and funnel-like energy landscapes and spanning a diversity of sequences, structures, N-methylation patterns, and structural motifs (see Data S1 and S2 for sequences and design models). Selected macrocycles were chemically synthesized and purified using reverse-phase high-performance liquid chromatography (RP-HPLC). In some cases, two peaks were observed with the expected MW; in such cases we experimentally tested the two peaks separately and refer to these as p1 and p2 in the design names.

**Figure 1:**
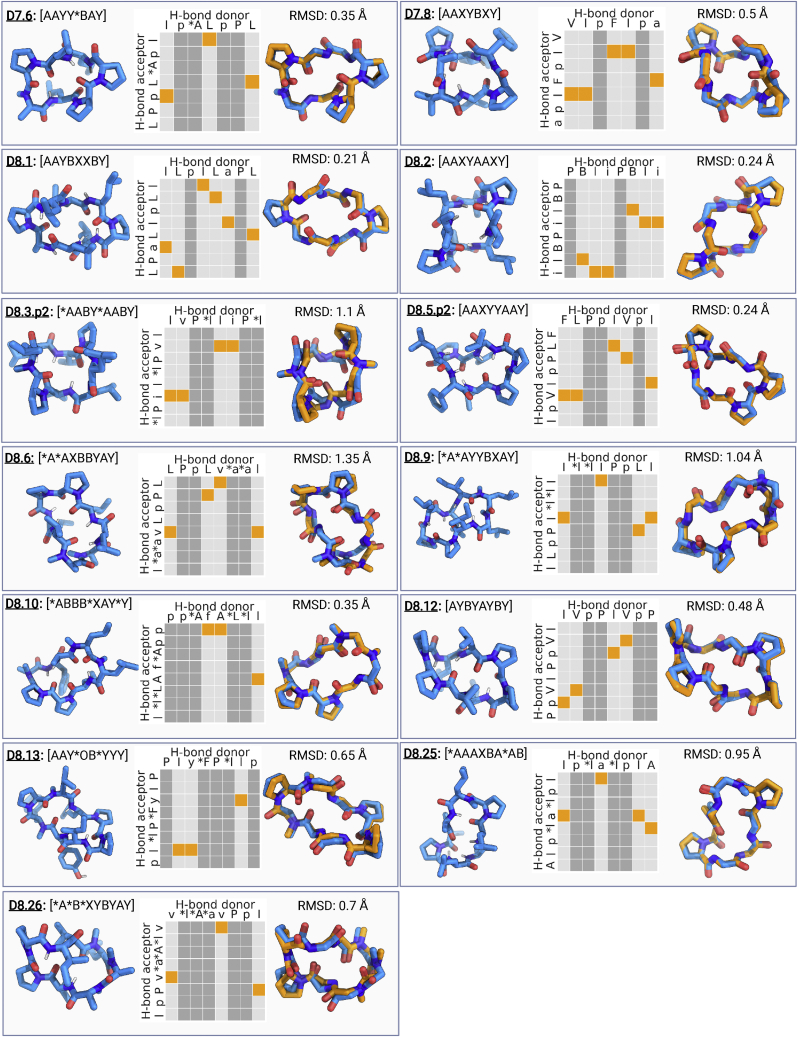
Computational design and structure validation of 6–8 amino acid macrocycles Structural validation of computationally designed macrocycles. Each panel shows the design model and torsion bin string describing the design model (left), hydrogen bonding pattern for the design model (middle), and superposition between the design model (blue) and the X-ray structure (orange) (right). For hydrogen bonding graphs, the orange boxes highlight the designed intramolecular hydrogen bonds. Amino acids without a backbone hydrogen bond donor (proline, D-proline, and N-methylated amino acids) are marked by darker gray columns. Sidechains for non proline residues not shown for clarity in the superposition graphs. RMSD between the design model and X-ray structure was calculated over all backbone heavy atoms (C, CA, N, O, and CN). ^∗^ in the macrocycle sequence denotes the N-methylated amino acid positions and lower case denotes the D-amino acids. See also Table S1, Figure S1, Figure S2, Figure S3, Figure S4, Figure S10, and Data S1 and S2.

To evaluate the accuracy of the design method, we determined X-ray crystal structures for two 7 residue designs and fifteen 8 residue designs. The structures for two 7 residue and twelve of the 8 residue macrocycles were very close to the computational design models (backbone atom RMSD < 1.2 Å) (Figure 1; Table S1). In seven cases with remarkably low RMSDs below 0.5 Å, the design models are within the experimental resolution of the X-ray data. Three of the designs, D8.1, D8.2, and D8.12, are internally symmetric: D8.1 with a backbone RMSD to the design model of 0.21 Å has an internal S2 mirror symmetry composed of four beta turns and two gamma turn motifs, D8.2 has C2 symmetry with a combination of 4 beta turns and 2 alpha turns (RMSD 0.24 Å), and D8.12 has C2 symmetry with four internal hydrogen bonds and no N-methylated amino acids (RMSD 0.48 Å) (Figure 1). Design D8.5.p2 with no N-methylated amino acids is stabilized by four internal hydrogen bonds forming four beta turns and an alpha turn motif (RMSD 0.24 Å). D8.10 with 0.35 Å RMSD between model and X-ray structure has three N-methylated amino acids and two proline residues in its sequence; its other three amino acids are involved in three internal hydrogen bonds. D7.6 features three beta turns and single N-methylated amino acid and matches very closely with the design (RMSD 0.35 Å). D7.8 has five internal hydrogen bonds and no N-methylated amino acids (RMSD 0.5 Å). Overall, the close matches between the experimental structures and the design models validate that our approach can very accurately specify macrocycle structure (Table S1).

**Figure 2:**
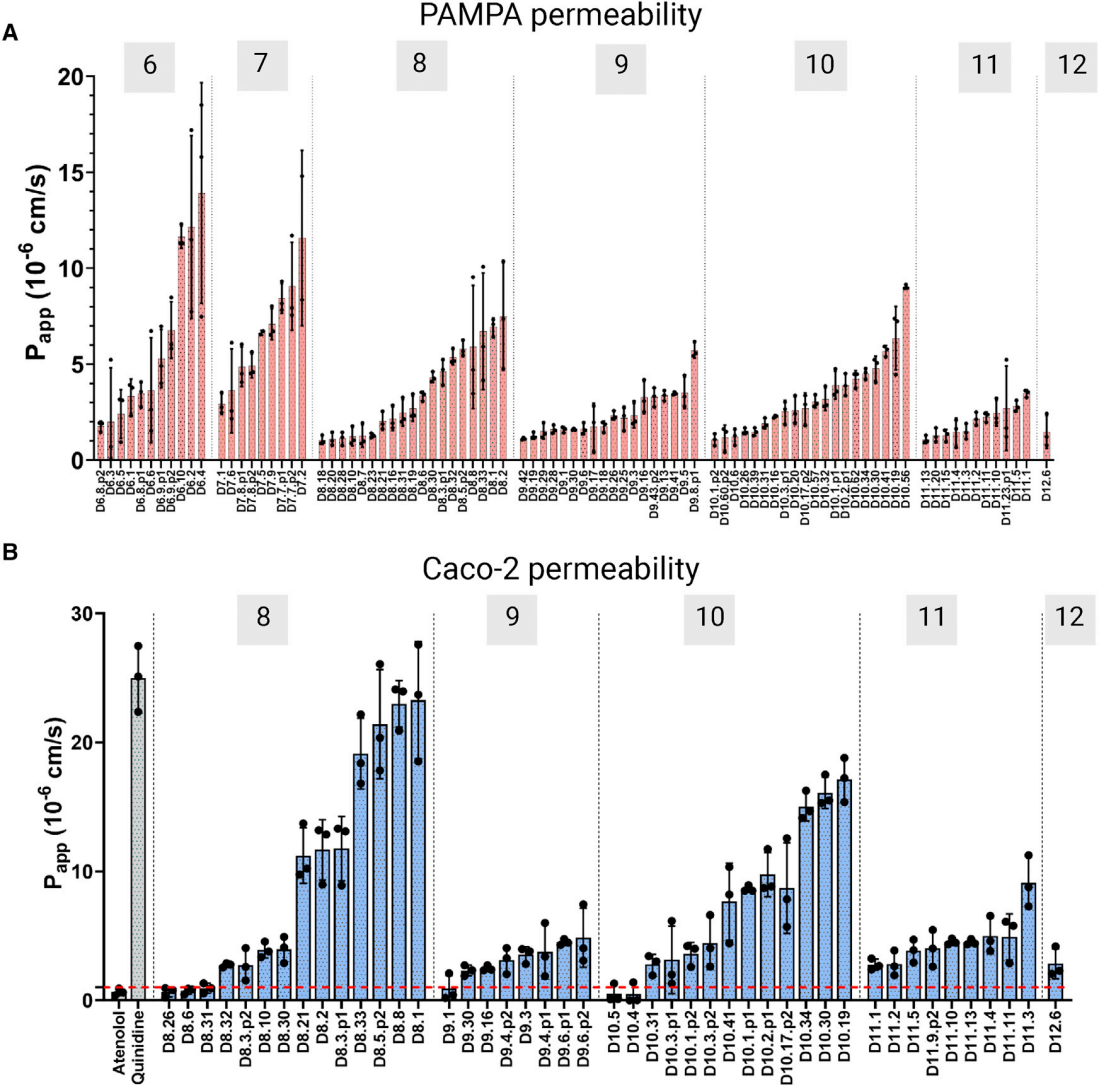
Permeability measurements of computationally designed macrocycles in PAMPA and Caco-2 assays (A) Apparent permeability (P_app_) of 6–12 amino acid macrocycles in PAMPA assay. Peptides are grouped based on sequence length. Isobaric peaks (denoted p1 and p2) were seen for some peptides during purification and were assayed separately. Bar height: average P_app_ from three replicates; error bars: standard deviation calculated from three replicates. (B) Apparent permeability (P_app_) of designed 8–12 amino acid macrocycles (salmon-colored bars) measured in the apical to the basal direction in the Caco-2 assays. P_app_ for quinidine and atenolol used as negative and positive controls (gray-colored bars). Bar height: average P_app_ from three replicates; error bars: standard deviation calculated from three replicates. See also Data S3.

Having found that the macrocycles fold as designed, we next investigated their membrane permeabilities using transwell permeability assays. The rates of traversal across artificial membranes in parallel artificial membrane permeability assays (PAMPAs) (Di et al., 2003) were determined by mass-spectrometry-based quantification of peptide concentrations in the donor and acceptor wells (see STAR Methods). Eight 6-mers, five 7-mers, and sixteen 8-mers had apparent permeabilities (P_app_) greater than 1 × 10^−7^ cm/s (see Data S3). Of these, eight 6-mers, five 7-mers, and ten 8-mers had P_app_ greater than 1 × 10^−6^ cm/s (Figure 2A). PAMPA is a good measure of passive transport across lipid membranes; however, oral bioavailability *in vivo* involves cellular barriers with efflux transporters. Therefore, we tested a subset of the designed macrocycles in transwell Caco-2 assays, with colorectal epithelial cells as the barrier between the donor and acceptor wells, and in the opposite direction of the efflux transport. In Caco-2 assays, permeability greater than 1 × 10^−6^ cm/s is considered indicative of adequate cellular permeability for a candidate drug. Because Caco-2 assays are resource intensive, we focused on testing the larger-sized 8 residue macrocycles and did not carry out this assay on the 6 and 7 residue macrocycles. We again observed high permeability (Figure 2B) in Caco-2 assays: out of the eight 8-mer designs tested in Caco-2 assays, 6 designs had P_app_ greater than 1 × 10^−6^ cm/s, and 4 designs higher than 1 × 10^−5^ cm/s. Design D8.1 with no N-methylated amino acids showed a very high P_app_ of 23.27 × 10^−6^ cm/s.

**Figure S2:**
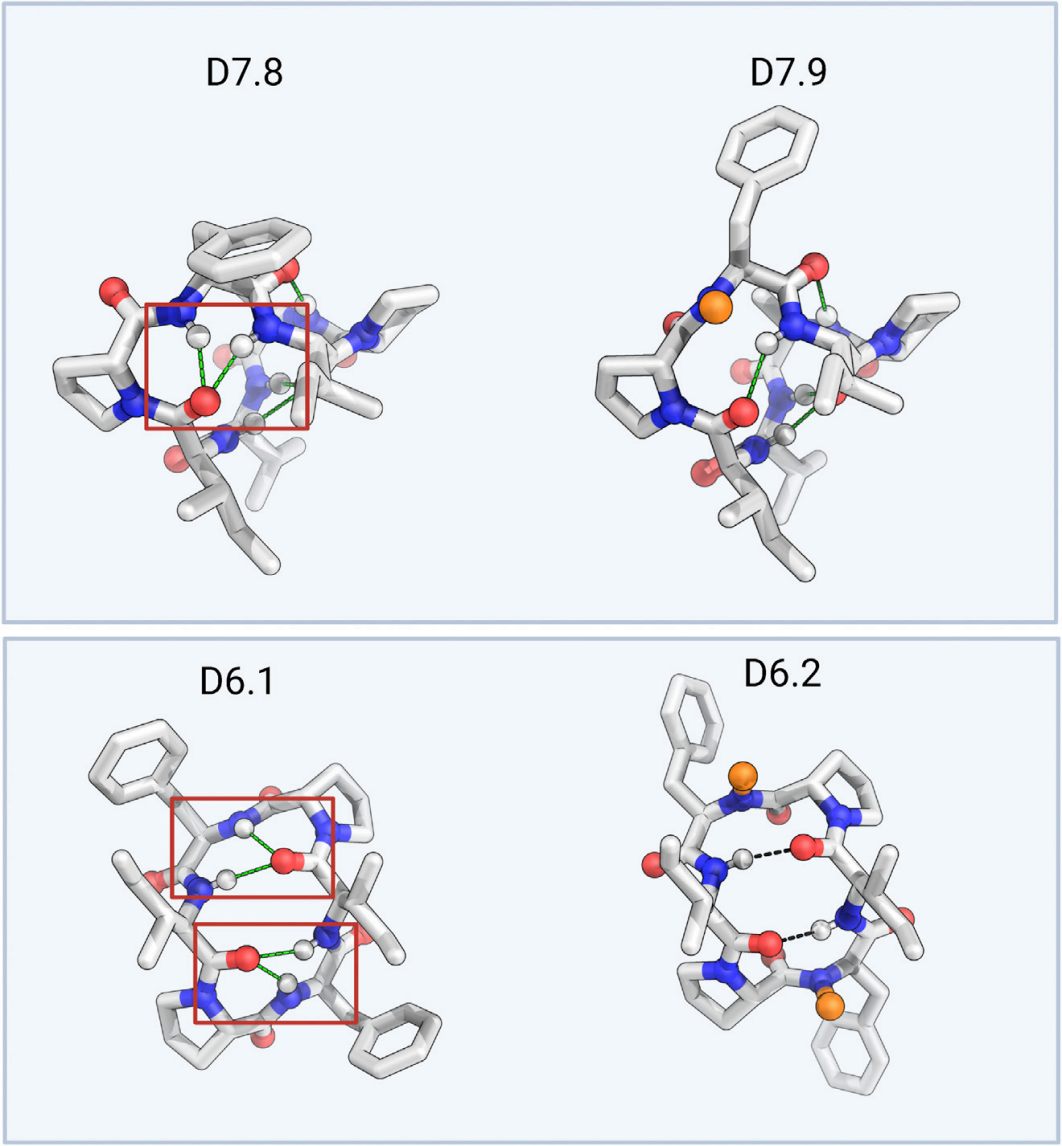
N-methylation of geometrically strained turn types, related to Figure 1 Examples of geometrically strained arrangement of overlapping gamma and beta turns seen in some of the design models. For such designs, variants with N-methylated middle residue were also generated and tested experimentally. N-methyls are shown as orange. Intramolecular hydrogen bonding interactions are shown as green dashes.

The membrane-traversing macrocycles with P_app_ greater than 1 × 10^−6^ cm/s and funnel-like energy landscapes cover a wide range of structures, populating six, five, and nine clusters for 6, 7, and 8 residue macrocycles, respectively. While previous studies with natural scaffolds and library-based methods have identified permeable macrocycles, these usually require multiple N-methylated or N-alkylated amino acids. For example, 7 of 11 amino acids in cyclosporine are N-methylated, which comes at the cost of increased molecular flexibility and synthetic difficulties. In our 6–8 residue permeable designs, the number of N-methylated amino acids ranges between 0 and 3. Five of the structurally validated designs, D7.8, D8.1, D8.2, D8.5, and D8.12, have no N-methyl amino acids highlighting the precise control offered by computational methods to design structures with complete hydrogen bond donor satisfaction through internal hydrogen bonding and prolines (Figure 1; Data S2). D8.1, D8.2, D8.5.p2, and D8.12 are the largest passively permeable macrocycles we are aware of that lack N-methylated or N-alkylated amino acids. The extensive internal hydrogen bonding enables full NH bond satisfaction in these designs with no N-methylated amino acids; designs D8.1 and D8.2 have six internal hydrogen bonds that stabilize the structure. Some of the 6 and 7 residue design models (D6.3, D6.5, D6.9, and D7.8) contain a geometrically strained arrangement of overlapping beta and gamma turns (Figure S2), in which the middle amino acid may have a partially or fully unsatisfied NH group. Accordingly, we also designed and tested variants with the middle amino acid N-methylated, and in all cases, the variant with the additional N-methylation is more permeable than the original. However, across all the permeable designs, permeability does not correlate with the number of N-methylated amino acids; for example, D8.1 is the most permeable 8 residue design in PAMPA and Caco-2 assays and does not have any N-methylated amino acids. This illustrates that maximizing hydrogen bond satisfaction and using N-methylation sparingly in the folded structure is a viable strategy for achieving permeability.

### Design of membrane-permeable 9–12 residue macrocycles

Earlier work on passively permeable peptides has been primarily limited to 5–7 amino acids with a few cases of larger peptide derivatives of natural products (Hewitt et al., 2015; Hill et al., 2014; Wang et al., 2014; White et al., 2011). This is because purely lipophilic peptides show a steep decrease in permeability with size, as observed in a study of 8-mer, 9-mer, and 10-mer peptides with all their residues N-methylated (Pye et al., 2017). To determine whether our design principles can circumvent this trend, we used our computational pipeline to design a wide variety of larger macrocycles ranging from 9 to 12 amino acids.

We selected for synthesis and characterization seventeen 9-mer, forty-one 10-mer, nineteen 11-mer, and eight 12-mer macrocycles with funnel-like energy landscapes spanning 16, 37, 18, and 8 different structural clusters, respectively, with widely ranging structures and between 1 and 6 N-methylated amino acids (see Data files S1 and S2 for sequences and design models). 50 designs spanning 12, 23, 13, and 2 structural clusters for 9, 10, 11, and 12 residue macrocycles, respectively, were permeable in PAMPA assays with apparent permeability greater than 1 × 10^−7^ cm/s (see Data S3). Out of these, ten 9-mers, sixteen 10-mers, nine 11-mers, and one 12-mer showed significant permeability (P_app_ > 1 × 10^−6^ cm/s) in PAMPA assays (Figure 2A). There was a size dependence in their permeability, but the drop-off is less steep than what has been observed in previous studies on non-designed macrocycles, resulting in significant permeabilities beyond the typical size range for permeable drug like compounds ("rule of five”, see introduction). In Caco-2 assays, three 9-mer, eight 10-mer, seven 11-mer designs, and one 12-mer had P_app_ greater than 1 × 10^−6^ cm/s (Figure 2B). Despite their large size, multiple 10 and 11 residue designs show considerably high P_app_ in Caco-2 assays: D10.19, D10.30, and D10.34 have rates greater than 1 × 10^−5^ cm/s, and three other designs have P_app_ between 0.5 and 1.0 × 10^−5^ cm/s. One 11-mer design, D11.3, has a high P_app_ of 9.11 × 10^−6^ cm/s in Caco-2 assays (Figure 2B; Data S3). Most of the 12 residue designs tested were not permeable, but D12.6 has a P_app_ value of 1.47 × 10^−6^ cm/s and 2.84 × 10^−6^ cm/s in the PAMPA and Caco-2 assays, respectively (Figure 2B).

**Figure 3:**
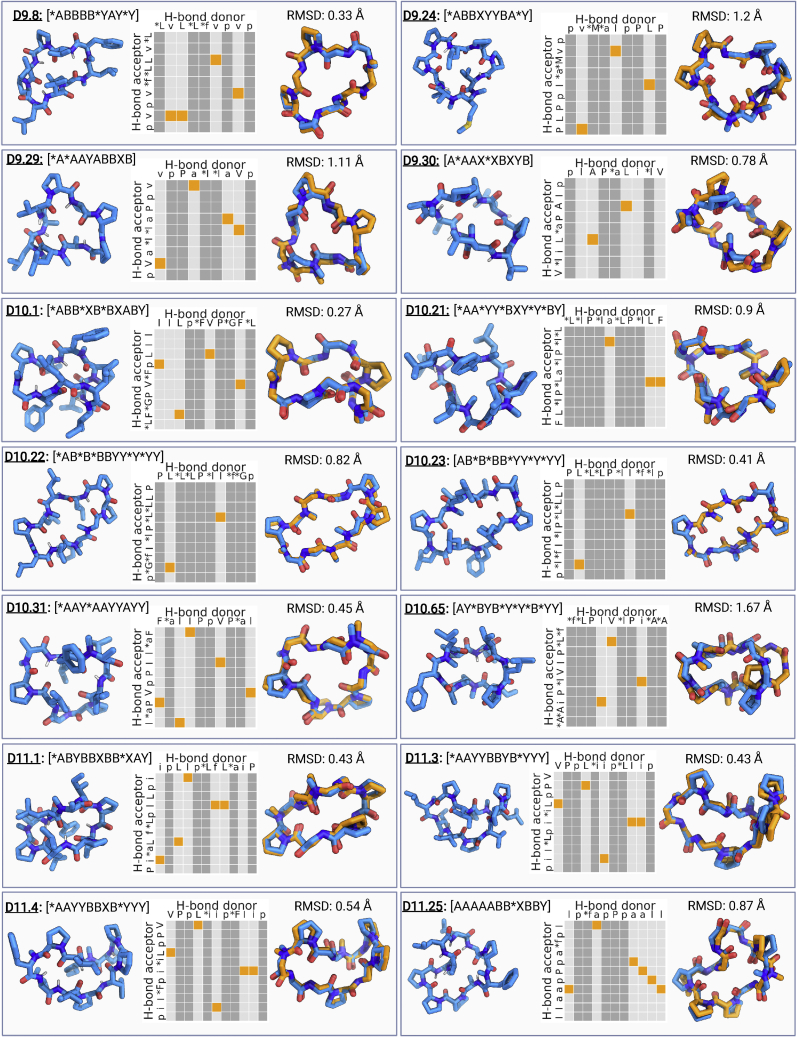
Computational design and structure validation of 9–12 amino acid macrocycles Structural validation of computationally designed macrocycles. Each panel shows the design model and torsion bin string describing the design model (left), hydrogen bonding pattern for the design model (middle), and superposition between the design model (blue) and the X-ray structure (orange) (right). For hydrogen bonding graphs, the orange boxes highlight the designed intramolecular hydrogen bonds. Amino acids without a backbone hydrogen bond donor (proline, D-proline, and N-methylated amino acids) are marked by darker gray columns. Side chains for non proline residues not shown for clarity in the superposition graphs. RMSD between the design model and X-ray structure was calculated over all the backbone heavy atoms (C, CA, N, O, and CN). ^∗^ in macrocycle sequence denotes the N-methylated amino acid positions and lowercase denotes the D-amino acids. See also Table S2, Figures S1, S3, and S5, and Data S1 and S2.

To evaluate the structural accuracy of our design models and to confirm structure-activity relationships present in the design models and membrane permeability data described above, we sought to determine their experimental structures. We successfully crystallized and solved the high-resolution X-ray crystal structures for five 9-mers, six 10-mers, and four 11-mers. Out of these 15 structures, three 9-mers, five 10-mers, and four 11-mer macrocycles matched closely (backbone RMSD of 1.2 Å or less) with their design models (Figure 3; Table S2). Design D9.8 (RMSD 0.33 Å) features three N-methylated amino acids, and the structure is stabilized by 1 alpha turn, 2 beta turns, and 1 gamma turn. D10.31 (RMSD 0.45 Å) has two N-methylated amino acids and five internal hydrogen bonds stabilizing the macrocyclic structure; structures solved in isopropanol:water and ethyl acetate:pentane mixtures were identical. Design D10.1 contains five intramolecular hydrogen bonds, and the crystal structure is nearly identical to the design model (backbone RMSD of 0.27 Å and all heavy atom RMSD of 0.47 Å). Designs D10.21, D10.22, and D10.23 each contain five N-methylated amino acids; D10.21 is stabilized by three internal hydrogen bonds and two prolines, while D10.22 and D10.23 have two internal hydrogen bonds (backbone RMSDs of 0.9, 0.82, and 0.41 Å, respectively). D11.3 and D11.4 have five internal hydrogen bonds, and their crystal structures have backbone RMSD less than 0.55 Å to the design model. Design D11.1, with a backbone RMSD of 0.43 Å between the design model and the X-ray crystal structure, contains five internal hydrogen bonds, 2 N-methylated amino acids, and three prolines.

**Figure S3:**
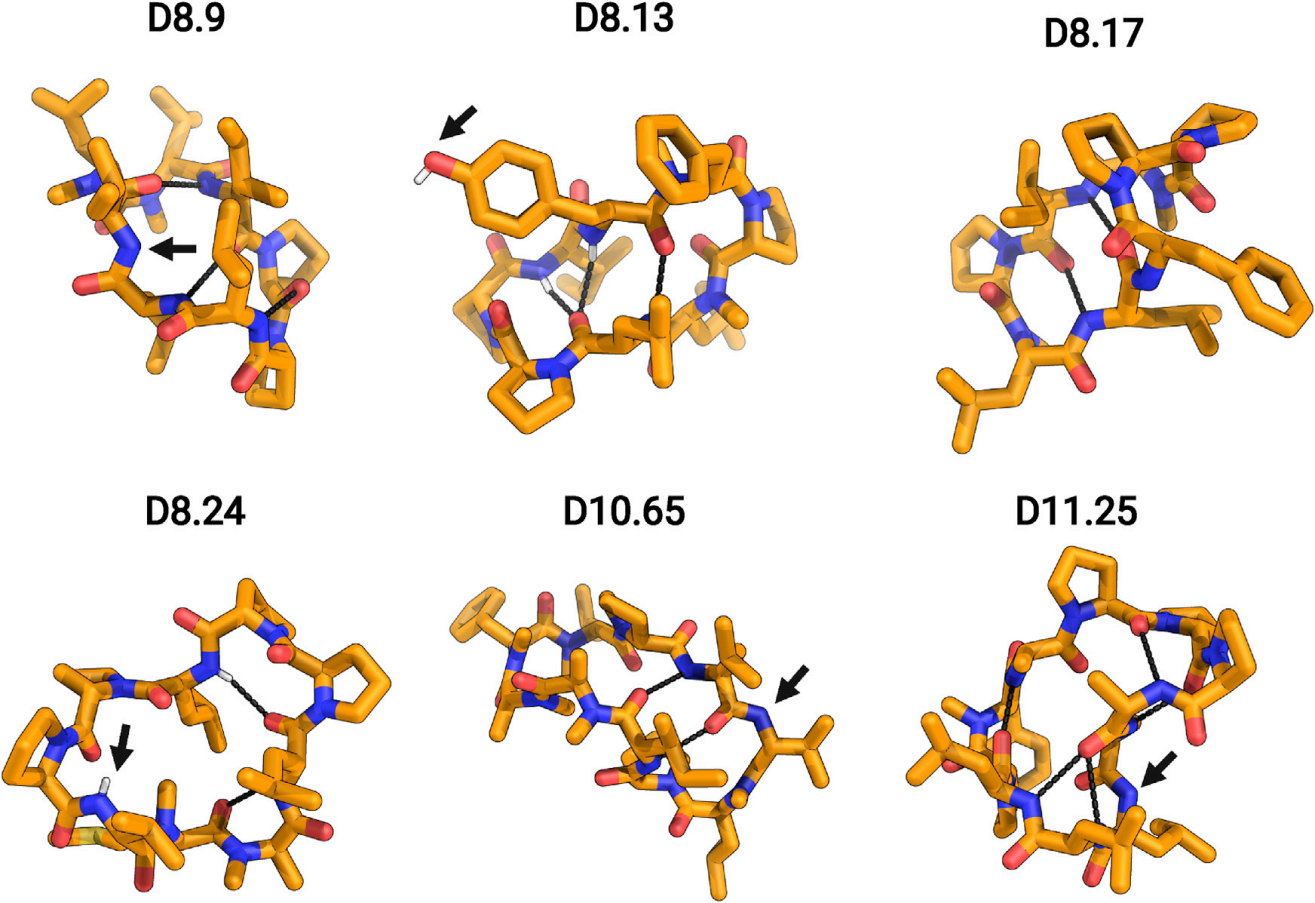
Effect of exposed and unsatisfied polar groups on macrocycle permeability, related to Figures 1 and 3 Designs with exposed polar NH or OH groups in the X-ray crystal structures (orange sticks) are not permeable or show low permeability in PAMPA. Dashed black lines denote the intramolecular hydrogen bonds. The exposed NH or OH groups are denoted by the arrows. See Data S2 and S3 for design models, structures, and permeability data.

The structural accuracy of our designs coupled with a large number of permeability measurements provides insight into the relationship between permeability and NH satisfaction in these larger peptides. Overall, almost all peptides without exposed NH groups were membrane permeable. In contrast to results with nondesigned macrocycles, there was not a strong correlation between permeability and size; indeed some of the 10 and 11 residue peptides were highly permeable. Crystal structures of macrocycles that were not permeable or show low permeability (Figure S3) further support the importance of NH satisfaction: these did not match the fully satisfied design models and included exposed polar groups. Although hydrogen bond satisfaction appears necessary for permeability, it is not sufficient: X-ray structures for D10.21, D10.22, and D10.23 matched very closely (RMSD < 1 Å) with their design models and had no unsatisfied NH groups but were not permeable in transwell assays.

### Permeability of *cis*-peptide bond-containing macrocycles

Our results thus far establish that computational design for satisfying all NHs can robustly generate highly membrane-permeable designs well beyond the Ro5 limits. We next investigated other possible contributions to membrane permeability. It has been proposed that *cis*-peptide bonds can enhance permeability (Marelli et al., 2015). *cis*-peptide bonds are present in a number of our designs. X-ray crystal structure of design D8.31 has *cis*-peptide bonds at the two N-methylated D-leucines at residue positions 3 and 8 that are part of a rare beta-turn formed by a Nme-D-Pro(i+1)–Nme-D-aa(i+2) motif. Design D8.13 contains three prolines with one designed to be in a *cis*-peptide bond stabilized by an aromatic AA(i+1)–Proline(i+2) motif recapitulated in the crystal structure (Ganguly et al., 2012). D8.6 contains two N-methylated amino acids in a row and four intramolecular hydrogen bonds; one of the N-methylated D-alanine undergoes a *trans*-to-*cis* switch in the X-ray crystal structure, but because the switch happens around an N-methylated amino acid, the overall NH satisfaction in the macrocycle is still maintained. Similarly, in design D8.9, an N-methylated D-leucine at amino acid position 3 undergoes a *trans*-to-*cis* switch but maintains the overall satisfaction of the peptide backbone, and the crystal structure of D9.16 also contains a *trans*-to-*cis* omega flip relative to the design model.

**Figure S4:**
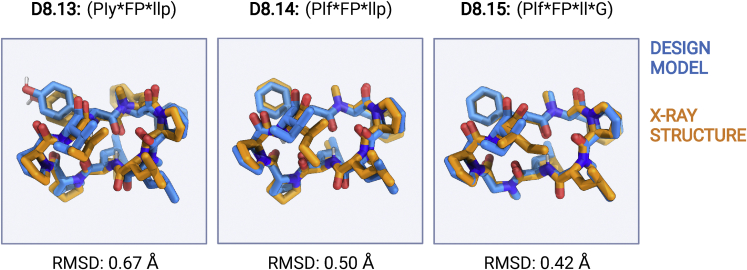
Differences in permeability of macrocycles with *cis*-peptide bonds, related to Figure 1 Superposition between the designed structure (blue sticks) and X-ray structure (orange sticks) of three closely related *cis*-peptide bond-containing designs, D8.13 (left panel), D8.14 (middle panel), and D8.15 (right panel). All three design models match closely (RMSD over all backbone atoms [N, CA, C, O, and CN] < 1.0 Å) with respective X-ray structures. All three design models feature a *cis*-peptide bond in the validated structures. However, the D8.13 is not permeable in PAMPA, while both D8.14 and D8.15 show significant permeability (P_app_ > 1 × 10^−7^ cm/s), indicating that *cis*-peptide bond alone is not enough to drive permeability in these macrocycles.

Over our macrocycle set, there is little association between the presence of *cis*-peptide bonds and the extent of membrane permeability. The permeabilities of *cis*-peptide containing D8.9, D8.13, D8.14, D8.15, D8.6, D9.13, and D10.62 are in the same range as those of all-*trans* macrocycles with the same number of residues. The secondary contribution of *cis*-peptide bonds is further illustrated by D8.13, D8.14, and D8.15, which have similar sequences and structures (Figure S4) with *cis*-peptide bonds at the same position; D8.13 is not membrane permeable, and D8.14 and D8.15 have PAMPA P_app_ of 9.71 × 10^−7^ and 7.68 × 10^−7^ cm/s. D8.13 has a tyrosine residue in place of a phenylalanine residue, creating an unsatisfied OH group that likely prevents permeability.

### Design of membrane-permeable chameleonic macrocycles

**Figure S5:**
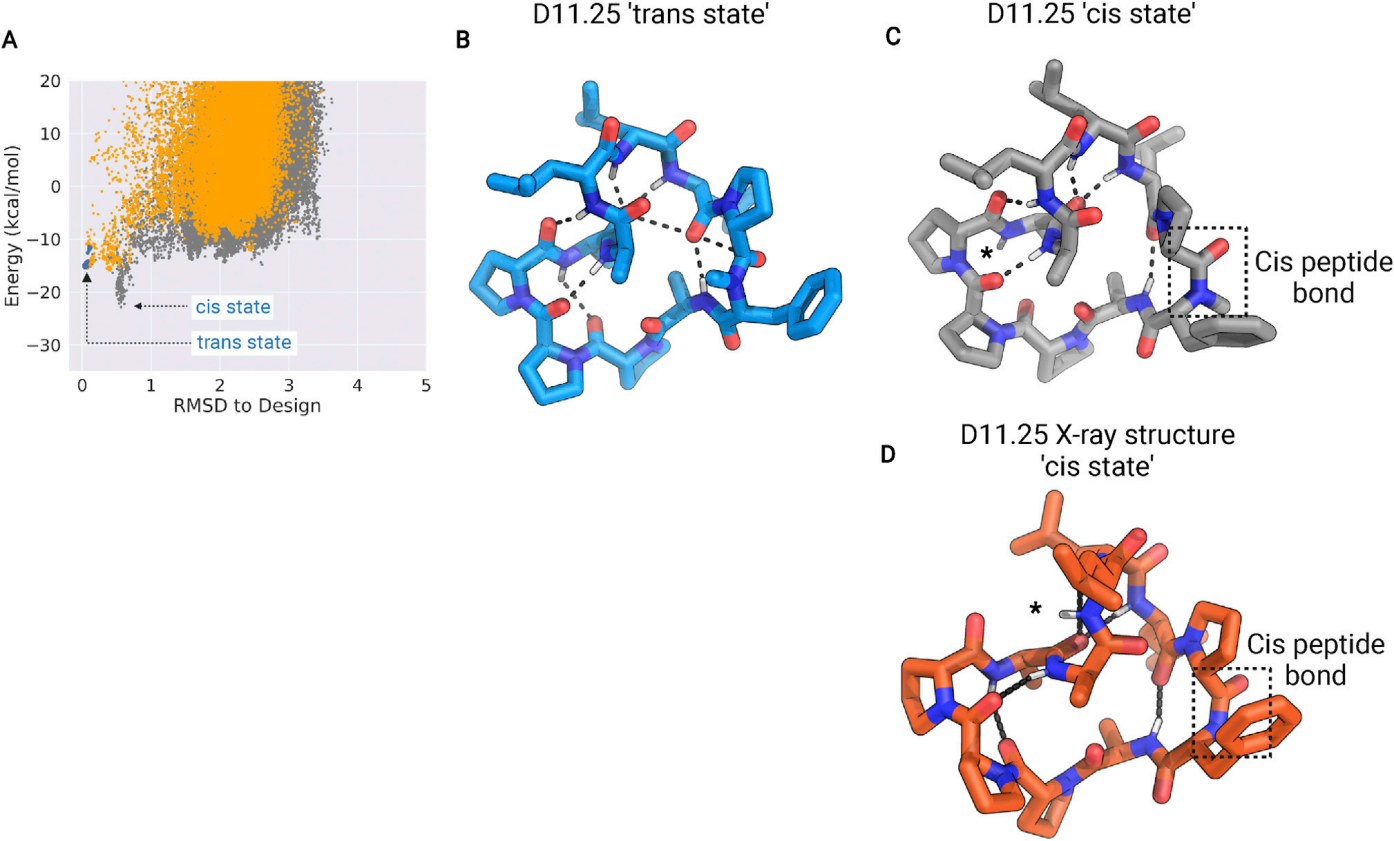
*ccis*-*trans* isomerization of the peptide bonds generates alternative low-energy states, related to Figure 3 (A) Structure prediction calculations for the design D11.25 sequence show two low-energy states. Orange points: conformations with no *cis*-peptide bonds; gray points: predicted conformations with at least one *cis*-peptide bonds; and blue points: conformations generated by the local energy minimization of the design model. (B and C) (B) Lowest energy “*trans*” state with all peptide bonds in *trans* conformation, (C) lowest energy “*cis*” state with the N-methylated amino acid in the *cis* conformation. (D) X-ray structure for D11.25 matches the *cis* state and exposed NH group from a D-leucine. The position of *cis*-peptide bond is highlighted in the dashed square. ^∗^ in the *cis* state denotes the position of the unsatisfied NH groups.

While the above results suggest that *cis*-peptide bonds do not inherently increase membrane permeability, we reasoned that *cis*-*trans* isomerization of the peptide bond could be a powerful design principle for generating peptides with both an open state, with polar groups exposed to interact with a therapeutic target, and a membrane-permeable closed state, with all NH groups making intrapeptide hydrogen bonds (White et al., 2011). The relatively slow (milliseconds timescale) kinetics of *cis*-*trans* isomerization allows for a peptide to populate multiple conformations that can interconvert fast enough to be biologically relevant but are distinguishable by NMR (Grathwohl and Wüthrich, 1981). There is some evidence that isomerization around the *cis*-peptide bond in cyclosporine interconverts distinct binding-competent and membrane-permeable states (Wang et al., 2018). To test this hypothesis, we designed macrocycles that undergo *cis*-*trans* isomerization. D11.25 provides a start in this direction: the structure prediction calculations identified two very similar conformations that differ by a *trans*-to-*cis*-peptide bond flip around the only N-methylated amino acid in the sequence (Figure S5). The all-*trans* conformation is stabilized by six intramolecular hydrogen bonds that satisfy all the available NH groups, while the *cis* form, which is closely recapitulated in the crystal structure (backbone RMSD 0.53 Å), exposes one NH group from D-leucine at position 10 (Figure 3; Figure S13).

We set out to systematically design chameleonic peptides with two distinct energy minima differing by isomerization around a peptide bond: one that has no exposed amides and can traverse membranes, and one that exposes amides to solution and hence has the potential to bind polar target sites. We used three approaches to identify peptides that can populate multiple distinct but almost isoenergetic states (with differences in Rosetta calculated energies of less than 5 kcal/mol). First, we used large-scale structure prediction calculations to generate energy landscapes for many designs (Data file S1), searched for those with two or more minima, and identified 45 with Rosetta calculated energy differences between the two states of less than 5 kcal/mol. Second, for 20 additional cases, where the calculated energy difference between the states was >5 kcal/mol, we developed a genetic algorithm-based multistate design method (see STAR Methods) to optimize the sequence such that the two alternative states have similar energies. Full energy landscape calculations were then performed for these new sequences to confirm the presence of two isoenergetic minima. Third, starting from crystallographically confirmed designs, we introduced destabilizing mutations that led to a second low-energy minimum in the energy landscape, and generated an additional two peptides predicted to adopt two states.

**Figure 4:**
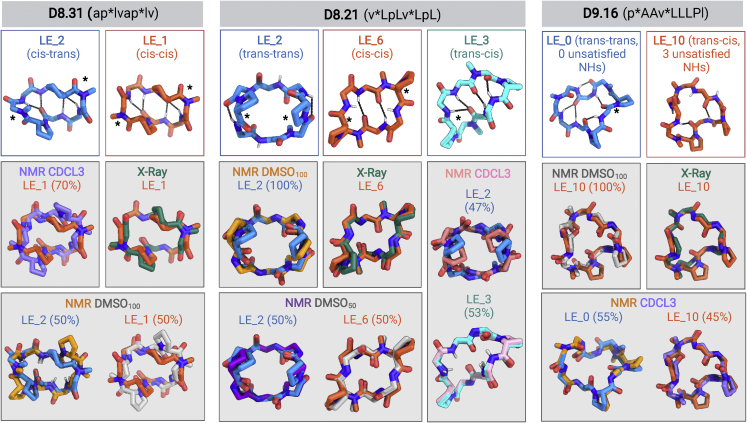
Design and structural characterization of conformation switching macrocycles Design models and experimentally determined structures (X-ray and NMR) for different conformational states of designs D8.31 (left), D8.21 (middle), and D9.16 (right). The design model and predicted low-energy states are shown in the top row. The superposition between the predicted low-energy states and the experimental structures is shown in the gray boxes. The solvent conditions for the NMR structures and low-energy states in superposition plots are indicated by similar colors in the labels and structures. In conditions with multiple conformations, the relative percentage of each conformation in the solution is also indicated. All three designs show solvent-dependent changes in the populations and switching between at least two different conformations. The conformational switch in D8.31 does not change the number of unsatisfied NHs, but for both D8.21 and D9.16, the states with fewer unsatisfied NHs are favored in the nonpolar solvents. See also Tables S3 and S4, and Figure S6, Figure S7, Figure S8, Figure S9, Figure S10.

**Figure S6:**
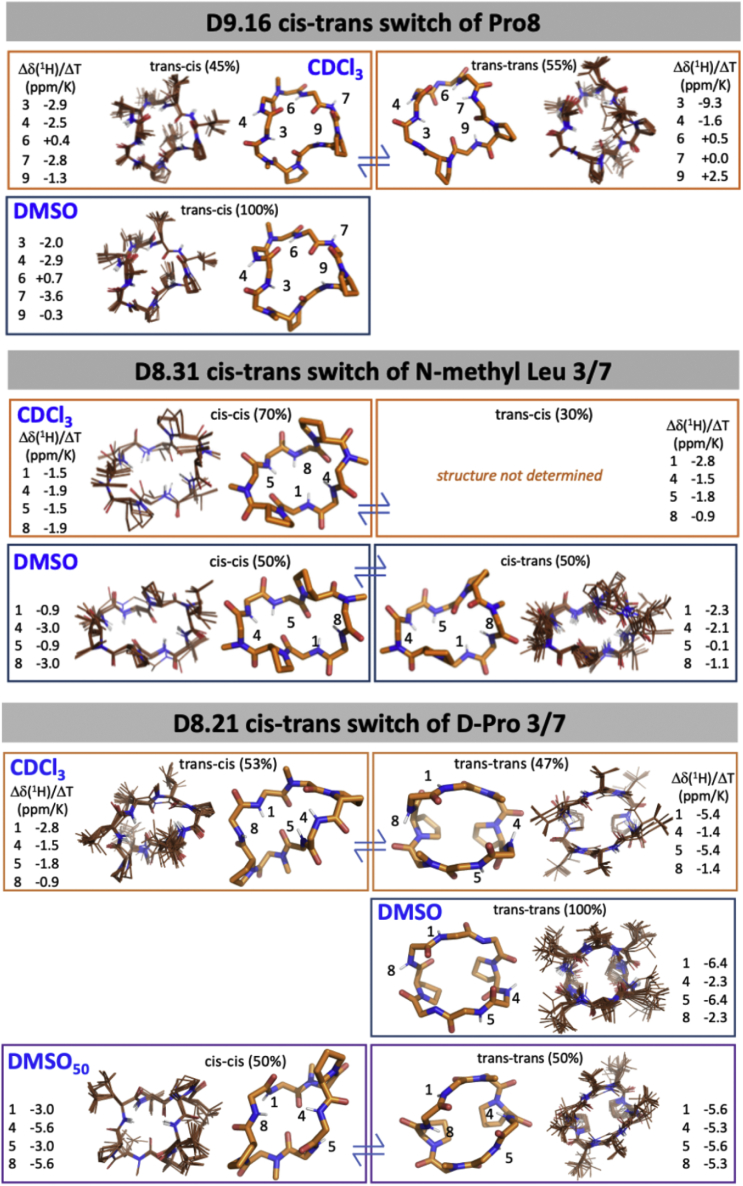
NMR structures in different solvents, related to Figure 4 NMR-derived structures in the indicated solvents d_6_-DMSO, CDCl_3_, or 50:50 d_6_-DMSO/H_2_O (DMSO_50_) are shown for D9.16, D8.31, and D8.21. The overlay structure of the ensemble of 20 lowest energy structures is shown along with the backbone structure of the medoid conformation with NH protons labeled. The amide proton temperature coefficients Δδ(^1^H)/ΔT are given for each of the HN resonances in each conformation. Less negative coefficients indicate increased hydrogen bond propensity and correlate with hydrogen bonds in the structures. In general, upon increasing temperature, amide ^1^H chemical shifts move upfield, which is attributed to a lengthening of the hydrogen bond and decreased shielding from the hydrogen bond acceptor (Baxter and Williamson, 1997). Large changes in chemical shift give large negative temperature coefficients and indicate solvent exposed or weakly hydrogen-bonded NHs. It was empirically found that for proteins in aqueous solution that amide protons with Δδ(^1^H)/ΔT that are more positive than −4.6 ppm/K (less negative and even sometimes positive) are indicative of intramolecular hydrogen bonds (Cierpicki and Otlewski, 2001). For cyclic peptides, temperature coefficients have been used as a measure of hydrogen bonding potential and correlated with MD simulations and predicted structures in aqueous solution as well as in chloroform and DMSO (Wang et al., 2015). In most cases, the NOESY or ROESY data with the ^3^J_hnha_ coupling-derived dihedral restraints was sufficient to give a converged structure for the peptides. However, in the case of the *trans*-*trans* variant of D8.21, the symmetry and the more open conformation with fewer NOEs did not give a unique conformational solution. In these cases, the conformation that best correlated the temperature coefficients was selected.

We synthesized 67 macrocycles spanning two 6-mers, two 7-mers, fourteen 8-mers, twenty-five 9-mers, seventeen 10-mers, five 11-mers, and two 12-mers predicted to have alternative low-energy states. Out of these, 50 macrocycles have PAMPA P_app_ greater than 1 × 10^−7^ cm/s, and 25 show significant apparent permeability (P_app_ > 1 × 10^−6^ cm/s) (Figure 2A; Data S3). 9 out of the 10 designs tested in Caco-2 permeability assays show significant permeability: four 8-mers, two 9-mers, one 10-mer, and two 11-mers show P_app_ greater than 1 × 10^−6^ cm/s. Designs D8.21 and D8.33 show Caco-2 P_app_ greater than 1 × 10^−5^ cm/s (Figure 2B). We selected 19 peptides that showed significant permeability for further studies with NMR. The lower success rate in these designs is consistent with their greater polarity, the challenges in designing multiple states, and possibly much slower membrane transversal rates limited by the kinetics of conformational isomerization. 1D ^1^H NMR in d_6_-DMSO and CDCl_3_ indicated that the equilibrium between the structured conformations was strongly solvent dependent for seven of these designs, with switching between conformational states, or ratios of conformational states, between polar and nonpolar solvents. Energy landscape calculations also revealed that switch design generally resulted in more than two discrete low-energy states—an expected consequence of introducing interactions that favor more than one state. We clustered the low-energy ensembles from structure prediction calculations for each macrocycle and assigned each distinct minimum a state (cluster) identifier ranked on the lowest energy structure in that cluster. For three of these seven macrocycles (D8.21, D8.31, and D9.16) designed with the first approach, we succeeded in solving crystal structures matching the design model or one of the predicted alternate low-energy structures (Figure 4). We then carried out more detailed solution NMR studies of these designs using 2D NOESY, ROESY, TOCSY, ^13^C-^1^H HSQC, and ^13^C-^1^H TOCSY-HSQC experiments in CDCl_3_, d_6_-DMSO, and a 50:50 d_6_-DMSO/H_2_O mixture (see STAR Methods), assigning the prolines and N-methylated amino acids in each conformation as either *trans* or *cis* peptide conformations. The heteronuclear NMR studies were conducted using ^1^H detection of natural abundance ^13^C and ^15^N nuclei. These NMR data were then analyzed to determine structures of multiple states, simultaneously, of each peptide, providing a total of 11 solution NMR structures for three peptides in various solvents (Figures 4 and S6; Tables S3 and S4).

**Figure S7:**
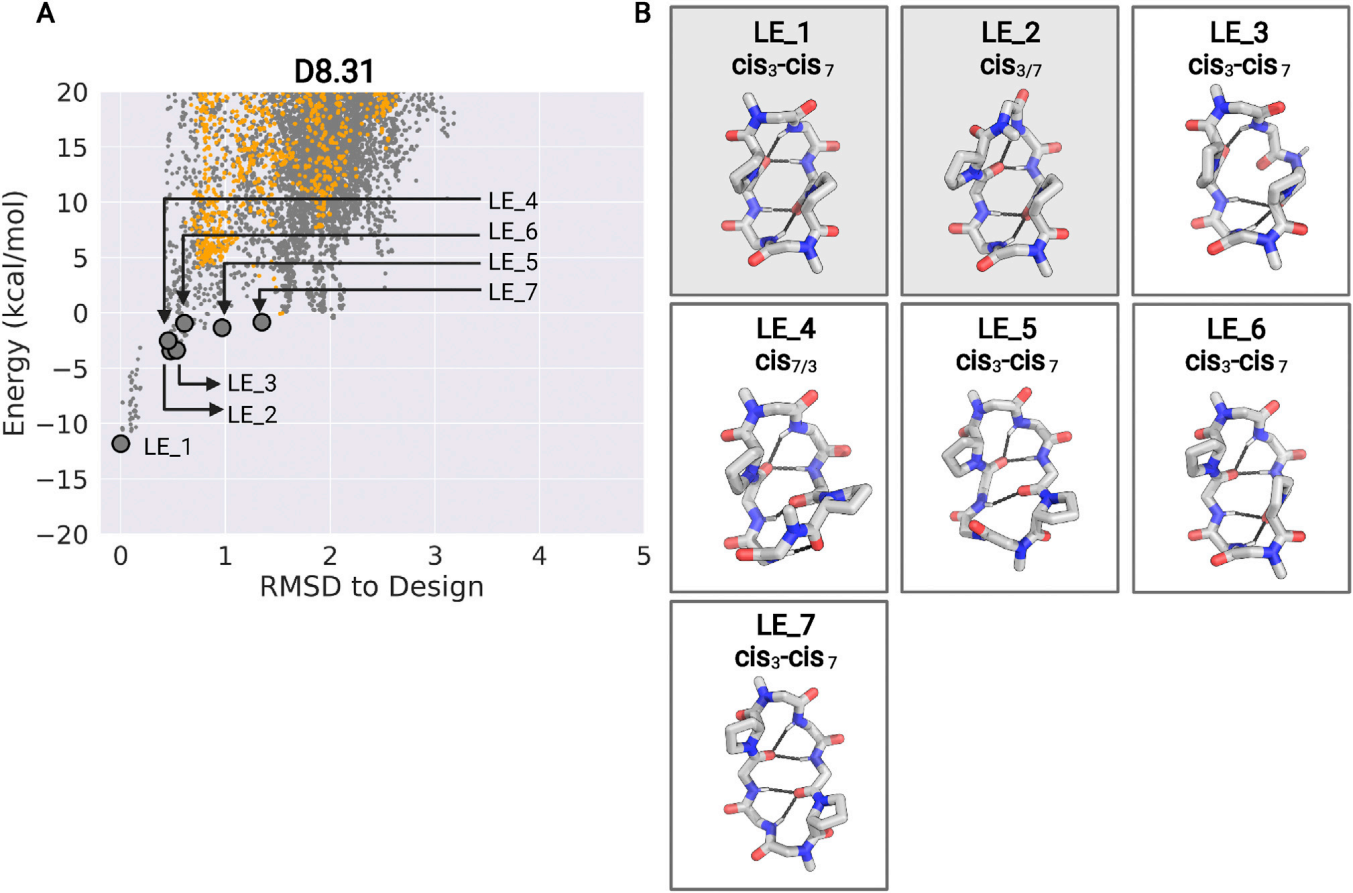
Low-energy structural clusters for design D8.31, related to Figure 4 (A) The 250 lowest energy predicted structures for D8.31 were selected and clustered using the Rosetta energy_based_clustering application. The cluster naming (LE_X) is based on the ranking of the lowest energy member from each cluster. The lowest energy structure from each identified cluster is labeled on the energy versus RMSD plot. Orange points: predicted structures with no *cis*-peptide bond; Gray points: predicted structures with at least one *cis*-peptide bond. (B) Lowest energy member from each cluster is shown in the stick representation. Position of *cis*-peptide bonds indicated in labels. Side chains for non proline (or D-proline) positions are not shown for clarity. The boxes with gray background denote the structures that match the X-ray crystal or the NMR structures.

Peptide D8.31 is an 8 amino acid macrocycle with a symmetric repeat sequence (ap^∗^lvap^∗^lv, ^∗^ represents N-methylated amino acids); the lowest energy state (LE_1) is C2 symmetric with both N-methylated amino acids in *cis* peptide bond conformations (“*cis*-*cis*”), and the second-lowest energy alternative state (LE_2) is asymmetric with one N-methylated leucine in the *cis* conformation (“*cis*-*trans*”) (Figure 4, left panel; Figures S6 and S7). The *cis*-*trans* isomerization occurs around an N-methylated amino acid; hence, both states have no unsatisfied NH groups. The crystal structure in the ethyl acetate:pentane solution is similar to the *cis*-*cis* LE_1. In d_6_-DMSO solution, two structures are present in slow exchange, a *cis*-*cis* conformation (∼50%) similar to LE_1, and *cis*-*trans* conformation (∼50%) similar to the LE_2 (Figure 4, left panels). In nonpolar CDCl_3_ solution, two conformations are also observed, with the dominant form (∼70%) matching the LE_1 state (Figure 4, left panels). The correspondence between these experimental X-ray crystal structures, solution NMR structures, and the predicted low-energy states for D8.31 demonstrates that Rosetta calculations can guide the design of macrocycles adopting multiple states. However, as the two different states have the same number of exposed NHs, these data do not directly address the contribution of conformational switching to membrane permeability. More relevant are the two other macrocycles, D8.21 and D9.16.

**Figure S8:**
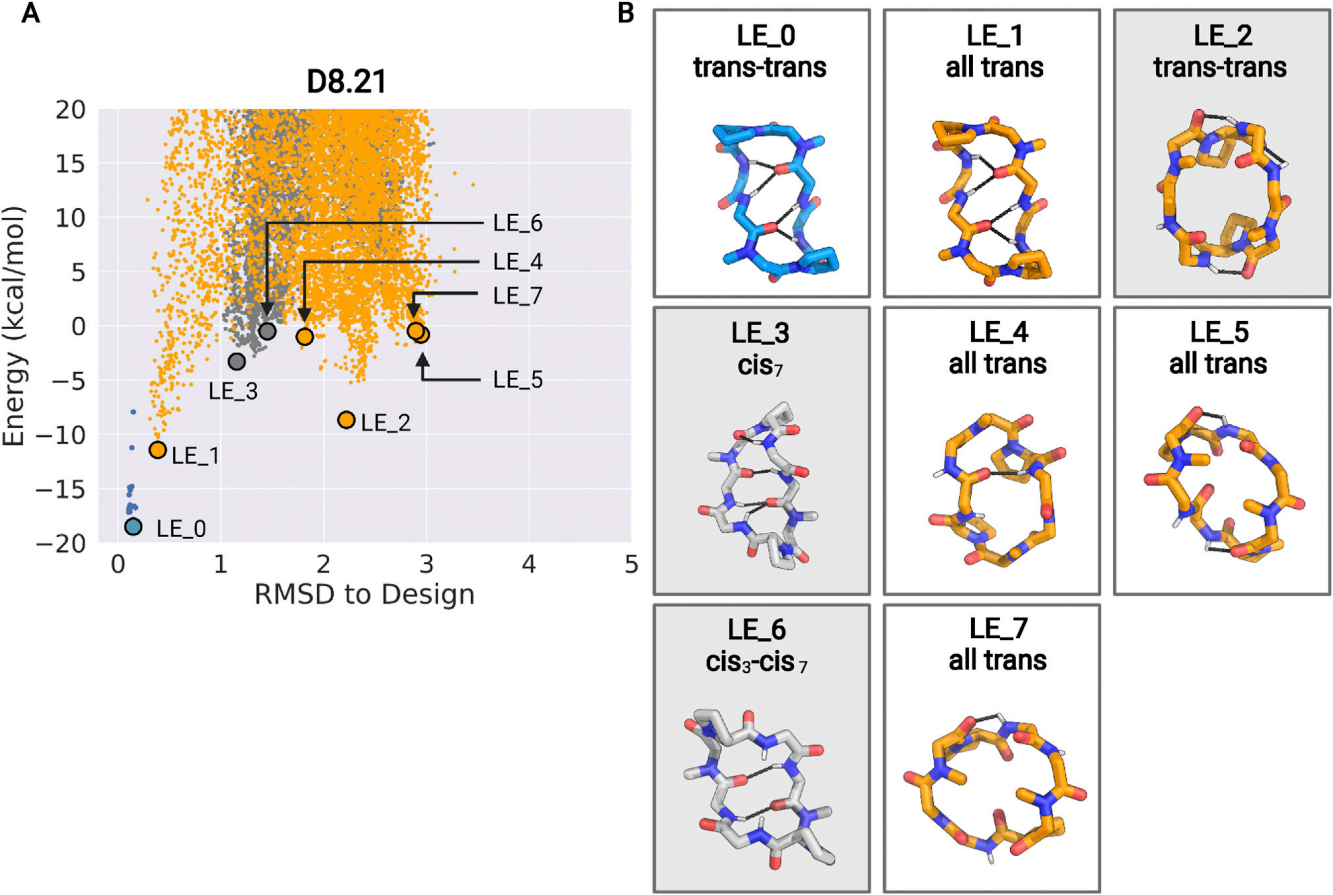
Low-energy structural clusters for design D8.21, related to Figure 4 (A) The 500 lowest energy predicted structures for D8.21 were selected and clustered using the Rosetta energy_based_clustering application. The cluster naming (LE_X) is based on the ranking of the lowest energy member from each cluster. The lowest energy structure from each identified cluster is labeled on the energy versus RMSD plot. Orange points: predicted structures with no *cis*-peptide bond; gray points: predicted structures with at least one *cis*-peptide bond; blue points: structures obtained after local minimization of the design model (LE_0). (B) Lowest energy member from each cluster is shown in the stick representation. The color of stick representation is based on the presence or absence of any *cis*-peptide bond in the structure. Orange: structures with no *cis*-peptide bonds; gray: structures with at least one *cis*-peptide bond; blue: design model. Position of *cis*-peptide bonds indicated in labels. Side chains for non proline (or D-proline) positions are not shown for clarity. The boxes with gray background denote the structures that match the X-ray crystal or the NMR structures.

**Figure S9:**
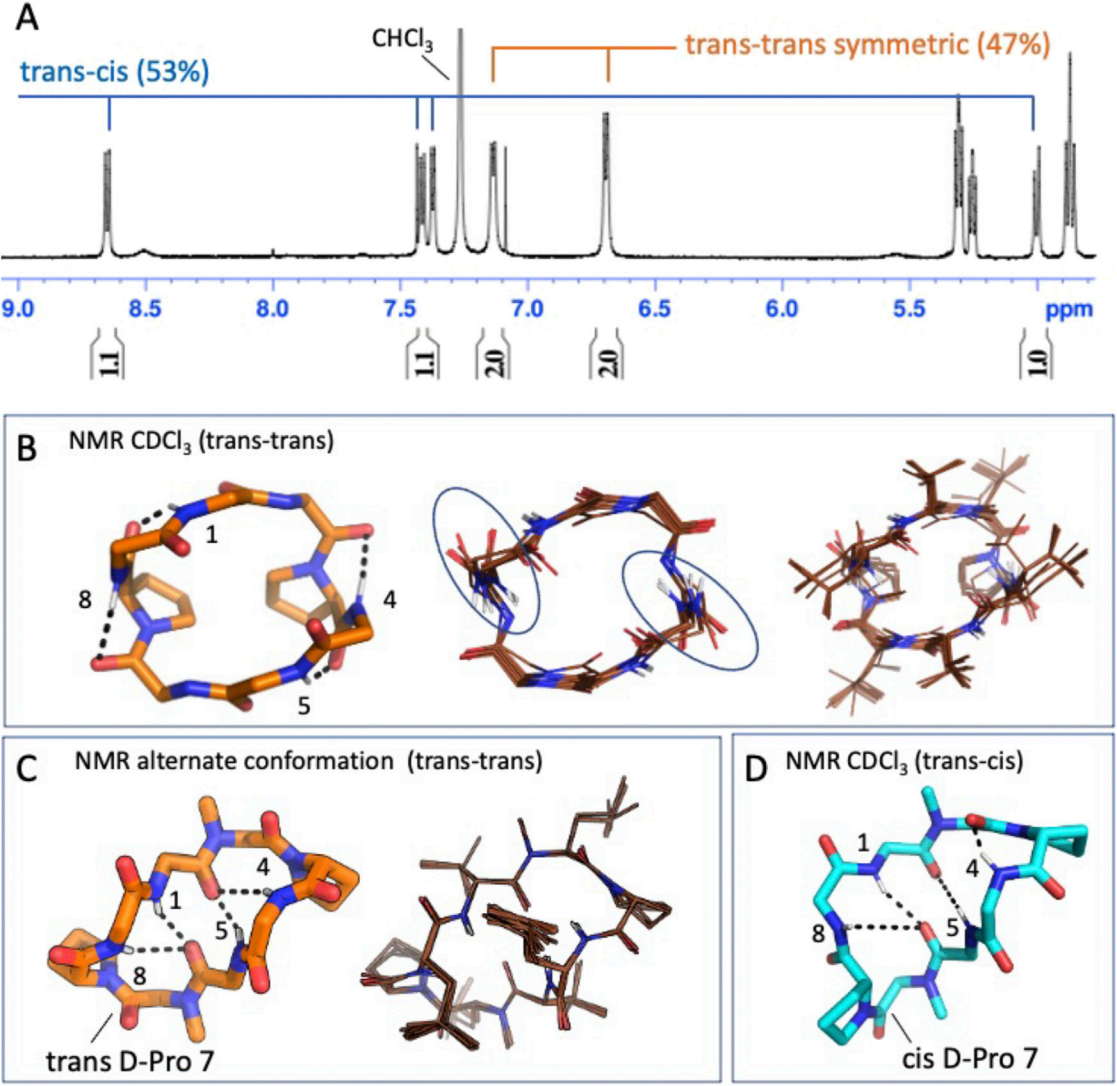
NMR structures of D8.21 have conformational ambiguity, related to Figure 4 (A) 1D ^1^H NMR spectrum of D8.21 in CDCl_3_ collected at 600 MHz and 293 K. Amide ^1^H peaks for each conformation are indicated. (B and C) (B) The *trans*-*trans* NMR structure (47% population) in CDCl_3_ that best matches the NMR data. It has an “open” conformation with only surface hydrogen bonds and buried but unsatisfied NHs and is similar to the *trans*-*trans* conformation observed in DMSO and DMSO-water. A representative member of this ensemble (left panel) shows four surface hydrogen bonds, whereas the ensemble of 20 structures (backbone—middle panel; backbone plus sidechain—right panel) shows the variability in orientations of the NH donor and carbonyl acceptor conformations along with buried, unsatisfied NHs. (B) An alternate “closed” *trans*-*trans* conformation. In order to assess whether the NMR data obtained for D8.21 in CHCl_3_ could possibly be fit to a more closed conformation with buried hydrogen bonds, we also used Cyana to reassign the ROESY data subject to restraints imposed for the four specific hydrogen bonds observed in the “closed” *trans*-*cis* conformation (47% in CHCl_3_), which are also observed in the designed and predicted low-energy *trans*-*trans* state (design model and LE_1 in Figure S8). Although the open *trans*-*trans* conformation (B) fits the NMR ROESY and amide temperature coefficient data better than the closed conformation, the alternate closed *trans*-*trans* conformation (C) cannot be ruled out. This ambiguity is due primarily to the chemical shift degeneracy that results from the repetitive 4-residue sequence (v^∗^LpL)_2_ and the symmetry of this conformation. The assignment of ROEs between degenerate/symmetric chemical shifts has multiple possibilities. For example, the assignment of ROEs to short distances between D-Pro 3 and D-Pro 7 rings in the open conformation is ambiguous because they are indistinguishable from intraresidue D-proline peaks. For the closed conformation, the D-Val 1 and 5 HNs have a characteristic short distance that is not observable in the ROESY data due to their degenerate chemical shifts. The alternative assignments of the ROEs by Cyana are based on preliminary structures and can be influenced by one or two manual restraints guiding it toward one or the other conformation. (D) The *trans-cis* conformation determined from NMR data in CHCl_3_ (53% population).

Macrocycle D8.21 also has a symmetric repeat sequence (v^∗^LpLv^∗^LpL) with predicted low-energy “*trans*-*trans*” states (2 variants, LE_1 and LE_2), “*trans*-*cis*” states (LE_3), and “*cis*-*cis*” (LE_6) states (Figure S8). The *trans*-*trans* LE_2 state has exposed NH groups, as well as two NHs forming surface-exposed hydrogen bonds. Both the *trans*-*cis* LE_3 and *cis*-*cis* LE_6 states have saturated NH groups that form hydrogen bonds in the core of the structure. The X-ray crystal structure of D8.21 under aqueous conditions is a *cis*-*cis* confomation similar to that of LE_6 (Figure 4, middle panel). In d_6_-DMSO solution, the peptide is a single conformationally symmetric *trans*-*trans* conformation similar to LE_2, while in 50:50 d_6_-DMSO/^2^H_2_O, it is a 50:50 equilibrium mixture of this same *trans*-*trans* conformer (LE_2) and a *cis*-*cis* conformation similar to both the X-ray crystal structure and LE_6 (Figure 4, middle panel). In CDCl_3_, the peptide also adopts two conformational states in equilibrium; the LE_2 symmetric *trans*-*trans* conformation with ∼47% population (see Figure S9C and legend for possible alternative state), and an asymmetric *cis*-*trans* structure with all of its NHs satisfied, matching LE_3 with ∼53% population (Figure 4, middle panel; Figures S7 and S9). The degree of solvent exposure of NH groups observed in these NMR structures is also generally consistent with temperature dependence of amide chemical shift data (Figure S6), and we observe significant stabilization of the *trans*-*cis* LE_3 conformer with all NHs satisfied in nonpolar CDCL_3_ compared with more polar solvent. Stabilization of LE_3 with NH groups in internal hydrogen bonds in more nonpolar solvent drives conformational switching, increasing the relative population of this state from 0% in a polar solvent to 53% in a nonpolar solvent while reducing the population of the LE_2 conformer with unsatisfied surface NH groups from 100% in polar DMSO (and ∼50% in 50:50 d_6_-DMSO/H_2_O) to less than 50% population in CDCl_3_.

**Figure S10:**
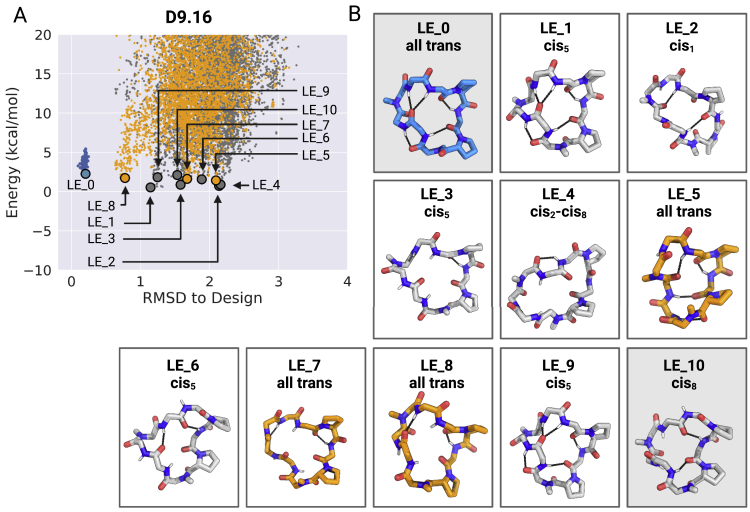
Low-energy structural clusters for design D9.16, related to Figure 4 (A) The 250 lowest energy predicted structures for D9.16 were selected and clustered using the Rosetta energy_based_clustering application. The cluster naming (LE_X) is based on the ranking of the lowest energy member from each cluster. The lowest energy structure for 10 lowest energy clusters is labeled on the energy versus RMSD plot. Orange points: predicted structures with no *cis*-peptide bond; gray points: predicted structures with at least one *cis*-peptide bond; blue points: structures obtained after local minimization of the design model (LE_0). (B) Lowest energy member from each of the 10 lowest energy clusters is shown in the stick representation. The color of stick representation is based on the presence or absence of any *cis*-peptide bond in the structure. Orange: structures with no *cis*-peptide bonds; gray: structures with at least one *cis*-peptide bond; blue: design model. Position of *cis*-peptide bonds indicated in labels. Side chains for non proline (or D-proline) positions are not shown for clarity. The boxes with gray background denote the structures that match the X-ray crystal or the NMR structures.

Design D9.16 has two N-methylated amino acids and two prolines (p^∗^AAv^∗^LLLPl). The low-energy design model is a “*trans*-*trans*” conformation (LE_0) with no unsatisfied NH groups. The predicted low-energy states include a “*trans*-*cis*” state (LE_10) with exposed NH groups (Figure 4, right panel; Figures S6 and S10). The X-ray crystal structure from aqueous conditions is in a *trans*-*cis* conformation that matches LE_10. In polar solvents (i.e., 50:50 d_6_-DMSO/^2^H_2_O and 100% d_6_-DMSO), the peptide has a single *trans*-*cis* conformation, with two exposed and one buried unsatisfied NH groups, that matches LE_10 (Figure 4, right panel). NMR data in nonpolar solvent (CDCl_3_) reveal an equilibrium between two states: a *trans*-*cis* state that matches the LE_10 state observed in both d_6_-DMSO and the crystal structure, and a *trans*-*trans* state with all backbone NHs satisfied that matches closely with the designed *trans*-*trans* state LE_0 (Figure 4, right panel). The population of the *trans*-*cis* LE_10 with exposed NH groups changes from 100% in a polar solvent to 45% in a nonpolar solvent, while the population of *trans*-*trans* LE_0 with all NH groups satisfied switches from 0% in a polar solvent to 55% in a nonpolar solvent.

Taken together, these data indicate that the D8.21, D8.31, and D9.16 macrocycles indeed populate multiple states, with the low-energy states closely matching the experimental crystal and NMR structures. Consistent with our conformational switch design strategy, the relative populations of the different states observed for D8.21 and D9.16 are solvent dependent, with the state having less exposed or no unsatisfied NH groups favored in the more nonpolar solvent. Overall, the solvent change shifts the equilibrium between the low-energy conformations of these chameleonic macrocycles. The macrocycles are also, as intended by design, membrane permeable, but due to difficulties in characterizing the state of macrocycles during membrane traversal, we cannot attribute this permeability specifically to one of the designed states. Boding well for the future design of membrane-permeable macrocycles targeting polar binding sites, it is notable that both D8.21 and D9.16 expose backbone NHs in one state, yet retain significant permeability.

### Oral bioavailability

**Figure 5:**
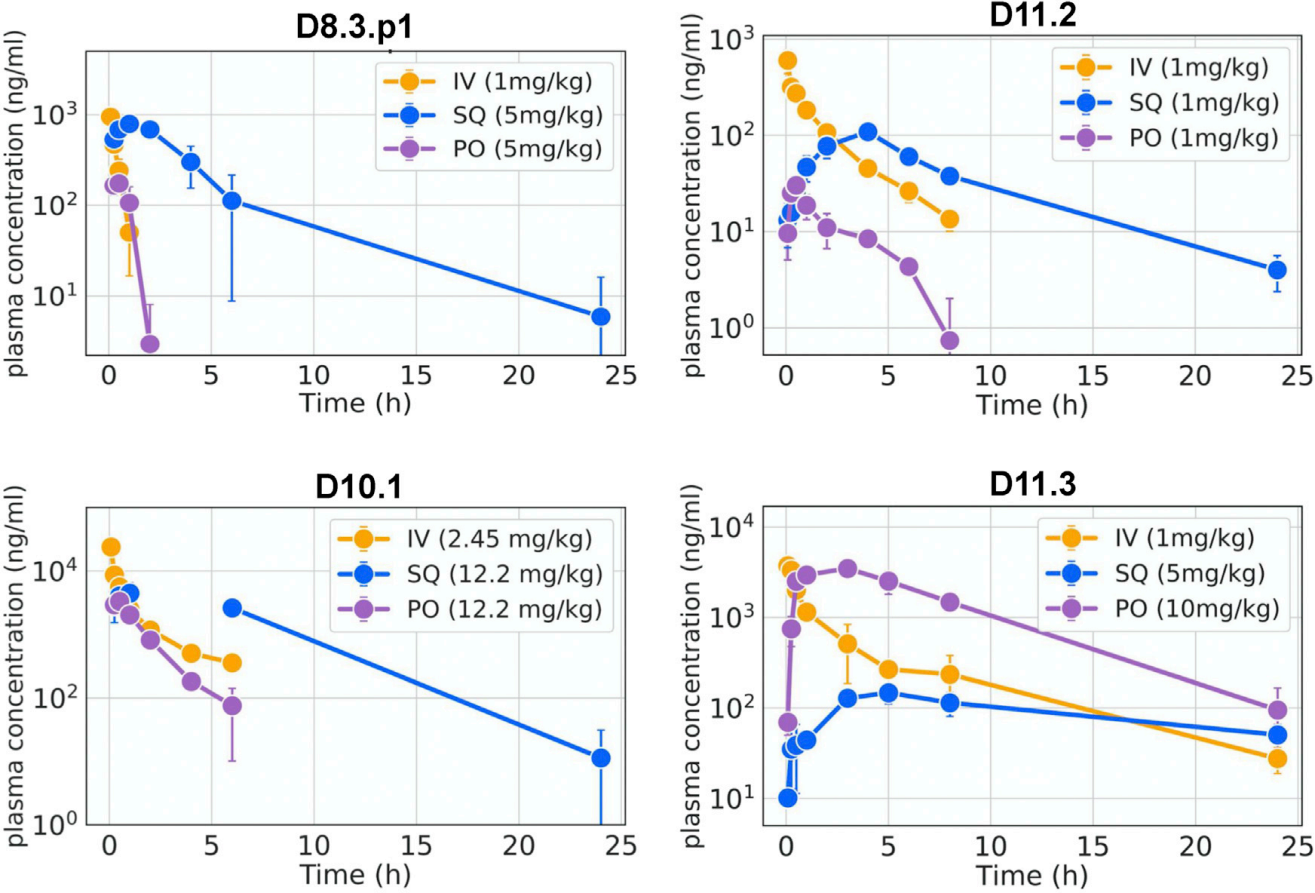
Designed macrocycles are orally bioavailable *in vivo* in rodent models Plasma concentration of unmodified full-length peptides measured after intravenous (IV), subcutaneous (SQ), and oral (PO) administration in mice (D8.3.p1, D10.1, and D11.3) and rats (D11.2) (n = 3 mice per dosing route for D8.3.p1, D10.1, and D11.3 and n = 3 rats per dosing route for D11.2). D8.3.p1 and D10.1 were studied in female BALB/c mice, D11.2 was studied in male Sprague Dawley (SD) rats, and D11.3 was studied in male swiss albino mice. See also Data S5.

Oral bioavailability is a desirable therapeutic property that requires stability against the low pH and proteases in the gastrointestinal tract and permeation across the epithelial cells in the gut. We selected four macrocycles covering different sizes and rates of *in vitro* permeability for *in vivo* oral bioavailability and pharmacokinetic studies in rodent models. Plasma exposure for one 8-mer (D8.3.p1), one 10-mer (D10.1), and two 11-mer peptides (D11.2 and D11.3) was measured after single-dose administration via intravenous (IV), subcutaneous (SQ), and oral (PO) routes. The amount of unmodified drug in plasma was quantified by mass spectrometry and the fraction of unmodified drug (%F) in plasma after oral delivery was determined using IV dosing as reference (see STAR Methods). The peptides were well tolerated without any adverse effects at the doses tested. All four designs had substantial oral exposure and demonstrated comparable or better oral bioavailability than most other natural orally absorbed peptides. Designs D8.3.p1, D11.2, and D10.1 have a good %F between 7.5% and 11% (Figure 5; Data S4). The 11-mer design, D11.3, was tested for oral bioavailability in male Swiss albino mice and had a very high oral bioavailability (%F) of 40% despite its large size. The designs also demonstrated other favorable drug-like attributes, such as long plasma half-life (T_1/2_). D11.3 has a T_1/2_ of 5.58 h after IV dosing, and D10.1 has a T_1/2_ of 3.75 h after SQ administration (Figure 5; Data S4). Overall, these *in vivo* data validate that these computationally designed and structurally validated peptides are robust to low pH and protease exposure and get absorbed efficiently across the gut epithelial barrier.

## Discussion

We have shown that the ability to precisely control the structure enables the robust design of a wide diversity of membrane-permeable macrocycles that goes considerably beyond those discovered previously, primarily N-methylated beta hairpins (Bockus et al., 2015; Fouché et al., 2016; Hill et al., 2014; Ovadia et al., 2011; Wang et al., 2014). Like natural products, our designs achieve this high permeability through conformational shielding of polar groups using a diversity of local structures and internal hydrogen bonds. In total, we designed, synthesized, and validated 84 structurally diverse macrocycles with good to excellent permeability, including 6–8 residue macrocycles with high permeability and no N-methyl groups, and 9–12 residue membrane-permeable macrocycles with a single N-methylated amino acid in the sequence. The passive permeability of the designs in PAMPA translates to good permeability across Caco-2 cells and oral bioavailability in rodent models. The importance of computational-design-based control over structure is highlighted by the strong correlation between the extent of permeability and sub-angstrom match between experimental structure and design model: of the 35 designs for which we succeeded in determining crystal or NMR structures, 21 macrocycles out of the 25 that closely matched (RMSD < 1 Å) the design states were all membrane permeable (P_app_ > 1 × 10^−7^ cm/s). While the very close agreement (RMSD < 1.2 Å) between the models of 29 out of the 35 designs and the corresponding experimental crystal or NMR structures indicates that the design method has very high accuracy, we cannot exclude the possibility that designs for which we lack X-ray or NMR structures fold into alternate conformations important for permeability.

The design methods and membrane-permeable macrocycles presented in this paper provide the basis for the development of orally bioavailable macrocyclic therapeutics. The ability to precisely control structure should enable the targeting of a wide range of binding site geometries, and the ability to robustly design for membrane permeability should allow access to intracellular targets and oral delivery. Our energy landscape-based approach to designing peptides that exhibit cyclosporine-like chameleonic behavior, switching between a lipophilic state and a second, relatively polar, state as solvent polarity changes, should enable the design of macrocycles that can bind intracellular therapeutic targets with exposed polar groups while retaining membrane permeability.

### Limitations of the study

While there is a good match between the X-ray crystal structures, NMR structures, and permeability data, it cannot be ruled out that designs have alternate conformations that affect their permeability but did not crystallize. While we focus here on achieving membrane permeability through intrapeptide hydrogen bonding, sidechain-mediated shielding of unsatisfied NH groups could also be effective; the structurally validated macrocycles provide templates to systematically evaluate the effects of NH shielding on permeability. We do not yet have as precise control over the chameleonic peptide alternative structures as we do with the single-state designs. This should improve with increases in energy function precision and improvements in landscape sampling, both methodological and through increases in available computing power.

The key challenge going forward is incorporating target binding functionality into the designs while maintaining control over structure and permeability. In this paper, the design calculations were focused on optimizing folding precision and permeability. Designing binders may require the incorporation of polar or other functional groups that make energetically favorable interactions with the target but may not be optimal for folding or permeability (or both). Our current efforts are focused on addressing this challenge by investigating the effects of polar side chains on permeability, and more generally, by designing and characterizing binders for a variety of intracellular targets.

## STAR★Methods

### Key resources table

**Table:** 

REAGENT or RESOURCE	SOURCE	IDENTIFIER
**Chemicals, peptides, and recombinant proteins**		

Cl-TCP(Cl) ProTide resin	CEM	R005
Fmoc amino acids	P3 biosystems	N/A
N,N-Dimethylformamide	Fisher	D119
Piperidine	Millipore Sigma	104094
Trifluoroacetic acid	Millipore Sigma	T6508
Dichloromethane	Fisher	D151
PyAOP	Millipore Sigma	8512210025
N,N-Diisopropylethylamine	Millipore Sigma	D125806
2-Propanol	Fisher	A451
d6-DMSO with 0.03% TMS	Krackeler	CAS # 2206-17-1
chloroform-d with 0.03% (v/v) TMS	Krackeler	CAS #865-49-6
Pre-coated PAMPA plates	Corning	353015
DMEM/F12 medium	Gibco	11320033
RPMI medium	Gibco	11875119
alamarBlue cell viability reagent	Invitrogen	DAL1025
HEPES	Gibco	15630080
L-glutamine	Gibco	25030081
Trypsin-EDTA (0.25%), phenol red	Gibco	25200056
Sodium pyruvate	Gibco	11360070
Fetal Bovine Serum	VWR Seradigm	97068-085
Microvette 100 w/ capillary straw, 100 μL Lithium heparin	SAI infusion technologies	MVC-H-100
Tween 80	Sigma-Aldrich	P8074
Dimethyl sulfoxide	Sigma-Aldrich	D2650
PEG 400	Sigma-Aldrich	PX1286B
DPBS	Gibco	14190136
Dextrose	Sigma-Aldrich	D9434
Transwell® polyester membrane cell culture inserts	Corning	CLS3460
DMEM - Dulbecco's Modified Eagle Medium	Gibco	11965118
Fetal Bovine Serum	Gibco	10099141
Penicillin-Streptomycin	Gibco	15070063
MEM Non-Essential Amino Acids Solution	Gibco	11140050
Puromycin dihydrochloride	Sigma-Aldrich	P9620
Hank's Balanced Salt Solution (HBSS)	Gibco	14175079
Atenolol	Sigma-Aldrich	A7655
Quinidine	Sigma-Aldrich	Q3625

**Deposited data**		

D7.6 X-ray crystal structure	This paper	CCDC 131411
D7.8 X-ray crystal structure	This paper	CCDC 2131412
D8.1 X-ray crystal structure	This paper	CCDC 2131449
D8.2 X-ray crystal structure	This paper	CCDC 2131417
D8.3.p1 X-ray crystal structure	This paper	CCDC 2131450
D8.5.p2 X-ray crystal structure	This paper	CCDC 2131423
D8.6 X-ray crystal structure	This paper	CCDC 2131424
D8.9 X-ray crystal structure	This paper	CCDC 2131249
D8.10 X-ray crystal structure	This paper	CCDC 2131251
D8.12 X-ray crystal structure	This paper	CCDC 2131252
D8.13 X-ray crystal structure	This paper	CCDC 2131463
D8.14 X-ray crystal structure	This paper	CCDC 2131425
D8.15 X-ray crystal structure	This paper	CCDC 2131426
D8.17 X-ray crystal structure	This paper	CCDC 2131427
D8.19 X-ray crystal structure	This paper	CCDC 2131428
D8.25 X-ray crystal structure	This paper	CCDC 2131253
D8.26 X-ray crystal structure	This paper	CCDC 2131291
D9.1 X-ray crystal structure	This paper	CCDC 2131429
D9.8 X-ray crystal structure	This paper	CCDC 2131430
D9.24 X-ray crystal structure	This paper	CCDC 2131245
D9.29 X-ray crystal structure	This paper	CCDC 2131431
D9.30 X-ray crystal structure	This paper	CCDC 2131432
D10.1 X-ray crystal structure	This paper	CCDC 2131433
D10.21 X-ray crystal structure	This paper	CCDC 2131434
D10.22 X-ray crystal structure	This paper	CCDC 2131435
D10.23 X-ray crystal structure	This paper	CCDC 2131436
D10.31 X-ray crystal structure in IPA: water	This paper	CCDC 2131438
D10.31 X-ray crystal structure in Ethyl Acetate: Pentane	This paper	CCDC 2131437
D10.65 X-ray crystal structure	This paper	CCDC 2131439
D11.1 X-ray crystal structure	This paper	CCDC 2131440
D11.3 X-ray crystal structure	This paper	CCDC 2131451
D11.4 X-ray crystal structure	This paper	CCDC 2131441
D11.25 X-ray crystal structure	This paper	CCDC 2131442
D8.31 X-ray crystal structure	This paper	CCDC 2131292
D8.21 X-ray crystal structure	This paper	CCDC 2131443
D9.16 X-ray crystal structure	This paper	CCDC 2131293
D8.3.p2 X-ray crystal structure	This paper	PDB 7UCP
D9.16 in d6-dMSO (trans-cis) solution NMR structure	This paper	PDB 7UBC & BioMagResDB 30997
D8.31 in d6-DMSO (cis-cis) NMR structure	This paper	PDB 7UBD & BioMagResDB 30998
D8.31 d6-DMSO (cis-trans) NMR structure	This paper	PDB 8CTO & BioMagResDB 31021
D8.21 in d6-DMSO (trans-trans) NMR structure	This paper	PDB 7UBE & BioMagResDB 30999
D8.21 in 50:50 d6-DMSO/H2O (trans-trans) NMR structure	This paper	PDB 7UBF & BioMagResDB 31000
D8.21 in 50:50 d6-DMSO/H2O (cis-cis) NMR structure	This paper	PDB 8CUN & BioMagResDB 31022
D9.16 in CDCl3 (trans-trans) NMR structure	This paper	PDB 7UBG & BioMagResDB 31001
D9.16 in CDCl3 (trans-cis) NMR structure	This paper	PDB 7UZL & BioMagResDB 31019
D8.31 in CDCl3 (cis-cis) NMR structure	This paper	PDB 7UBH & BioMagResDB 31002
D8.21 in CDCl3 (trans-trans) NMR structure	This paper	PDB 7UBI & BioMagResDB 31003
D8.21 in CDCl3 (trans-cis) NMR structure	This paper	PDB 8CWA & BioMagResDB 31023

**Experimental models: Cell lines**		

Hep G2 human hepatocellular carcinoma cell line	ATCC	HB-8065
WIL2-NS human B lymphocyte cell line	ATCC	CRL-8155
CACO-2 Cell Line human	CellBank Australia	86010202

**Experimental models: Organisms/strains**		

10-12 week old female BALB/cAnNCrl mice	Charles River	strain code 028
Male JVC Sprague Dawley rats	Hilltop Lab animals incorporated	http://www.hilltoplabs.com/

**Software**		

Rosetta and PyRosetta Scripts for Macrocycle Design	This study	Data S5
TopSpin 3.5 and 4.0.9	Bruker BioSpin, Inc	www.bruker.com
Cyana 3.98.13	Güntert et al., 1997	https://www.las.jp/english/products/cyana.html
NMRFAM-SPARKY	Lee et al., 2015	https://nmrfam.wisc.edu/nmrfam-sparky-distribution/
NMRPipe v10.9	Delaglio et al., 1995	https://www.ibbr.umd.edu/nmrpipe/
PDBStat v5.21.6	Tejero et al., 2013	http://rtti7.uv.es/∼roberto/Index.php?sec=pdbstat
Phoenix WinNonlin®, Version 6.3	Pharsight Corp	https://www.certara.com/software/phoenix-winnonlin/
XDS	Winn et al., 2001	https://xds.mr.mpg.de/
CCP4	Kabsch, 2010	https://www.ccp4.ac.uk/
SHELXL	Sheldrick, 2015a, 2015b	https://shelx.uni-goettingen.de/
SHELXLe	Hübschle, 2011	https://www.shelxle.org/shelx/eingabe.php

**Other**		

Bruker Avance II 600 MHz NMR System	Bruker Biospin, Inc.	https://www.bruker.com/en.html
Bruker Avance III 600 MHz NMR System	Bruker Biospin, Inc.	https://www.bruker.com/en.html
Bruker Avance II 800 MHz NMR System	Bruker Biospin, Inc.	https://www.bruker.com/en.html

### Resource availability

#### Lead contact

Further information and requests for resources and reagents should be directed to and will be fulfilled by the lead contact, David Baker (dabaker@uw.edu).

#### Materials availability

All the codes and data used in the manuscript are available in supplemental information files. Other materials are available upon request.

### Experimental Model and Subject Details

#### Caco-2 assays

Human colon adenocarcinoma-derived Caco-2 cell line was used as a model system to investigate the intestinal epithelial permeability of our designs. Caco-2 cells were propagated in T125 flasks (Corning, Merck) using Dulbecco’s modified Eagle medium supplemented with 10% (v/v) fetal bovine serum, 1% (v/v) nonessential amino acids, and 1% (v/v) penicillin-streptomycin (Gibco, Thermo Fisher Scientific) at 37°C in an atmosphere of 5% CO2. Cells were subcultured when they reached 80% confluence.

#### *In vivo* bioavailability assays

In vivo bioavailability assays were conducted in mice and rats. D8.3.p1 and D10.1 were studied in female Balb/C mice by van Voorhis group at University of Washington. D11.2 was evaluated in Sprague Dawley (SD) rats by Robert Griffin’s group at Takeda Pharmaceuticals. D11.3 was studied in the male swiss albino mice by the contract research organization, GVK Biosciences Pvt. Ltd., Hyderabad, India. All animals used in these studies were handled in strict accordance with practices made to minimize suffering. All animal experiments conducted at the University of Washington, USA, and Takeda Pharmaceuticals, USA were approved by the Institutional Animal Care and Use Committee. Animal studies at GVK Biosciences were reviewed and approved by the Institutional Animal Ethics Committee of GVK Biosciences.

### Method Details

#### Enhancements to the Rosetta software suite

In order to allow the design, modeling, and structure prediction of cyclic peptides incorporating N-methylated amino acids in the Rosetta software suite, a number of enhancements were needed. First, Rosetta’s kinematic machinery, which keeps track of updating Cartesian coordinates of atoms as internal degrees of freedom (such as torsion angles) of a polymer change, was refactored. Code for generating ideal coordinates of atoms whose positions depend on peptide bonds (such as the carbonyl oxygen and amide proton) was altered to allow whole chemical groups (such as an N-methyl group) to be placed. Rosetta’s protein cutpoint code was also refactored. This code ensures that a special term in the Rosetta energy function (called chainbreak) penalizes poor peptide bond geometry when a peptide bond is located at a break in the kinematic tree, as inevitably happens at some point in a cyclic peptide; effectively, this keeps a cyclic peptide from opening during energy minimization. The needed changes made this term compatible with *cis*-peptide bonds and with N-methylation.

Rosetta’s residue type system is organized into residue types (distinct chemical entities) and patches (small variations on existing types). Chemical modifications such as a protonated N-terminus, a deprotonated C-terminus, or a methylated side-chain are typically handled with patches, which instruct Rosetta to add a variant by altering an existing residue type’s geometry, rather than by adding an entirely new residue type. We supported backbone N-methylation in the same way, adding a patch to convert an amino acid residue type to its N-methylated equivalent. However, the addition of a methyl group on the backbone nitrogen greatly alters both the backbone and side-chain conformational preferences of a given residue type. We added support to Rosetta’s patching system to permit new mainchain potentials and side-chain rotamer libraries to be specified for a patched residue type as part of the patching process. We then computed new mainchain Ramachandran potentials for N-methyl-glycine (sarcosine) and for N-methyl-L-alanine, using the AMBER GAFF force field for N-methyl-glycine and Gaussian quantum mechanics calculations with the B3LYP/6-311+G(d,p) level of theory for N-methyl-L-alanine. Since the steric repulsion of an N-methyl group on the i^th^ amino acid residue greatly influences the conformational preferences of the i-1^st^ amino acid residue in much the same way that the proline side-chain’s steric repulsion when proline is at the i^th^ position affects the conformational preferences of the i-1^st^ residue, Rosetta’s rama_prepro energy term (which computes the mainchain potential, and applies different Ramachandran potentials to residues preceding proline) was extended to apply the pre-proline Ramachandran potentials to positions preceding N-methylated positions as well. Pre-proline/pre-N-methyl Ramachandran potentials were similarly computed for N-methyl-glycine and N-methyl-L-alanine. Using the previously-described MakeRotLib protocol (Renfrew et al., 2012), rotamer libraries for the following N-methylated L-amino acid residues were generated: arginine, asparagine, aspartate, cysteine, glutamine, glutamate, histidine, isoleucine, leucine, lysine, methionine, phenylalanine, serine, threonine, tryptophan, tyrosine, and valine. Rosetta automatically mirrors mainchain potentials and rotamer libraries that were generated for L-amino acid residues when sampling conformations or computing energies of D-amino acid residues, and this functionality was extended to N-methylated amino acids. Building on past work to generalize Rosetta’s fa_dun sidechain potential to use rotamer probabilities computed with MakeRotLib when computing the energies of side-chain conformations (Mulligan et al., 2021), we removed special-case code that had previously been included for peptoid rotamer libraries (Renfrew et al., 2012), permitting all rotamer sampling and scoring to be handled by the newly-generalized rotamer code.

During testing, we found that the close interactions between an N-methyl group, the preceding carbonyl oxygen, and the preceding and adjacent side-chains resulted in frequent clashes that were highly sensitive to the rotation of the N-methyl group. In some cases, these clashes would result in Rosetta choosing distinctly sub-optimal side-chain rotamers during rotamer optimization (packing). We, therefore, added support for freely-rotating N-methyl groups, allowing precise positions of methyl hydrogen atoms to be adjusted during packing or gradient-descent energy minimization.

To facilitate conformational sampling during design and structure prediction, we modified the generalized kinematic closure (GeneralizedKIC) method described previously (Bhardwaj et al., 2016; Hosseinzadeh et al., 2017, Hosseinzadeh et al., 2021; Mulligan et al., 2021). Briefly, GenerlizedKIC-based peptide macrocycle conformational sampling works by building an extended chain, randomizing all but 6 backbone torsion angles, and then solving analytically for the values of the remaining 6 “pivot residue” torsion angles in order to ensure ideal peptide bond geometry at the breakpoint. In order to better bias sampling based on the conformational preferences of each amino acid, given its residue type and the presence or absence of a methyl group on its backbone nitrogen and on the backbone nitrogen of the next amino acid, we added a new GeneralizedKIC perturber, called randomize_backbone_by_rama_prepro, which uses the same Ramachandran potentials that are used for scoring to bias sampling. Since pivot residue torsion angles are determined analytically rather than being sampled from a biased distribution, a given kinematic closure solution may have backbone torsion values in any region of Ramachandran space. We therefore also added a GeneralizedKIC filter, called rama_prepro_check, which uses the precomputed Ramachandran potentials to discard solutions with pivot residues in poor regions of Ramachandran space. Because kinematic closure calculations and filtering are performed on raw vectors of degrees of freedom without manipulating full atomic-resolution models, they are extremely fast, allowing thousands of conformations to be sampled in seconds; downstream design steps in a design protocol occupy the majority of the computing time. Documentation for the GeneralizedKIC perturbers and filters may be found on the Rosetta help wiki at https://www.rosettacommons.org/docs/latest/scripting_documentation/RosettaScripts/composite_protocols/generalized_kic/GeneralizedKICperturber and at https://www.rosettacommons.org/docs/latest/scripting_documentation/RosettaScripts/composite_protocols/generalized_kic/GeneralizedKICfilter, respectively.

In addition, a new residue selector, called Unsat, was developed to permit the identification of amino acid residues with unsatisfied backbone hydrogen bond donors or acceptors in order to permit automatic N-methylation in the context of a RosettaScripts or PyRosetta design protocol. This selector is compatible with both asymmetric and symmetric structures. Documentation for the Unsat residue selector may be found in the Rosetta online documentation (https://www.rosettacommons.org/docs/latest/scripting_documentation/RosettaScripts/ResidueSelectors/ResidueSelectors#residueselectors_conformation-dependent-residue-selectors_unsatselector).

We added support for using N-methylated amino acids in Rosetta’s simple_cycpep_predict application, which is used for the prediction of peptide macrocycle structure from its amino acid sequence and for estimating the folding propensity of each designed peptide in a pool in order to rank and prioritize designs for synthesis. During peptide conformational sampling, simple_cycpep_predict uses the GeneralizedKIC method described above to sample closed conformations of a macrocycle rapidly, with the newly-implemented randomize_backbone_by_rama_prepro GeneralizedKIC perturber to bias sampling and the rama_prepro_check GeneralizedKIC filter to discard solutions with poor backbone conformations at pivot atoms. Rosetta’s energy_based_clustering application was also modified to allow it to cluster N-methylated peptides, and to include the carbon of the backbone methyl group as an atom used when aligning structures and computing RMSD values. These applications have documentation available on the Rosetta help wiki, at https://www.rosettacommons.org/docs/latest/structure_prediction/simple_cycpep_predict#full-inputs_additional-flags-for-n-methylated-amino-acids and at https://www.rosettacommons.org/docs/latest/application_documentation/analysis/energy_based_clustering_application, respectively. Testing of these changes revealed a small bug related to an uninitialized variable in the Lazaridus-Karplus term in the Rosetta energy function, which was also corrected.

As of October 2021 (Rosetta Git revision a9ab4ac590fd0a4e2def5739deaeab02c72c949d), these enhancements have all been incorporated into public releases of Rosetta, with the exception of the N-methylated rotamer libraries. Due to their size, these libraries are not distributed with Rosetta (but for the N-methyl tryptophan rotamer library, used for unit testing), but all are bundled with noncanonical amino acid libraries distributed separately. Extensive unit tests have also been added to ensure that energy calculations involving N-methylated amino acids are invariant to cyclic permutation or to mirroring. Rosetta source code and compiled binaries are made freely available to academic, government, and not-for-profit users, and can be licensed from the University of Washington for corporate and for-profit use. To download and use Rosetta, please visit https://www.rosettacommons.org/software/license-and-download.

#### Computational design of structured membrane-permeable peptides

We modified the Rosetta generalized kinematic closure (GeneralizedKIC) based macrocycle design protocol described previously (Bhardwaj et al., 2016; Hosseinzadeh et al., 2017, Hosseinzadeh et al., 2021; Mulligan et al., 2021) to enable design of 6 to 12 amino acid macrocycles capable of traversing the lipid membranes and cellular barriers. All steps for macrocycle design were implemented in RosettaScripts (Fleishman et al., 2011).

Briefly, we chose a macrocycle size and initiated design calculations by constructing a linear polyglycine backbone of the selected amino acid length using the Rosetta PeptideStubMover (Bhardwaj et al., 2016). In this work, we performed separate design runs for each macrocycle size between 6 to 12 amino acids. Next, we declared a bond between the ‘C’ atom from the last residue and ‘N’ atom of the first residue in the polyglycine peptide and set up the distance, angle, and dihedral constraints for N-to-C terminal cyclization using the Rosetta PeptideCyclizeMover mover (Hosseinzadeh et al., 2017). We selected one residue randomly as the ‘anchor residue’ and three additional residues as the ‘pivot residues’. The omega torsions for all residues were set to 180°; ɸ and ψ torsions for the anchor residues were randomly selected from a flat bottom mirror-symmetric Ramachandran table using the SetTorsion mover (Bhardwaj et al., 2016). Following, we used the GeneralizedKIC mover to identify cyclic polyglycine peptides from the linear peptides (Bhardwaj et al., 2016). Within the GenKIC mover, the ɸ and ψ dihedrals of the non-pivot and non-anchor residues were randomly drawn from a flat bottom mirror-symmetric Ramachandran table. The ɸ and ψ dihedrals for the pivot residues were calculated analytically by the kinematic closure algorithm (Bhardwaj et al., 2016; Coutsias et al., 2004, Mandell et al., 2009) to find a combination of dihedral angles that give N-to-C cyclic peptide backbones. The criteria for closure were further defined to include a minimum number of internal backbone-to-backbone hydrogen bonds. The number of internal hydrogen bonds required was based on the length of the macrocycle: a minimum of 1 internal hydrogen bond was required for 6-7 amino acids, 2 hydrogen bonds were required for 8-9 amino acids, and 3 internal hydrogen bonds were required for macrocycles with 10 or more amino acids. In cases where GenKIC returned multiple cyclic solutions, we selected the lowest energy solutions based on a custom Rosetta energy function that includes only the fa_rep, fa_atr, hbond_sr_bb, hbond_lr_bb, rama_prepro, and p_aa_pp score terms (Alford et al., 2017). For each successfully closed cyclic backbone, we used the Rosetta FastDesign mover to design an amino acid sequence that tried to minimize the overall energy of the macrocycle (Bhardwaj et al., 2016).

Since our goal was to design macrocycles that can traverse the lipid membranes passively, we removed NH groups not involved in hydrogen bonding by mutating the amino acids with such ‘unsatisfied’ backbone NH groups to their N-methyl variants. Residues with the unsatisfied NH groups were selected using the Unsat selector and mutated using the ModifyVariantType mover to their N-methylated versions. However, given the different torsional preferences of the N-methylated amino acids, it is possible that the mutations to N-methyl amino acids could expose NH groups from other amino acids as well. Therefore, we followed an iterative approach with three rounds of amino acid sequence design and N-methylation of exposed backbone NH groups in-between. In the first round of designs we used an energy function with upweighted (5X) backbone hydrogen bonding score terms to favor more internal backbone-to-backbone hydrogen bonds. For the second round of design, we used the standard Rosetta beta_nov16 weights with constraints (Alford et al., 2017; Park et al., 2016). In the third round of design, we also allowed for cartesian minimization. We only allowed hydrophobic amino acids during design, and only D-amino acids were allowed at residue positions with positive ɸ, and only L-amino acids were allowed at residues with negative ɸ values. For some runs, we also used the AddCompositionConstraintMover to limit the minimum and maximum number of allowed prolines, D-prolines, and some bulky hydrophobic amino acids in the designed peptides (Hosseinzadeh et al., 2017). Given the difficulty in synthesizing peptides with multiple N-methylated amino acids, we filtered the design models based on total number N-methylated amino acids in the design models and lack of any exposed NH groups in the final designed state using Rosetta SimpleMetrics (Adolf-Bryfogle et al., 2021).

For each selected size range, approximately 10^5^ designs were sampled. Design models were clustered based on the torsion bin strings calculated from the backbone dihedral angles as described previously (Hosseinzadeh et al., 2017). Next, we pursued structure prediction of lowest-energy scoring designs from diverse clusters using Rosetta simple_cycpep_predict application (Bhardwaj et al., 2016; Hosseinzadeh et al., 2017) as described previously (Hosseinzadeh et al., 2017). We evaluated the Energy vs. RMSD-to-Design plots (Figure S2) from structure prediction calculations and selected the structured and conformation-switching peptides based on the number of low-energy states. In cases where the structure prediction calculations identified conformations with lower energies (denoted by _LE) than the design model, the lower energy conformation was used as the model for structural comparisons.

Rosetta Macromolecular Modeling Suite can be downloaded from https://rosettacommons.org/software. Rosetta Documentation describing the details of RosettaScripts and components used in the protocol described above are available at https://new.rosettacommons.org/docs/latest/Home. An example folder with the scripts and example command-line flags can also be found in the Data S5. A sample RosettaScripts for designing a 10-amino acid peptide with N-methylated amino acids is provided below.

<ROSETTASCRIPTS>

#Define scorefunctions to be used later in the script

<SCOREFXNS>

<ScoreFunction name="SFXN_STD" weights="beta_nov16.wts" >

<Reweight scoretype="atom_pair_constraint" weight="1" />

<Reweight scoretype="dihedral_constraint" weight="1" />

<Reweight scoretype="angle_constraint" weight="1" />

<Reweight scoretype="aa_composition" weight="1.0" />

</ScoreFunction>

<ScoreFunction name="SFXN_STD_high_hbond" weights="beta_nov16.wts" >

<Reweight scoretype="atom_pair_constraint" weight="1" />

<Reweight scoretype="dihedral_constraint" weight="1" />

<Reweight scoretype="angle_constraint" weight="1" />

<Reweight scoretype="aa_composition" weight="1.0" />

<Reweight scoretype="hbond_sr_bb" weight="5.0" />

<Reweight scoretype="hbond_lr_bb" weight="5.0" />

</ScoreFunction>

<ScoreFunction name="SFXN_CART" weights="beta_nov16_cart.wts" >

<Reweight scoretype="atom_pair_constraint" weight="1" />

<Reweight scoretype="dihedral_constraint" weight="1" />

<Reweight scoretype="angle_constraint" weight="1" />

<Reweight scoretype="aa_composition" weight="1.0" />

</ScoreFunction>

<ScoreFunction name="SFXN_hbond_bb" weights="empty.wts" symmetric="0">

<Reweight scoretype="fa_rep" weight="0.1" />

<Reweight scoretype="fa_atr" weight="0.2" />

<Reweight scoretype="hbond_sr_bb" weight="2.0" />

<Reweight scoretype="hbond_lr_bb" weight="2.0" />

<Reweight scoretype="rama_prepro" weight="0.45" />

<Reweight scoretype="omega" weight="0.4" />

<Reweight scoretype="p_aa_pp" weight="0.6" />

</ScoreFunction>

</SCOREFXNS>

<RESIDUE_SELECTORS>

#Define different residue selections to be used later in the script

<Chain name="chainA" chains="A"/>

<Chain name="sel_A" chains="1"/>

#select the regions with phi less than or greater than zero for designing with L- and D-amino acids

<Phi name="posPhi" select_positive_phi="true" />

<Phi name="negPhi" select_positive_phi="false" />

<Bin name="ProBin" bin="LPRO" bin_params_file="PRO_DPRO" />

<Bin name="DProBin" bin="DPRO" bin_params_file="PRO_DPRO" />

#we need to select residues that are outside +/-2 amino acids of each residue to calculate the internal h-bonds

<Index name="hbonds_to_1" resnums="4,5,6,7,8"/>

<Index name="hbonds_to_2" resnums="5,6,7,8,9"/>

<Index name="hbonds_to_3" resnums="6,7,8,9,10"/>

<Index name="hbonds_to_4" resnums="1,7,8,9,10"/>

<Index name="hbonds_to_5" resnums="1,2,8,9,10"/>

<Index name="hbonds_to_6" resnums="1,2,3,9,10"/>

<Index name="hbonds_to_7" resnums="1,2,3,4,10"/>

<Index name="hbonds_to_8" resnums="1,2,3,4,5"/>

<Index name="hbonds_to_9" resnums="2,3,4,5,6"/>

<Index name="hbonds_to_10" resnums="3,4,5,6,7"/>

#select the residues with unsatisfied NH grups

<Unsat name="unsat_amines" scorefxn="SFXN_STD" check_acceptors="false" consider_mainchain_only="false"/>

<And name="ProBin_unsat" selectors="ProBin,unsat_amines" />

<And name="DProBin_unsat" selectors="DProBin,unsat_amines" />

</RESIDUE_SELECTORS>

<SIMPLE_METRICS>

<SelectedResidueCountMetric name="count_unsats" residue_selector="unsat_amines" />

</SIMPLE_METRICS>

<TASKOPERATIONS>

<InitializeFromCommandline name="init"/>

<IncludeCurrent name="current"/>

<LimitAromaChi2 name="limitchi2" chi2max="110" chi2min="70" include_trp="True" />

### expanded chi1 and chi2 rotamer libraries

<ExtraRotamersGeneric name="ex1_ex2" ex1="1" ex2="1"/>

<ExtraRotamersGeneric name="ex1" ex1="1"/>

#Add the allowed residues from resfile that apply to selected regions of the peptide

<ReadResfile name="l_res" filename="l_res.txt" selector="negPhi"/>

<ReadResfile name="d_res" filename="d_res.txt" selector="posPhi"/>

<ReadResfile name="lpro_res" filename="pro.txt" selector="ProBin_unsat"/>

<ReadResfile name="dpro_res" filename="dpro.txt" selector="DProBin_unsat"/>

</TASKOPERATIONS>

<FILTERS>

#calculation of internal h-bonds in the peptide

<HbondsToResidue name="hbonds1" partners="0" energy_cutoff="-0.25" backbone="1" bb_bb="1" sidechain="0" residue="1" residue_selector="hbonds_to_1"/>

<HbondsToResidue name="hbonds2" partners="0" energy_cutoff="-0.25" backbone="1" bb_bb="1" sidechain="0" residue="2" residue_selector="hbonds_to_2"/>

<HbondsToResidue name="hbonds3" partners="0" energy_cutoff="-0.25" backbone="1" bb_bb="1" sidechain="0" residue="3" residue_selector="hbonds_to_3"/>

<HbondsToResidue name="hbonds4" partners="0" energy_cutoff="-0.25" backbone="1" bb_bb="1" sidechain="0" residue="4" residue_selector="hbonds_to_4"/>

<HbondsToResidue name="hbonds5" partners="0" energy_cutoff="-0.25" backbone="1" bb_bb="1" sidechain="0" residue="5" residue_selector="hbonds_to_5"/>

<HbondsToResidue name="hbonds6" partners="0" energy_cutoff="-0.25" backbone="1" bb_bb="1" sidechain="0" residue="6" residue_selector="hbonds_to_6"/>

<HbondsToResidue name="hbonds7" partners="0" energy_cutoff="-0.25" backbone="1" bb_bb="1" sidechain="0" residue="7" residue_selector="hbonds_to_7"/>

<HbondsToResidue name="hbonds8" partners="0" energy_cutoff="-0.25" backbone="1" bb_bb="1" sidechain="0" residue="8" residue_selector="hbonds_to_8"/>

<HbondsToResidue name="hbonds9" partners="0" energy_cutoff="-0.25" backbone="1" bb_bb="1" sidechain="0" residue="9" residue_selector="hbonds_to_9"/>

<HbondsToResidue name="hbonds10" partners="0" energy_cutoff="-0.25" backbone="1" bb_bb="1" sidechain="0" residue="10" residue_selector="hbonds_to_10"/>

<CombinedValue name="total_hbonds" threshold="-3.0"> #change it to the -1^∗^number of hydrogens bonds required

<Add filter_name="hbonds1" factor="-0.5"/>

<Add filter_name="hbonds2" factor="-0.5"/>

<Add filter_name="hbonds3" factor="-0.5"/>

<Add filter_name="hbonds4" factor="-0.5"/>

<Add filter_name="hbonds5" factor="-0.5"/>

<Add filter_name="hbonds6" factor="-0.5"/>

<Add filter_name="hbonds7" factor="-0.5"/>

<Add filter_name="hbonds8" factor="-0.5"/>

<Add filter_name="hbonds9" factor="-0.5"/>

<Add filter_name="hbonds10" factor="-0.5"/>

</CombinedValue>

#Remove designs with any residue with a hydrogen bond acceptor that makes more than allowed number of h-bonds

<OversaturatedHbondAcceptorFilter name="oversat" scorefxn="SFXN_STD" max_allowed_oversaturated="0" consider_mainchain_only="false" confidence="1" />

#Remove designs with more than 5 N-methylated amino acids

<ResidueCount name="nme_count" max_residue_count="5" include_property="N_METHYLATED" confidence="1" />

<ResidueCount name="nme_count2" max_residue_count="5" include_property="N_METHYLATED" confidence="1" />

<SimpleMetricFilter name="C6s" metric="count_unsats" cutoff="0" comparison_type="lt_or_eq"/>

</FILTERS>

<MOVERS>

#Construct the intial poly glycine chain

<PeptideStubMover name="intial_stub" reset="true">

<Append resname="GLY" />

<Append resname="GLY" />

<Append resname="GLY" />

<Append resname="GLY" />

<Append resname="GLY" />

<Append resname="GLY" />

<Append resname="GLY" />

<Append resname="GLY" />

<Append resname="GLY" />

<Append resname="GLY" />

</PeptideStubMover>

#Declare the peptide bond and cyclization constraints between the N- and C-terminal

<PeptideCyclizeMover name="cyclize_peptide" />

#Set the intial phi/psi/omega torsions for the poly glycine chain

<SetTorsion name="torsion1">

<Torsion residue="ALL" torsion_name="omega" angle="180.0" />

<Torsion residue="1,2,3,4,5,6,7,8,9,10" torsion_name="rama" angle="rama_biased" custom_rama_table="flat_symm_dl_aa_ramatable"/>

</SetTorsion>

#Set some filtering criteria based on internal h-bonds and oversaturation to accept or reject the solution from GenKIC

<ParsedProtocol name="preselection_pp">

<Add filter="total_hbonds"/>

<Add filter="oversat"/>

<Add mover="cyclize_peptide" />

</ParsedProtocol>

#GenKIC method for cyclizing the peptide

<GeneralizedKIC name="genkic1" closure_attempts="1000" selector="lowest_energy_selector" pre_selection_mover="preselection_pp" stop_when_n_solutions_found="1" stop_if_no_solution="500" selector_scorefunction="SFXN_hbond_bb" >

<AddResidue res_index="7" />

<AddResidue res_index="8" />

<AddResidue res_index="9" />

<AddResidue res_index="10" />

<AddResidue res_index="1" />

<AddResidue res_index="2" />

<AddResidue res_index="3" />

<AddResidue res_index="4" />

<AddResidue res_index="5" /> #we are leaving resn 6 out of loop to be closed

<SetPivots atom1="CA" atom2="CA" atom3="CA" res1="7" res2="2" res3="5" />

<CloseBond prioratom_res="10" prioratom="CA" res1="10" atom1="C" res2="1" atom2="N" followingatom="CA" followingatom_res="1" angle1="121.69997" angle2="116.199993" bondlength="1.328685" torsion="180.0" randomize_flanking_torsions="false" />

<AddPerturber effect="set_dihedral">

<AddAtoms atom1="C" res1="10" res2="1" atom2="N" />

<AddValue value="180.0" />

</AddPerturber>

<AddPerturber effect="randomize_alpha_backbone_by_rama" custom_rama_table="flat_symm_dl_aa_ramatable">

<AddResidue index="7" />

<AddResidue index="8" />

<AddResidue index="9"/>

<AddResidue index="10"/>

<AddResidue index="1"/>

<AddResidue index="2"/>

<AddResidue index="3"/>

<AddResidue index="4"/>

<AddResidue index="5"/>

</AddPerturber>

<AddFilter type="loop_bump_check" />

<AddFilter type="rama_prepro_check" residue="7" rama_cutoff_energy="2" />

<AddFilter type="rama_prepro_check" residue="2" rama_cutoff_energy="2" />

<AddFilter type="rama_prepro_check" residue="5" rama_cutoff_energy="2" />

</GeneralizedKIC>

#Add constraints to set min and max number of some amino acids

<AddCompositionConstraintMover name="global" filename="comp/global_preferences.comp" selector="sel_A" />

<AddCompositionConstraintMover name="pro_comp" filename="comp/pro.comp" selector="sel_A" />

#sequence design with upweighted hydrogen bonding score terms to favor internal backbon-to-backbone hydrogen bonds

<FastDesign name="fdesign_hbond_upweighted" scorefxn="SFXN_STD_high_hbond" repeats="1" task_operations="limitchi2,ex1_ex2,ex1,d_res,dpro_res,l_res,lpro_res" ramp_down_constraints="False" >

<MoveMap name="fdesign_step1_mm">

<Chain number="1" chi="1" bb="1"/>

</MoveMap>

</FastDesign>

#Calculate the number of unsats in the peptide

<RunSimpleMetrics name="run_metrics" metrics="count_unsats" prefix="end_unsat" />

#mutate the selected residues to their N-methyl versions

<ModifyVariantType name="N_methylation" add_type="N_METHYLATION" residue_selector="unsat_amines" />

#design amino acid sequence with STD sfxn

<FastDesign name="fdesign" scorefxn="SFXN_STD" repeats="2" task_operations="limitchi2,ex1_ex2,ex1,d_res,l_res" ramp_down_constraints="False" >

<MoveMap name="fdesign_step2_mm">

<Chain number="1" chi="1" bb="1"/>

</MoveMap>

</FastDesign>

#design amino acid sequence with Cartesian sfxn

<FastDesign name="fdesign_cart" scorefxn="SFXN_CART" repeats="1" task_operations="limitchi2,ex1_ex2,ex1,d_res,l_res" ramp_down_constraints="False" cartesian="True" >

<MoveMap name="fdesign_step3_mm">

<Chain number="1" chi="1" bb="1"/>

</MoveMap>

</FastDesign>

</MOVERS>

<PROTOCOLS>

<Add mover="intial_stub" />

<Add mover="torsion1" />

<Add mover="cyclize_peptide" />

<Add mover="genkic1" />

<Add mover="cyclize_peptide" />

<Add mover="global" />

<Add mover="fdesign_hbond_upweighted" />

<Add filter="total_hbonds" />

<Add filter="oversat" />

<Add mover="N_methylation" />

<Add filter="nme_count" />

<Add mover="pro_comp" />

<Add mover="fdesign" />

<Add mover="N_methylation" />

<Add mover="fdesign_cart" />

<Add mover="cyclize_peptide" />

<Add mover="run_metrics" />

</PROTOCOLS>

<OUTPUT scorefxn="SFXN_CART"/>

</ROSETTASCRIPTS>

We used the following command line options to run the Rosettascripts described earlier:

<path_to_rosetta_binary_files>/rosetta_scripts.default.linuxgccrelease -in:path:database <path_to_rosetta>/main/database -parser:protocol permeability_design.xml -in:file:s input.pdb -nstruct 100 -overwrite -beta_nov16 -packer_palette:extra_base_type_file d_packer_palette_hydrophobic.txt -corrections:beta_nov16 true

The example script filters designs to have at least 3 internal hydrogen bonds and less than 5 N-methylated amino acids. These are pretty stringent constraints, so most of the design attempts will fail to return a solution that satisfies all these constraints. For testing purposes, we recommend changing these thresholds to more permissible values. The script above was tested with Rosetta Git Revision 61f38062d297c1a83107a77cd5b33afceeee53a0.

A packer palette file defining the D-amino acids to be used during design is also required to run the protocol described above. Here is an example packer_palette (**d_packer_palette_hydrophobic.txt**). The following packer palette only allows design with D-alanine, D-phenylalanine, D-isoleucine, D-leucine, D-proline, and D-valine. The file can be tuned to include more amino acids (including non-canonical amino acids) or exclude any specific amino acid that must be avoided during the sequence design step.

DALA DPHE DILE DLEU DPRO DVAL

Running the Rosetta Scripts protocol described above also requires a residue file that restricts the allowed amino acids at positions with positive ɸ values to only D-amino acids. Following is an example resfile (**d_res.txt)** that can be used to design with only D-amino acids when combined with a residue selector that selects for positions with positive ɸ values.

NOTAA CDEQNSTYRHKWG

start

Similarly, the design method also requires a resfile file to define amino acids allowed at residue positions with negative phi values. An example resfile (**l_res.txt**) file is given below:

PIKAA AVLFIP

start

We also use amino acids composition constraints to control the distribution and total number of some amino acids. Details about the AA composition constraints and AA composition energy can be found in the help wiki: https://www.rosettacommons.org/docs/latest/scripting_documentation/RosettaScripts/Movers/movers_pages/AddCompositionConstraintMover

An example composition constraint file (**global.comp**) is shown below.

PENALTY_DEFINITION

TYPE PHE DPH

DELTA_START -2

DELTA_END 2

PENALTIES 0 0 0 5 25

ABSOLUTE 2

BEFORE_FUNCTION CONSTANT

AFTER_FUNCTION QUADRATIC

END_PENALTY_DEFINITION

PENALTY_DEFINITION

TYPE ALA DAL

DELTA_START -2

DELTA_END 2

PENALTIES 0 0 0 5 25

ABSOLUTE 2

BEFORE_FUNCTION CONSTANT

AFTER_FUNCTION QUADRATIC

END_PENALTY_DEFINITION

#### Multistate Design of conformation-switching peptides

We used a multistate design method to generate chameleonic macrocycles. Specifically, we implemented a genetic algorithm in PyRosetta-3 that optimizes mutations to obtain two isoenergetic low energy states for the designed amino acid sequences (*see* Data S5 *for scripts and required files*). The starting sequence is used to generate a list of 1000 variants that contain mutations to different hydrophobic residues while maintaining the original chirality and N-methylation patterns. Each mutant sequence is then threaded onto the original backbone conformations and scored using the REF2015 Rosetta energy function (Park et al., 2016). The sequences are filtered to assure that the total energy of the sequence on both backbones is less than 10 kcal/mol and that the difference between the two states is less than 6 kcal/mol. In order to select for sequences that stabilize both conformations while maintaining low energy, sequences are then given a final score equal to (−5×abs(eA − eB))-(eA-eB) where eA and eB are the Rosetta scores of a given sequence threaded onto the first and second conformations, respectively. The best 500 sequences based on this metric are carried on to the next cycle of evaluation, where a point mutant of each sequence is added to the list, and the process is repeated. This algorithm was run for 1,000 generations and then the best sequence was chosen. Structure prediction is performed to ensure both desired conformational states are featured as low-energy minima in the conformational landscape.

#### Conversion of structured peptides to conformation-switching peptides

For some of the crystallographic confirmed designs, we attempted to identify amino acid substitutions that could create secondary isoenergetic minima. We implemented a PyRosetta script that loops through each amino acid position of a given structure and mutates the original residue to other hydrophobic amino acids while maintaining chirality and N-methylation patterns (*see* supplemental information *for scripts and required files*). Each mutated version of the original structure was then energy minimized using the Rosetta FastRelax protocol (Bhardwaj et al., 2016) to ease any strain induced by the mutation. The structure-energy landscape for the full set of mutated sequence-structure pairs was evaluated using Rosetta cycpep_predict application (Bhardwaj et al., 2016; Hosseinzadeh et al., 2017) and designs with isoenergetic alternate states separated by *cis*/*trans* isomerization were selected for experimental characterization.

#### Structure prediction of the designed macrocycles

We used the Rosetta simple_cycpep_predict application as described previously (Bhardwaj et al., 2016; Hosseinzadeh et al., 2017) to evaluate and conformational landscape for the designed amino acid sequences of macrocycles. We ran the structure prediction calculations using the Rosetta@Home platform. For each macrocycle, we generated > 10^4^ energy minimized cyclic conformations of the designed amino acid sequence. For each conformation, we calculated the RMSD to the design model and the energy using the Rosetta REF2015 energy function (Park et al., 2016). The parameters used to run the scripts on Rosetta@Home are equivalent to running the following script on a compute cluster:

/home/gauravb/cycpep_cst_fix_copy/Rosetta/main/source/bin/simple_cycpep_predict.default.linuxgccrelease -in:path:database /home/gauravb/cycpep_cst_fix_copy/Rosetta/main/database @input.flags -nstruct 100

An example flags file (input.flags) is shown below:

-cyclic_peptide:sequence_file [SEQ_TEXT_FILE]

-symmetric_gly_tables true

-score:weights ref2015.wts

-in:file:native [DESIGN_PDB_FILE]

-in:file:fullatom

-cyclic_peptide:genkic_closure_attempts 1000

-cyclic_peptide:genkic_min_solution_count 1

-cyclic_peptide:min_genkic_hbonds 1

-cyclic_peptide:min_final_hbonds 1

-cyclic_peptide:fast_relax_rounds 20

-cyclic_peptide:rama_cutoff 10.0

-ex1

-ex2

-extrachi_cutoff 5

-mute all

-out:file:silent [OUTPUT_FILE_NAME]

-cyclic_peptide:n_methyl_positions [N_METHYLATED_POSITIONS]

In the flags file above, SEQ_TEXT_FILE needs to be replaced with the path for a text file containing the amino acid sequence of the design model, DESIGN_PDB_FILE needs to be replaced with the path for the PDB file of the design model, OUTPUT_FILE_NAME needs to be replaced with the path and filename for the output silent file name, and N_METHYLATED_POSITIONS needs to be replaced with the comma-separated list of amino acid positions that are N-methylated in the design model.

After the completion of the structure prediction runs, we plotted the Energy vs. RMSD to the design model and selected macrocycles that converged to the design model as the unique low-energy states or as one of the alternate isoenergetic states.

#### Peptide synthesis and purification

Macrocyclic peptides were either purchased from Wuxi AppTec at greater than 99% purity or synthesized in-house using the Fmoc-based solid-phase peptide synthesis methods on an automated CEM Liberty Blue peptide synthesizer at a 0.1 mmol scale. Linear peptides were assembled on Cl-TCP(Cl) ProTide resin purchased from CEM, using standard coupling of 2 minutes at 90°C and deprotection for 1 minute at 90°C, unless the Fmoc-amino acid was being coupled to an N-methylated amino acid in which case coupling was performed for 10 minutes at 90°C. We used Oxyma Pure/DIC as the coupling agent and 20% piperidine in DMF for deprotection as per standard protocols by CEM. Linear, protected peptides were released from the resin by repeated washes with 1% TFA in DCM. The wash volumes containing protected peptide were ejected into a round bottom flask containing a 50:50 mixture of acetonitrile and water of greater volume than the volume of the washes. DCM was removed by rotary evaporation and the resulting mixture was lyophilized to dryness. Dry, protected peptides were solubilized in DCM and placed on a stir plate with a stir bar. Two equivalents of PyAOP were added directly to the solution followed by 5 equivalents of DIEA and left to stir overnight at room temperature. DCM was removed by rotary evaporation leaving an oil-like solution in the round bottom flask, which was resuspended in 50:50 acetonitrile:water and purified by reverse-phase high-performance liquid chromatography (RP-HPLC). Peptides were purified on an Agilent Infinity 1260 HPLC using 1% per minute gradient on Agilent ZORBAX SB-C18, 80Å, 5 μm, 9.4 x 250 mm column with a gradient of solvent A: 0.1% TFA in water, and solvent B: 0.1% TFA in acetonitrile. Mass spectrometry was used to confirm the synthesis of the correct product; purified peptides were direct-injected on a Thermo TSQ Quantum Access mass spectrometer. Isobaric peaks matching the expected molecular weight (labeled as p1 and p2) were seen in some cases and were tested separately for permeability and structure. Shifting the cyclization point during resynthesis removed the isobaric peaks for a subset of such cases. For example, D10.1 was resynthesized by CSBio for rodent studies with a different cyclization point and did not show isobaric peaks during purification. Designs, D8.29, D9.21, D9.28, D9.31, D9.40, D10.40, D10.44, and D11.14 were synthesized and tested with norleucine replacing methionine residues to avoid issues with sidechain oxidation.

#### Parallel Artificial Membrane Permeability Assay (PAMPA)

Passive permeability was assayed using standard methods on a Corning® BioCoat™ Pre-coated PAMPA Plate System (Kansy et al., 1998). Starting stock solutions of peptides were prepared by adding 1-2 mg of peptide in 1 ml of DMSO solution. Stock solutions were diluted 20X in Phosphate buffered saline (PBS) buffer to create solutions with 5% DMSO. 300 uL of peptide solution was added to the donor well and 250 uL of 5% DMSO 1X PBS was added to the acceptor well. Donor and acceptor plates were incubated together for 16-20 hours and transferred to 96-well plates at the end of incubation for measuring concentrations of peptide in starting solution, donor wells, and acceptor wells using an RP-HPLC and mass spectrometry on Agilent 6230 LC/TOF. Samples were separated on a 20%/min gradient of Solvent A (water, 0.1% formic acid) and Solvent B (acetonitrile, 0.1% formic acid) ran using Acquity UPLC BEH C18 1.7 μm column. The area under the curve for the peaks matching the peptide mass was calculated, and peptide concentrations were calculated by fitting sample peak areas to a calibration curve of an 8 point two-fold serial dilution series from the starting donor solution. Propranolol and Cyclosporine were used as positive controls during the PAMPA. Apparent permeability (P_app_) were calculated as follows:

$$P_{app}\;=\;-ln\left(1\;-\;C_{A}/C_{e}\right)/A\;*\;\left(1/V_{D}\;+1/V_{A}\right)\;*\;t;\;C_{e}\;=\;\left(C_{D}\;*\;V_{D}\;+\;C_{A}\;*\;V_{A}\right)/\left(V_{D}\;+V_{A}\right);$$

t = incubation time (s),

C_A_ = compound concentration in the acceptor well at t,

C_D_ = compound concentration in the donor well at t,

C_e_ = compound at equilibrium concentration,

V_A_ = acceptor well volume,

V_D_ = donor well volume,

A = filter area.

#### Caco-2 permeability assay

The permeability of designed peptides was evaluated using an accelerated 6-day Caco-2 permeability assay as described previously (Sevin et al., 2013). In brief, cells were seeded into the 12-well Transwell plates with 0.4 μm polyester membrane inserts (Corning, Merck) to give 4.0 × 10^5^ cells/well. The complete assay medium contained Dulbecco’s modified Eagle medium supplemented with 10% (v/v) fetal bovine serum, 1% (v/v) nonessential amino acids, and 0.4 μg/mL puromycin. The medium in plates was renewed every second day. The inserts received 0.75 mL of assay medium in the upper compartment (apical chamber) and 1.5 mL in the bottom compartment (basolateral chamber) individually. After 6-day incubation or until the cells formed monolayers, the medium in the apical and basolateral chamber was replaced with peptide solutions at 50 μM or Hanks' Balanced Salt Solution (HBSS), separately. The plates were incubated for 90 min at 37°C on an orbital shaker (60 rpm). The peptide concentration of solutions in the apical and basolateral compartments was subsequently quantified using LC-MS (Qstar Elite, AB Sciex Australia) to evaluate the transmembrane flux of peptides in the apical-to-basolateral direction. Membrane integrity of the Caco-2 cell monolayer was validated using trans-epithelial electrical resistance (TEER) measurements before and after the assays. Atenolol and quinidine were included as negative and positive controls in all assays.

#### Single-crystal x-ray diffraction of peptides

Based on the availability of macrocycles, we tried to crystallize 139 designed macrocycles (44 6-8mers and 95 6-12mers) using slow evaporation in 50:50 water:ACN or 50:50 water:isopropanol mixtures or vapor diffusion in ethyl acetate and pentane. Crystal diffraction data were collected from a single crystal at the synchrotron (on APS 24ID-C) and at 100 K. Unit cell refinement, and data reduction were performed using XDS and CCP4 suites (Kabsch, 2010, Winn et al., 2001). The structure was identified by direct methods and refined by full-matrix least-squares on F^2^ with anisotropic displacement parameters for the non-H atoms using SHELXL-2016 (Sheldrick, 2015a, Sheldrick, 2015b). Structure analysis was aided by using Coot/Shelxle (Emsley and Cowtan, 2004, Hübschle, 2011). The hydrogen atoms on heavy atoms were calculated in ideal positions with isotropic displacement parameters set to 1.2 × U_eq_ of the attached atoms. Since direct methods can return initial models with mirror handedness in 50% of the cases, we refined the structures with the handedness of the initial model from direct methods, and inverted using ROSETTA flip_chirality mover for alignment and RMSD calculation. Structural coordinates of the refined X-ray structures deposited to the Cambridge Crystallography Data Centre (CCDC) with the following identifiers: 2131411 (D7.6), 2131412 (D7.8), 2131449 (D8.1), 2131417 (D8.2), 2131450 (D8.3.p1), 2131423 (D8.5.p2), 2131424 (D8.6), 2131249 (D8.9), 2131251 (D8.10), 2131252 (D8.12), 2131463 (D8.13), 2131425 (D8.14), 2131426 (D8.15), 2131427 (D8.17), 2131428 (D8.19), 2131253 (D8.25), 2131291 (D8.26), 2131429 (D9.1), 2131430 (D9.8), 2131245 (D9.24), 2131431 (D9.29), 2131432 (D9.30), 2131433 (D10.1), 2131434 (D10.21), 2131435 (D10.22), 2131436 (D10.23), 2131438 (D10.31 IPA), 2131437 (D10.31 ETP), 2131439 (D10.65), 2131440 (D11.1), 2131451 (D11.3), 2131441 (D11.4), 2131442 (D11.25), 2131292 (D8.31), 2131443 (D8.21), 2131293 (D9.16). X-ray structure for D8.3.p2 deposited to Protein data bank with ID: 7UCP. PDB files for all the X-ray structures are also available in the Data S6 (Experimental Structures).

#### NMR sample preparation and assignments

Peptide samples for 2D data collection were prepared by dissolving 5-10 mg of peptide in d_6_-DMSO, CDCl_3_, or mixtures of H_2_O and d_6_-DMSO. For D8.21 an additional sample with 50% d_6_-DMSO and 50% D_2_O, rather than H_2_O, was prepared for additional data collection to remove complications from water peak suppression; peptide solubility was greatly decreased over the solubility in pure organic solvents. NMR data were collected on Bruker Avance II 600 MHz, Avance III 600 MHz, or Avance II 800 MHz spectrometers, equipped with TCI cryoprobes. In all cases data was collected at 293 K and referenced to internal TMS.

Resonance assignments were made using 2D experiments, including heteronuclear ^15^N-^1^H HSQC, ^3^C-^1^H HSQC, HMBC, HSQC-TOCSY (tau_mix_ = 60 ms), as well as homonuclear ^1^H-^1^H COSY, TOCSY (80 ms), ROESY (tau_mix_ = 200 ms, 7.8 kHz), and NOESY (tau_mix_ = 250 ms). Spectra were processed using TopSpin (Bruker) or NMRPipe and visualized in NMRFAM-SPARKY (Delaglio et al., 1995; Lee et al., 2015), allowing for the generation of chemical shift tables in BioMagResBank (BMRB) format from manual assignments.

#### NMR structure determination

Cyclic peptide structures were calculated using CYANA to generate an ensemble of 20 lowest energy conformations (Güntert et al., 1997; Herrmann et al., 2002). Input files were chemical shifts in BMRB format, phi angle restraints, and either 2D NOESY or 2D ROESY peaks lists with peak intensities. NOESY peaks lists were used for samples in DMSO, and ROESY data for samples in CDCl_3_ since NOESY data was weaker. ROESY data was used to identify peaks due to exchange between the conformations, which have the same sign as the diagonal, and these were not included in the peak lists. Phi angle restraints for HN peaks were determined from measured ^3^J_HNHa_ coupling constants from high resolution ^1^H 1D spectra that were converted to phi angles using the Karplus relationship and parameters with error bars of +/- 30 degrees (Vögeli et al., 2007).

Cyana library files were obtained for D-amino acids and N-methylated residues using CYLIB (Yilmaz and Güntert, 2015). Dihedral omega angles were restrained to *cis* or *trans* based on nuclear Overhauser effect **(**NOE) assignments and cyclic peptides were linked using covalent restraints between the N- and C-termini to constrain the peptide bond geometries. For samples with two major conformations, structures were calculated by creating an extended sequence file with the sequences separated by a 12-13 residue non-steric (invisible) linker and performing the restraint-based modeling calculations of both conformers together. This was done to account for all NOE/ROE cross peaks in the 2D spectra corresponding to both conformations. This avoids problems that would be associated with trying to manually separate the peak list for each conformation, a process complicated by resonance overlap, especially in the methyl group region. Exchange peaks were identified by their opposite sign (from NOE cross peaks) in the ROESY spectrum and were removed from NOESY and ROESY peak lists prior to structure calculations. Conformations with symmetry as indicated by chemical shift degeneracy were restrained to C2 symmetry. Chemical shift assignments and NOE completeness were evaluated by PDBStat v5.21.5 (Tejero et al., 2013). NOESY/ROESY peaks were evaluated by reading Cyana assigned peak lists back into Sparky and manually assessed. Structures were validated with PDBStat v5.21.5 (Tejero et al., 2013) and NMR structure validation statistics are given in Table S4. Chemical shifts assignments for ^1^H, ^13^C, and ^15^N resonances are given in Table S3. NMR structures for the chameleonic peptides are available in Protein Data Bank with the following PDB ID and BMRB IDs: 7UBC and 30997 (D9.16 trans-cis d_6_-DMSO), 7UBD and 30998 (D8.31 cis-cis d_6_-DMSO), 8CTO and 31021 (D8.31 cis-trans d_6_-DMSO), 7UBE and 30999 (D8.21 trans-trans d_6_-DMSO), 7UBF and 31000 (D8.21 trans-trans 50:50 d_6_-DMSO / H_2_O), 8CUN and 31022 (D8.21 cis-cis 50:50 d_6_-DMSO / H_2_O), 7UBG and 31001 (D9.16 trans-trans CDCl_3_), 7UZL and 31019 (D9.16 trans-cis CDCl_3_), 7UBH and 31002 (D8.31 cis-cis CDCl_3_), 7UBI and 31003 (D8.21 trans-trans CDCl_3_), 8CWA and 31023 (D8.21 trans-cis CDCl_3_). PDB files for all the NMR structures are also available in the Data S6 (Experimental Structures).

#### NMR amide temperature coefficient studies

Temperature dependence of amide ^1^H chemical shifts were determined by recording 1D NMR variable temperature experiments on a Bruker Avance III 600 MHz spectrometer. Spectra were measured on d_6_-DMSO samples from 293 to 323 K and for CDCl_3_ samples from 288 to 323 K, recorded in 5 K increments and referenced to TMS at 0 ppm. Previous assignments were used and HN amide proton temperature coefficients Δδ(^1^H)/ΔT (ppb/K) were determined from the slope of the linear fit of chemical shift vs. temperature.

#### *In vivo* oral bioavailability assays

We selected four designed macrocycles with diverse structures and *in vitro* rates of permeability for *in vivo* oral bioavailability studies in the rodent models. Due to the intensive resource requirements for such studies, these studies were conducted by three different research groups. D8.3.p1 and D10.1 were studied in female Balb/C mice by van Voorhis group at University of Washington. D11.2 was evaluated in Sprague Dawley (SD) rats by Robert Griffin’s group at Takeda Pharmaceuticals. D11.3 was studied in the male swiss albino mice by the contract research organization, GVK Biosciences Pvt. Ltd., Hyderabad, India.

#### Evaluating pharmacokinetic properties of D8.3.p1 and D10.1

As a preliminary safety screen, peptides were first tested for cytotoxicity *in vitro* against two mammalian cell lines. CRL-8155 human lymphocyte and HepG2 human hepatocyte cells are seeded in 96-well plates and incubated for 48 hours in the presence of test peptide (serial-2 dilutions, from 80 μM to 1.25 μM, in triplicate). At the end of the incubation period, cells were visually assessed before alamarBlue™, a resazurin-based cell viability reagent which measures metabolic activity is added to the plate, and fluorescence was measured on a microplate reader. Fluorescence signals resulting from cell viability changes were compared with control wells to calculate 50% cytotoxic concentration (CC_50_) values.

Pharmacokinetic properties were evaluated in Balb/C female mice (10-12 weeks old, avg. weight 20g, in triplicates) after dosing the peptides through oral gavage (PO), subcutaneous (SQ), and intravenous (IV) routes. Blood samples were collected at multiple time points and plasma was separated from whole blood using centrifugation and stored at -80°C. Drug was extracted from plasma using 80% Acetonitrile (ACN) in water with 0.1% formic acid (FA) and an internal standard. Samples were mixed, centrifuged, and supernatant harvested for LC-MS analysis. For RP-HPLC analysis, peptides were evaluated using Agilent ZORBAX Eclipse Plus C18 1.8 μm, 2.1 mm x 50 mm and Waters Xevo TQ-S micro Triple Quadrupole/ACQUITY UPLC H-Class. A two-component system composed of mobile phase A (0.1% FA in water) and mobile phase B (0.1% FA in 100% ACN) was used at a flow rate of 0.25 mL/min. Peptides were bound to an Agilent ZORBAX C18 column at 40°C for 1 min with 95% mobile phase A, and then were eluted with a linear gradient from 5 to 95% mobile phase B for 4 min. MS was operated in multiple reaction monitoring (MRM) mode via the positive electrospray ionization interface using two transitions: *m/z* 886.6/128 and *m/z* 886.6/156 for D8.3.p1, and *m/z* 1139.92/99.97 for D10.1. Pharmacokinetic parameters were modeled using the Phoenix WinNonlin software package.

#### Experimental Parameters for D8.3.p1

Dosing vehicle: 5% DMSO/5% Tween 80/90% PBS (PO, SQ); 5% DMSO/5% Tween 80/20% PEG 400/70% D5W (IV)

Dose volume: 200 mL (PO, SQ); 50 mL (IV)

Dose: 5 mg/kg, 0.5 mg/mL (PO, SQ); 1 mg/kg, 0.4 mg/mL (IV)

Sampling times: 15, 30, 60, 120, 240, 360, 1440 min (PO, SQ); 5, 15, 30, 60, 120, 240, 360 min (IV)

#### Experimental Parameters for D10.1

Dosing vehicle: 5% DMSO/5% Tween 80/90% PBS (PO, SQ); 5% DMSO/5% Tween 80/20% PEG 400/70% D5W (IV)

Dose volume: 200 mL (PO, SQ); 50 mL (IV)

Dose: 12.2 mg/kg, 1.22 mg/mL (PO, SQ); 2.45 mg/kg, 0.98 mg/mL (IV)

Sampling times: 15, 30, 60, 120, 240, 360, 1440 min (PO, SQ); 5, 15, 30, 60, 120, 240, 360 min (IV)

#### Evaluating pharmacokinetic properties of D11.2

We evaluated the pharmacokinetic properties for D11.2 in male SD rats (n=3). D11.2 was dissolved in 2% DMSO/0.1% Tween80 and dosed by IV, PO, and SQ routes. Plasma samples were collected at multiple time points up to 24 hours. The quantification limit was established to be 2 ng/mL for the assay conditions tested. Pharmacokinetic parameters were modeled using the Phoenix WinNonlin software package.

#### Experimental Parameters for D11.2

Dosing vehicle: DMSO (2%) + 0.1% Tween80(IV, PO, SQ)

Dose: 1 mg/kg (PO, SQ, IV)

Sampling times: 0.083, 0.25, 0.5, 1, 2, 4, 6, 8, 24 h (PO, SQ, IV)

#### Evaluating pharmacokinetic properties of D11.3

Pharmacokinetic properties of D11.3 were evaluated by the contract research organization, GVK Bio in Swiss Albino male mice after dosing the peptides through oral gavage (PO), subcutaneous (SQ), and intravenous (IV) routes. Plasma samples were collected at multiple time points up to 24 hours. Animals were dosed with 27 gauge needle through tail vein for IV, gastric gavage needle for PO, and 27 gauge needle on dorsal side for SQ. Plasma concentrations were determined using LC-MS/MS based quantification. Pharmacokinetic parameters were determined using non-compartmental analysis with Phoenix software version 8.1. All animals were healthy throughout the duration of the study.

#### Experimental Parameters for D11.3

Dosing vehicle: DMSO (10%) + 10% solutol in PBS (90%) (IV, PO, SQ)

Dose volume: 200 mL (PO, SQ); 50 mL (IV)

Dose: 10 mg/kg (PO) 1 mg/kg (IV), 5mg/kg (SQ)

Sampling times: 0.08, 0.25, 0.5, 1, 3, 5, 8, 24 h (IV, PO, SQ)

#### Images and Figures

The hydrogen bonding and structure prediction data was plotted using python scripts using pandas (McKinney, 2010), matplotlib (Hunter, 2007) and seaborn (Waskom, 2021) libraries. Three-dimensional structures were rendered in PyMol. Figures were assembled using Biorender.

### Quantification and Statistical Analysis

Sample sizes for PAMPA, Caco-2, and *in vivo* bioavailability studies are noted in the figure legends. Bar graphs for the PAMPA and Caco-2 assays were plotted in GraphPad Prism using the mean values and the standard deviation of replicate measurements as bar height and error bars, respectively. Pharmacokinetic parameters for *in vivo* studies were modeled using the Phoenix WinNonlin software package.

## Data Availability

• X-ray crystal structures described in the manuscript have been deposited to the Cambridge Crystallography Data Centre (CCDC) and are publicly available as of the date of publication. CCDC IDs are listed in the key resources table. • NMR structures and associated data for the chameleonic peptides have been deposited to Protein Data Bank (PDB) and Biological Magnetic Resonance Bank (BMRB) and are publicly available as of the date of publication. PDB and BMRM IDs are listed in the key resources table. • All the scripts used for the design and validations of peptides were implemented in Rosetta Macromolecular Modeling Suite and PyRosetta. The Rosetta software suite and PyRosetta can be downloaded from https://www.rosettacommons.org/ and https://www.pyrosetta.org/. The scripts used to design macrocycle and example command-line flags are included in the supplemental information files. • Example script files, macrocycle sequences, design models, and permeability values are available in the supplemental information files. • Any additional information required to reanalyze the data reported in this paper is available from the lead contact upon request.

## Supplemental information

Table S1. RMSD between the designed models and X-ray crystal structures of the 7- and 8-amino-acid macrocycles, related to Figure 1

Table S2. RMSD between the designed models and X-ray crystal structures of the 9–11 residue macrocycles, related to Figure 3

Table S3. NMR chemical shift assignments for peptides D9.16, D8.21, and D8.31, related to Figure 4

Table S4. NMR validation tables for designs D9.16, D8.31, and D8.21. RMSD calculated for 20 lowest energy structures out of 100, related to Figure 4

Data S1. Structure prediction plots for all designs, related to Figures 1 and 3

Data S2. Amino acid sequences and computational models, related to Figures 1 and 3

Data S3. PAMPA and Caco-2 data, related to Figure 2

Data S4. Oral bioavailability data, related to Figure 5

Data S5. Example design scripts, related to STAR Methods

Data S6. PDB files for experimental structures, related to STAR Methods
