# Supplementary material for: Accurate de novo design of membrane-traversing macrocycles
Source: Cell. 2022 Sep 15;185(19):3520–3532.e26. doi: 10.1016/j.cell.2022.07.019 (PMC9490236; doi:10.1016/j.cell.2022.07.019)
Supplement: Table S1. RMSD between the designed models and X-ray crystal structures of the 7- and 8-amino-acid macrocycles, related to Figure 1 [file mmc1.pdf]

Cell, Volume *185*

Supplemental information

Accurate *de novo* design of membrane-traversing macrocycles

Gaurav Bhardwaj, Jacob O'Connor, Stephen Rettie, Yen-Hua Huang, Theresa A. Ramelot, Vikram Khipple Mulligan, Gizem Gokce Alpkilic, Jonathan Palmer, Asim K. Bera, Matthew J. Bick, Maddalena Di Piazza, Xinting Li, Parisa Hosseinzadeh, Timothy W. Craven, Roberto Tejero, Anna Lauko, Ryan Choi, Calina Glynn, Linlin Dong, Robert Griffin, Wesley C. van Voorhis, Jose Rodriguez, Lance Stewart, Gaetano T. Montelione, David Craik, and David Baker

Table S1: RMSD between the designed models and X-ray crystal structures of the 7- and 8-amino acid macrocycles. Related to Figure 1.

Design Name	RMSD (CA only) (Å)	RMSD backbone (C+N+CA+O+CN) (Å)	RMSD All Heavy Atoms (Å)
**D7.6**	0.22	0.35	0.9
**D7.8**	0.31	0.5	1.43
**D8.1**	0.2	0.21	0.85
**D8.2**	0.2	0.24	0.86
**D8.3.p1***	0.56	0.57	1.23
**D8.3.p2**	0.92	1.1	1.25
**D8.5.p2**	0.11	0.24	0.55
**D8.6**	1.04	1.35	1.56
**D8.9**	0.7	1.04	1.65
**D8.10**	0.22	0.35	0.96
**D8.12**	0.29	0.48	0.79
**D8.13**	0.63	0.65	1.78
**D8.14**	0.46	0.5	1.17
**D8.15**	0.34	0.42	0.98
**D8.17**	1.25	1.7	3.14
**D8.19**	2.43	2.24	4.13
**D8.25**	0.28	0.95	1.07
**D8.26**	0.57	0.7	1.07

*D8.3 peak 1 matches closely with the alternate low-energy state from structure prediction calculations and the X-ray crystal structure shows cis-trans isomerization around one of the prolines and racemization of residue 1 from chemical synthesis.
