# Supplementary material for: Accurate de novo design of membrane-traversing macrocycles
Source: Cell. 2022 Sep 15;185(19):3520–3532.e26. doi: 10.1016/j.cell.2022.07.019 (PMC9490236; doi:10.1016/j.cell.2022.07.019)
Supplement: Table S2. RMSD between the designed models and X-ray crystal structures of the 9–11 residue macrocycles, related to Figure 3 [file mmc2.pdf]

Table S2: RMSD between the designed models and X-ray crystal structures of the 9-11 residue macrocycles. Related to Figure 3.

Design Name	RMSD (CA only) (Å)	RMSD backbone (C+N+CA+O+CN) (Å)	RMSD All Heavy atoms (Å)
**D9.1**	2.36	2	3.03
**D9.8**	0.39	0.33	0.88
**D9.24**	0.89	1.2	2.1
**D9.29**	0.78	1.11	1.66
**D9.30**	2.54	2.49	4.07
**D9.30_LE0***	0.78	0.78	1.47
**D10.1**	0.27	0.27	0.47
**D10.21**	0.38	0.9	1.19
**D10.22**	0.75	0.82	1.58
**D10.23**	0.38	0.41	1.28
**D10.31**	0.47	0.45	0.84
**D10.65**	1.74	1.67	2.47
**D11.1**	0.43	0.43	1.11
**D11.3**	0.4	0.43	0.93
**D11.4**	0.51	0.54	0.93
**D11.25**	0.63	0.87	1.47

*Structure prediction runs for D9.30 identified conformation LE0 with lower energy than the design model. For more details, please see the methods section on computational design of structured membrane-permeable peptides.
