# Supplementary material for: Accurate de novo design of membrane-traversing macrocycles
Source: Cell. 2022 Sep 15;185(19):3520–3532.e26. doi: 10.1016/j.cell.2022.07.019 (PMC9490236; doi:10.1016/j.cell.2022.07.019)
Supplement: Table S3. NMR chemical shift assignments for peptides D9.16, D8.21, and D8.31, related to Figure 4 [file mmc3.pdf]

Table S3: NMR chemical shift assignments for peptides D9.16, D8.21, and D8.31. Related to Figure 4.

D9.16 (trans-cis) in d_6-DMSO resonance assignments. Proton chemical shifts are given in parentheses.

res.	N / CH3	C	C^{α}	C^{β}	other
P1			56.8 (4.72)	29.6 (2.14, 1.85)	C^{γ}, 25.7 (1.98, 2.14); C^{δ}, 47.5 (3.72, 3.58)
*A2	32.0 (3.09)		49.9 (4.81)	14.1 (1.36)	
A3	117.0 (7.65)		50.3 (4.07)	16.8 (1.27)	
V4	116.7 (6.78)		55.0 (4.20)	29.9 (1.97)	$C^{\gamma1}$, 19.3 (0.88); $C^{\gamma2}$, 18.8 (0.81)
*L5	30.9 (3.01)		54.3 (5.16)	35.7 (1.60, 1.60)	C^{γ}, 24.6 (1.31); $C^{\delta1}$, 21.2 (0.76); $C^{\delta2}$, 23.8 (0.9)
L6	114.1 (6.41)		49.5 (4.48)	41.9 (1.41, 1.48)	C^{γ}, 24.8 (1.60); $C^{\delta1}$, 21.9 (0.91); $C^{\delta2}$, 24.0 (0.9)
L7	120.0 (8.41)		51.7 (3.90)	37.5 (1.32, 1.51)	C^{γ}, 24.6 (1.77); $C^{\delta1}$, 20.0 (0.78); $C^{\delta2}$, 23.7 (0.92)
cP8			60.5 (4.22)	32.1 (2.28, 2.02)	C^{γ}, 22.0 (1.42, 1.78); C^{δ}, 47.0 (3.20, 3.29)
L9	121.2 (7.95)		53.8 (4.82)	37.1 (1.27, 1.95)	C^{γ}, 25.9 (1.34); $C^{\delta1}$, 22.7 (0.74); $C^{\delta2}$, 21.4 (0.86)

D9.16 in $CDCl_3$; conformation 1-9 is trans-Pro 8 (trans-trans) and 21-29 is cis Pro 8 (trans-cis). Pro 1 & 21 are trans.

res.	N / CH3	C	C^{α}	C^{β}	other
tP1		171.9	57.2 (4.57)	28.0 (1.79, 2.23)	C^{γ}, 25.4 (2.01, 2.17); C^{δ}, 47.0 (3.74, 3.62)
*A2		171.1	52.8 (5.38)	13.2 (1.29)	
A3	122.4 (8.41)	174.3	48.0 (4.83)	17.4 (1.32)	
V4	120.8 (6.71)	174.7	57.1 (4.15)	29.8 (2.04)	$C^{\gamma1}$, 18.0 (0.98); $C^{\gamma2}$, 19.9 (1.06)
*L5		171.2	56.0 (5.55)	36.3 (2.04, 1.64)	C^{γ}, 24.9 (1.43); $C^{\delta1}$, 20.9 (0.93); $C^{\delta2}$, 23.7 (0.97);
L6	114.8 (7.46)	173.6	53.9 (4.34)	40.0 (1.97, 1.67)	C^{γ}, 25.3 (1.83); $C^{\delta1}$, 20.6 (0.91); $C^{\delta2}$, 23.4 (0.98)
L7	112.8 (7.20)		48.9 (4.74)	42.9 (1.61, 1.14)	C^{γ}, 24.6 (1.52); $C^{\delta1}$, 21.20 (1.00); $C^{\delta2}$, 23.8 (0.81)
tP8		171.0	58.8 (4.74)	25.3 (2.45, 1.79)	C^{γ}, 25.3 (2.10, 2.01); C^{δ}, 46.9 (3.50, 3.39)
L9	123.1 (8.04)		49.7 (4.72)	40.7 (1.65, 1.51)	C^{γ}, 24.9 (1.73); $C^{\delta1}$, 21.2 (1.00); $C^{\delta2}$, 23.4 (0.95)
P21		174.2	56.9 (4.58)	29.6 (2.14, 2.09)	C^{γ}, 25.7 (2.25, 2.02); C^{δ}, 47.2 (3.81, 3.76)
*A22		171.1	54.2 (5.03)	14.1 (1.50)	
A23	118.6 (7.69)	174.0	49.6 (4.47)	16.3 (1.40)	
V24	120.0 (6.90)	173.6	56.4 (4.16)	29.7 (2.18)	$C^{\gamma1}$, 18.9 (0.90); $C^{\gamma2}$, 19.6 (1.00)
*L25		170.7	55.3 (5.36)	35.8 (1.87, 1.63)	C^{γ}, 24.8 (1.38); $C^{\delta1}$, 20.0 (0.85); $C^{\delta2}$, 23.6 (0.93)
L26	116.3 (6.93)		50.4 (4.58)	41.1 (1.51, 1.87)	C^{γ}, 25.0 (1.70); $C^{\delta1}$, 21.3 (0.96); $C^{\delta2}$, 23.4 (0.98)
L27	* (5.82)		51.8 (4.11)	38.7 (1.69, 1.26)	C^{γ}, 24.7 (1.78); $C^{\delta1}$, 20.4 (0.86); $C^{\delta2}$, 23.4 (0.98)
cP28		170.4	60.7 (4.28)	31.7 (2.60, 2.00)	C^{γ}, 22.0 (1.88, 1.67); C^{δ}, 46.9 (3.51, 3.38)
L29	123.7 (8.15)	172.5	50.5 (4.92)	36.9 (1.93, 1.49)	C^{γ}, 26.0 (1.38); $C^{\delta1}$, 20.9 (0.93); $C^{\delta2}$, 22.5 (0.82)

D8.31 in $CDCl_3$ resonance assignments for conformation with symmetric cis-cis N-methyl Leu 3 and 7.

res.	NH / CH3	C	C^{α}	C^{β}	other
dA1	118.1 (7.80)	171.3	45.9 (5.01)	17.4 (1.27)	
dP2		173.4	55.8 (4.60)	29.8 (2.08, 2.01)	C^{γ}, 25.3 (2.36, 1.92); C^{δ}, 47.9 (3.77, 3.63)
*dL3	30.0 (2.82)	169.8	59.7 (4.54)	36.9 (2.04, 1.69)	C^{γ}, 24.4 (1.45); $C^{\delta1}$, 23.4 (0.98); $C^{\delta2}$, 21.7 (0.93)
dV4	113.3 (7.48)	171.2	60.3 (4.39)	30.8 (2.14)	$C^{\gamma1}$, 18.1 (0.93); $C^{\gamma2}$, 19.5 (0.97)

D8.31 in d_6-DMSO; conformation 1-4 is symmetric cis-cis and 21-28 is cis-trans.

res.	N / CH3	C	C^{α}	C^{β}	other
dA1	116.2 (7.57)	170.5	46.1 (4.75)	17.5 (1.15)	
dP2			56.0 (4.65)	29.6 (2.08, 1.80)	C^{γ}, 25.2 (2.16, 1.79); C^{δ}, 47.5 (3.61, 3.43)
c-*dL3	29.7 (2.66)	167.0	59.2 (4.55)	36.8 (1.83, 1.64)	C^{γ}, 24.5 (1.40); $C^{\delta1}$, 23.7 (0.91); $C^{\delta2}$, 22.4 (0.89)
dV4	114.1 (7.63)	170.6	60.6 (4.03)	30.4 (2.00)	$C^{\gamma1}$, 19.7 (0.88); $C^{\gamma2}$, 18.9 (0.85)
dA21	114.1 (7.36)		47.6 (4.60)	17.0 (1.17)	
dP22			56.5 (4.82)	29.2 (2.06, 1.73)	C^{γ}, 25.3 (2.13, 1.89); C^{δ}, 47.3 (3.59, 3.45)
c-*L23	29.3 (2.60)	169.8	59.0 (4.70)	36.3 (1.81, 1.63)	C^{γ}, 24.4 (1.32); $C^{\delta1}$, 24.0 (0.91); $C^{\delta2}$, 22.5 (0.89)
dV24	115.6 (8.43)		61.0 (4.01)	30.6 (2.03)	$C^{\gamma1}$, 19.6 (0.85); $C^{\gamma2}$, 19.2 (0.86)
dA25	117.3 (7.35)		46.1 (4.76)	18.2 (1.13)	
dP26			57.6 (4.61)	29.12 (2.22, 1.76)	C^{γ}, 25.3 (2.06, 1.89); C^{δ}, 48.1(3.67, 3.67);
t-*dL27	40.9 (3.22)		68.4 (3.70)	38.3 (2.22, 1.66)	C^{γ}, 25.3 (1.42); $C^{\delta1}$, 23.5 (0.87); $C^{\delta2}$, 22.1 (0.88)
dV28	112.3 (7.96)	170.8	60.2 (4.01)	31.3 (1.84)	$C^{\gamma1}$, 19.5 (0.84); $C^{\gamma2}$, 18.4 (0.77)

D8.21 in d_6-DMSO resonance assignments for symmetric trans-trans conformation for D-Pro 3 & 7

res.	N / CH3	C	C^{α}	C^{β}	other
dV1	121.0 (8.57)	172.0	54.8 (4.34)	30.3 (2.00)	$C^{\gamma1}$, 19.2 (0.86); $C^{\gamma2}$, 19.1 (0.89)
*L2	30.9 (3.01)	170.8	52.6 (5.35)	37.7 (1.52, 1.52)	C^{γ}, 24.3 (1.39); $C^{\delta1}$, 23.9 (0.90); $C^{\delta2}$, 22.5 (0.87)
dP3		170.6	61.0 (4.31)	28.4 (2.01, 1.74)	C^{γ}, 25.2 (1.69, 1.69); C^{δ}, 47.3 (3.48, 3.02)
L4	113.4 (7.04)	172.2	50.5 (4.54)	43.4 (1.36, 1.36)	C^{γ}, 24.3 (1.58); $C^{\delta1}$, 23.6 (0.83); $C^{\delta2}$, 22.5 (0.87)

D8.21 in 50:50 d_6-DMSO/water resonance assignments. 1-4 are trans-trans and 21-24 are cis-cis conformation

res.	N / CH3	C	C^{α}	C^{β}	other
dV1	116.7 (8.02)	173.6	55.4 (4.64)	31.0 (2.07)	$C^{\gamma1}$, 19.1 (0.9); $C^{\gamma2}$, 17.4 (0.88)
*L2	31.3 (3.09)		53.6 (5.13)	36.1 (1.79, 1.49)	C^{γ}, 24.7 (1.50); $C^{\delta1}$, 23.2 (0.95); $C^{\delta2}$, 21.4 (0.89)
dP3			60.9 (4.40)	29.5 (2.23, 1.92)	C^{γ}, 24.5 (1.97, 1.9); C^{δ}, 47.8 (3.71, 3.65)
L4	116.83 (7.30)		51.7 (4.51)	41.3 (1.36, 1.36)	C^{γ}, 24.8 (1.46); $C^{\delta1}$, 23.1 (0.82); $C^{\delta2}$, 21.1 (0.82)
dV21	120.7 (8.76)	171.5	54.5 (4.89)	31.5 (2.06)	$C^{\gamma1}$, 18.7 (0.85); $C^{\gamma2}$, 18.7 (0.81)
*L22	30.6 (2.94)		52.6 (5.29)	38.0 (1.80, 1.25)	C^{γ}, 24.5 (1.37); $C^{\delta1}$, 22.8 (0.84); $C^{\delta2}$, 22.7 (0.84)
c-dP23			59.6 (4.20)	33.2 (2.31, 2.03)	C^{γ}, 22.2 (1.86, 1.64); C^{δ}, 48.2 (3.53, 3.44)
L24	116.2 (8.33)		51.1 (4.59)	41.6 (1.84, 1.48)	C^{γ}, 24.0 (1.46); $C^{\delta1}$, 23.6 (0.92); $C^{\delta2}$, 20.7 (0.87)

D8.21 in $CDCl_3$ resonance assignments; 1-4 are trans-trans and 21-28 are cis-trans

res.	N	C	C^{α}	C^{β}	other
dV1	114.4 (6.69)	171.0	54.2 (4.72)	31.6 (2.01)	$C^{\gamma1}$, 17.5 (0.89); $C^{\gamma2}$, 19.6 (0.97)
*L2	31.0 (3.13)	172.6	52.7 (5.31)	37.5 (1.70, 1.64)	C^{γ}, 24.8 (1.46); $C^{\delta1}$, 22.3 (0.93); $C^{\delta2}$, 23.2 (0.97)
dP3		170.9	60.0 (4.64)	27.5 (1.95, 2.36)	C^{γ}, 24.7 (2.03, 1.94); C^{δ}, 47.3 (3.54, 3.63)
L4	118.7 (7.14)	171.7	52.0 (4.38)	40.7 (1.50, 1.50)	C^{γ}, 24.5 (1.61); $C^{\delta1}$, 21.7 (0.90); $C^{\delta2}$, 23.0 (0.91)
dV21	117.1 (7.37)	168.1	55.8 (4.28)	29.3 (2.30)	$C^{\gamma1}$, 18.5 (1.00); $C^{\gamma2}$, 19.6 (0.97)
*L22	30.6 (3.00)	172.0	53.4 (5.25)	38.3 (1.14, 2.00)	C^{γ}, 24.7 (1.51); $C^{\delta1}$, 22.4 (0.87); $C^{\delta2}$, 23.2 (0.88)
dP23		171.9	58.7 (4.30)	33.7 (1.95, 2.4)	C^{γ}, 22.2 (1.77, 1.86); C^{δ}, 47.8 (3.54, 3.73)
L24	112.4 (5.00)	172.3	50.9 (4.87)	42.6 (1.37, 2.08)	C^{γ}, 25.1 (1.47); $C^{\delta1}$, 21.0 (0.93); $C^{\delta2}$, 24.3 (0.92)
dV25	116.8 (8.65)	176.7	56.7 (4.87)	31.5 (2.18)	$C^{\gamma1}$, 19.4 (0.89); $C^{\gamma2}$, 19.0 (0.80)
*L26	32.2 (3.36)		55.4 (4.50)	36.7 (1.98, 1.39)	C^{γ}, 25.6 (1.62); $C^{\delta1}$, 21.2 (0.96); $C^{\delta2}$, 23.5 (1.04)
dP27		171.3	60.1 (4.71)	27.7 (1.96, 2.57)	C^{γ}, 24.2 (1.96, 2.09); C^{δ}, 46.8 (3.48, 4.00)
L28	117.6 (7.41)	174.0	52.2 (4.61)	43.1 (1.58, 1.18)	C^{γ}, 24.8 (1.72); $C^{\delta1}$, 21.0 (0.93); $C^{\delta2}$, * (0.94)
