# Supplementary material for: Accurate de novo design of membrane-traversing macrocycles
Source: Cell. 2022 Sep 15;185(19):3520–3532.e26. doi: 10.1016/j.cell.2022.07.019 (PMC9490236; doi:10.1016/j.cell.2022.07.019)
Supplement: Table S4. NMR validation tables for designs D9.16, D8.31, and D8.21. RMSD calculated for 20 lowest energy structures out of 100, related to Figure 4 [file mmc4.pdf]

Table S4. NMR validation tables for designs D9.16, D8.31, and D8.21. RMSD calculated for 20 lowest energy structures out of 100. Related to Figure 4.

<table>
<tr><th></th><th>D9.16
DMSO
trans-cis</th><th>D9.16
CDCl3
trans-cis</th><th>D9.16
CDCl3
trans-trans</th><th>D8.31
CDCl3
cis-cis</th><th>D8.31
DMSO
cis-cis</th><th>D8.31
DMSO
cis-trans</th></tr>
<tr><td><u>NMR restraints</u></td><td></td><td></td><td></td><td></td><td></td><td></td></tr>
<tr><td>Total NOEs</td><td>106</td><td>89</td><td>99</td><td>111</td><td>84</td><td>82</td></tr>
<tr><td>Intra-residue</td><td>38</td><td>35</td><td>41</td><td>38</td><td>26</td><td>24</td></tr>
<tr><td>Sequential |i-j|=1</td><td>48</td><td>31</td><td>38</td><td>59</td><td>48</td><td>47</td></tr>
<tr><td>Medium 1<|i-j|<5</td><td>20</td><td>23</td><td>20</td><td>14</td><td>10</td><td>11</td></tr>
<tr><td>Long |i-j|>=5</td><td>0</td><td>0</td><td>0</td><td>73</td><td>0</td><td>0</td></tr>
<tr><td>Hydrogen bonds</td><td>0</td><td>0</td><td>0</td><td>0</td><td>0</td><td>0</td></tr>
<tr><td>PHI dihedral restraints</td><td>5</td><td>5</td><td>5</td><td>4</td><td>4</td><td>4</td></tr>
<tr><td><u>Structure Statistics</u></td><td></td><td></td><td></td><td></td><td></td><td></td></tr>
<tr><td>Violations (mean and s.d.)</td><td></td><td></td><td></td><td></td><td></td><td></td></tr>
<tr><td>Max. distance restraint (Å)</td><td>0.10</td><td>0.14</td><td>0.41</td><td>0.35</td><td>0.07</td><td>0.74</td></tr>
<tr><td>Ave. distance restraints viol. (Å)</td><td>0.02 ± 0.02</td><td>0.03 ± 0.03</td><td>0.07 ± 0.10</td><td>0.19 ± 0.15</td><td>0.02 ± 0.02</td><td>0.09 ± 0.15</td></tr>
<tr><td>Max. dihedral angle (º)</td><td>2.53</td><td>2.45</td><td>1.18</td><td>1.26</td><td>0.30</td><td>3.68</td></tr>
<tr><td>Ave. dihedral viol (º)</td><td>0.86 ± 0.73</td><td>0.78 ± 0.67</td><td>0.36 ± 0.26</td><td>1.18 ± 0.06</td><td>0.19 ± 0.07</td><td>1.27 ± 1.19</td></tr>
<tr><td><u>Average pairwise RMSD</u></td><td></td><td></td><td></td><td></td><td></td><td></td></tr>
<tr><td>backbone (Å)</td><td>0.21 ± 0.07</td><td>0.24 ± 0.09</td><td>0.20 ± 0.05</td><td>0.21 ± 0.10</td><td>0.14 ± 0.12</td><td>0.48 ± 0.09</td></tr>
<tr><td>heavy (Å)</td><td>0.53 ± 0.08</td><td>0.68 ± 0.12</td><td>0.73 ± 0.07</td><td>0.52 ± 0.22</td><td>0.54 ± 0.16</td><td>1.15 ± 0.16</td></tr>
</table>

<table>
<tr><th></th><th>D8.21
DMSO
trans-trans</th><th>D8.21
DMSO50
trans-trans</th><th>D8.21
DMSO50
cis-cis</th><th>D8.21
CDCl3
trans-trans</th><th>D8.21
CDCl3
trans-cis</th></tr>
<tr><td><u>NMR restraints</u></td><td></td><td></td><td></td><td></td><td></td></tr>
<tr><td>Total NOEs</td><td>84</td><td>94</td><td>66</td><td>144</td><td>94</td></tr>
<tr><td>Intra-residue</td><td>22</td><td>38</td><td>24</td><td>48</td><td>30</td></tr>
<tr><td>Sequential |i-j|=1</td><td>48</td><td>36</td><td>32</td><td>56</td><td>38</td></tr>
<tr><td>Medium 1<|i-j|<5</td><td>14</td><td>20</td><td>10</td><td>40</td><td>26</td></tr>
<tr><td>Long |i-j|>=5</td><td>0</td><td>0</td><td>0</td><td>0</td><td>0</td></tr>
<tr><td>Hydrogen bonds</td><td>0</td><td>0</td><td>0</td><td>0</td><td>0</td></tr>
<tr><td>PHI dihedral restraints</td><td>4</td><td>4</td><td>4</td><td>4</td><td>4</td></tr>
<tr><td><u>Structure Statistics</u></td><td></td><td></td><td></td><td></td><td></td></tr>
<tr><td>Violations (mean and s.d.)</td><td></td><td></td><td></td><td></td><td></td></tr>
<tr><td>Max. distance restraint (Å)</td><td>0.19</td><td>0.45</td><td>0.01</td><td>0.30</td><td>0.57</td></tr>
<tr><td>Ave. distance restraints viol. (Å)</td><td>0.06 ± 0.06</td><td>0.08 + 0.08</td><td>0.01 ± 0.00</td><td>0.08 ± 0.08</td><td>0.12 ± 0.16</td></tr>
<tr><td>Max. dihedral angle (º)</td><td>2.23</td><td>2.80</td><td>1.23</td><td>3.12</td><td>4.25</td></tr>
<tr><td>Ave. dihedral viol (º)</td><td>0.46 ± 0.59</td><td>2.17 ± 0.52</td><td>0.35 ± 0.47</td><td>2.56± 0.34</td><td>1.53 ±1.37</td></tr>
<tr><td><u>Average pairwise RMSD</u></td><td></td><td></td><td></td><td></td><td></td></tr>
<tr><td>backbone (Å)</td><td>0.39 ± 0.18</td><td>0.25 ± 0.19</td><td>0.13 ± 0.03</td><td>0.21 ± 0.13</td><td>0.30 ± 0.11</td></tr>
<tr><td>heavy (Å)</td><td>1.02 ± 0.25</td><td>0.74 ± 0.23</td><td>0.68 ± 0.13</td><td>0.64 ± 0.28</td><td>0.76 ± 0.17</td></tr>
</table>
