# Supplementary material for: Accurate de novo design of membrane-traversing macrocycles
Source: Cell. 2022 Sep 15;185(19):3520–3532.e26. doi: 10.1016/j.cell.2022.07.019 (PMC9490236; doi:10.1016/j.cell.2022.07.019)

Accurate *de novo* design of membrane-traversing macrocycles

Supplemental data file 1: Structure prediction plots for all designs.
Related to Figures 1 and 3.

Figure D1.1: Structure-energy landscapes and hydrogen-bonding patterns for designed and selected 6-amino acid macrocycles: Left panels show the torsion bin cluster and predicted conformational landscape for each design. * denotes N-methylated amino acids. Orange points denote an independently predicted structure with no cis-peptide bonds. Grey points represent predicted structures with at least one cis-peptide bond. Blue dots represent the local minimization of the designed macrocycle structure. For hydrogen bonding graphs, the orange boxes highlight the designed intramolecular hydrogen bonds. Amino acids without a backbone hydrogen bond donor (proline, D-proline, N-methylated amino acids) are marked by darker grey columns. Related to Figure 1.

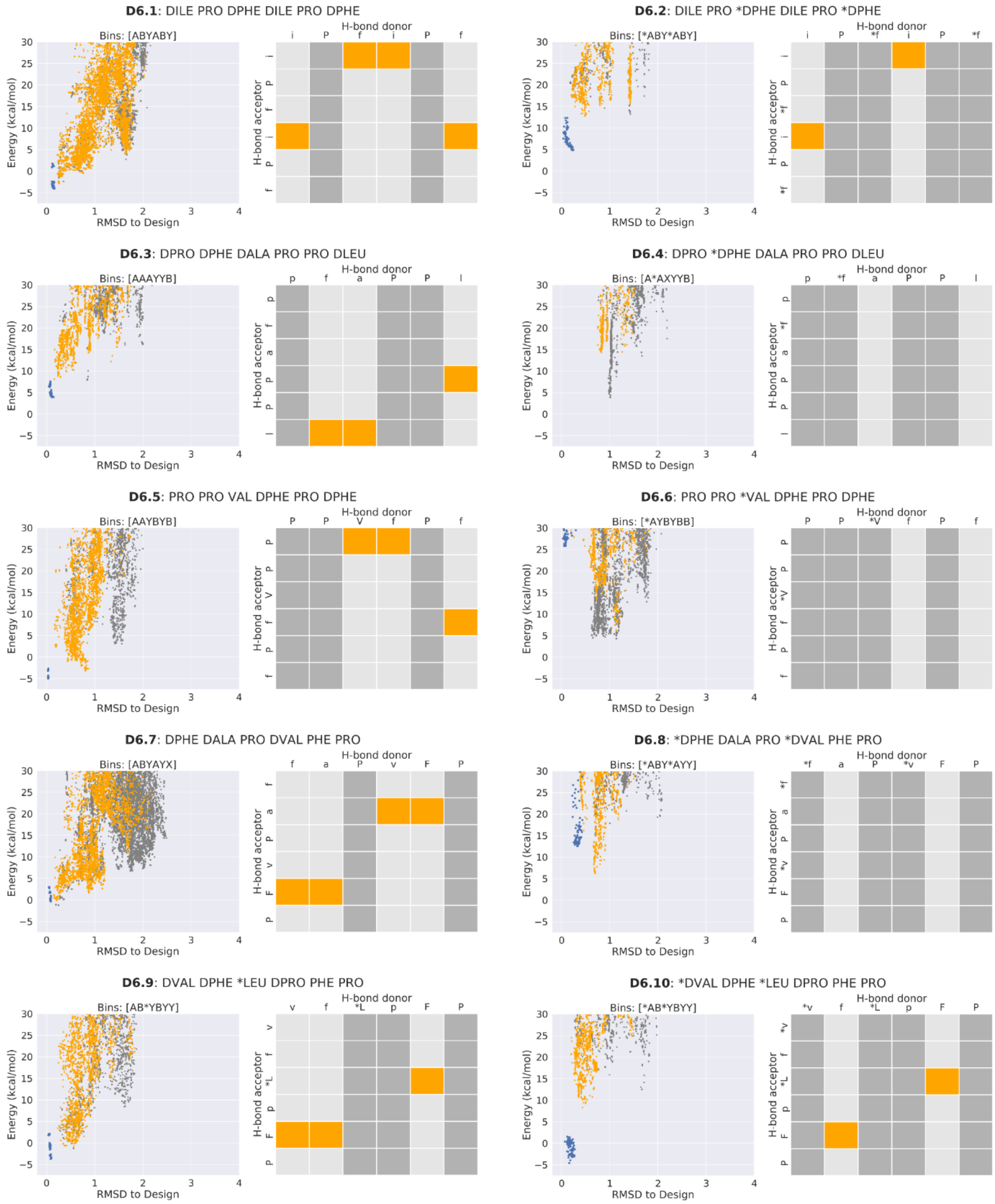

Figure D1.2: Structure-energy landscapes and hydrogen-bonding patterns for designed and selected 7-amino acid macrocycles: Left panels show the torsion bin cluster and predicted conformational landscape for each design. * denotes N-methylated amino acids. Orange points denote an independently predicted structure with no cis-peptide bonds. Grey points represent predicted structures with at least one cis-peptide bond. Blue dots represent the local minimization of the designed macrocycle structure. For hydrogen bonding graphs, the orange boxes highlight the designed intramolecular hydrogen bonds. Amino acids without a backbone hydrogen bond donor (proline, D-proline, N-methylated amino acids) are marked by darker grey columns. Related to Figure 1.

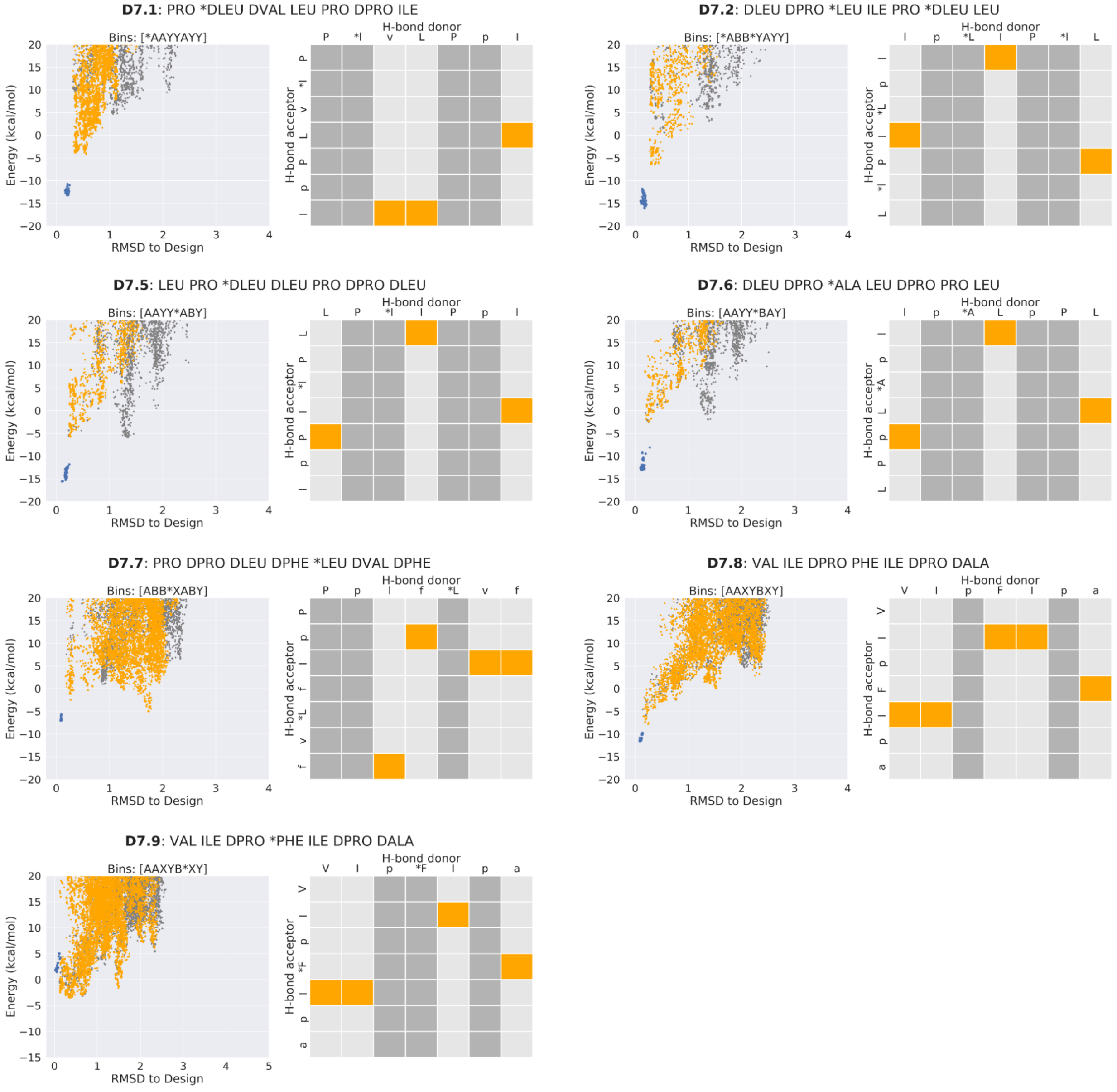

Figure D1.3: Structure-energy landscapes and hydrogen-bonding patterns for designed and selected 8-amino acid macrocycles: Left panels show the torsion bin cluster and predicted conformational landscape for each design. * denotes N-methylated amino acids. Orange points denote an independently predicted structure with no cis-peptide bonds. Grey points represent predicted structures with at least one cis-peptide bond. Blue dots represent the local minimization of the designed macrocycle structure. For hydrogen bonding graphs, the orange boxes highlight the designed intramolecular hydrogen bonds. Amino acids without a backbone hydrogen bond donor (proline, D-proline, N-methylated amino acids) are marked by darker grey columns. Related to Figure 1.

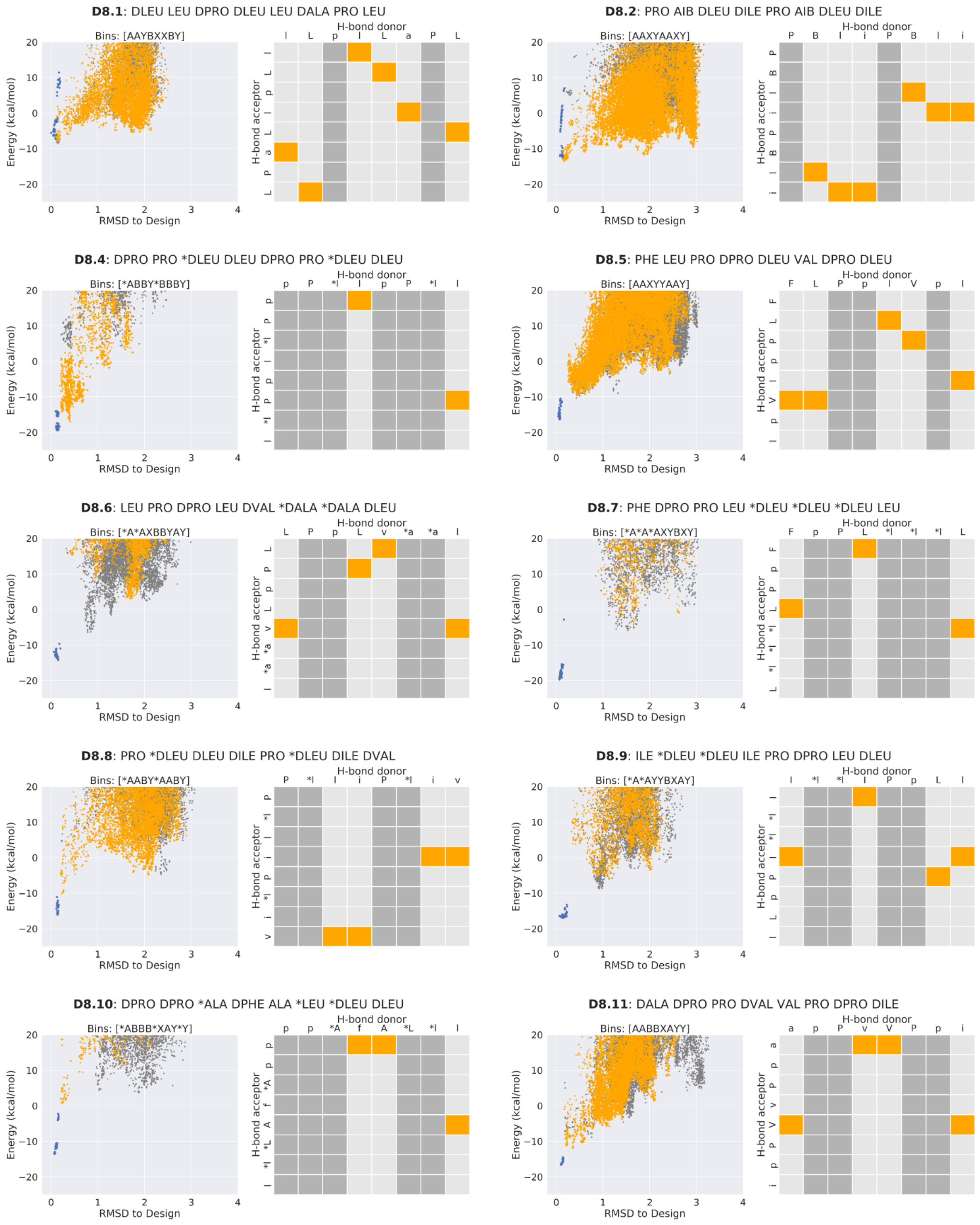

Figure D1.3 (cont.): Structure-energy landscapes and hydrogen-bonding patterns for designed and selected 8-amino acid macrocycles *(full figure legend described earlier).* Related to Figure 1.

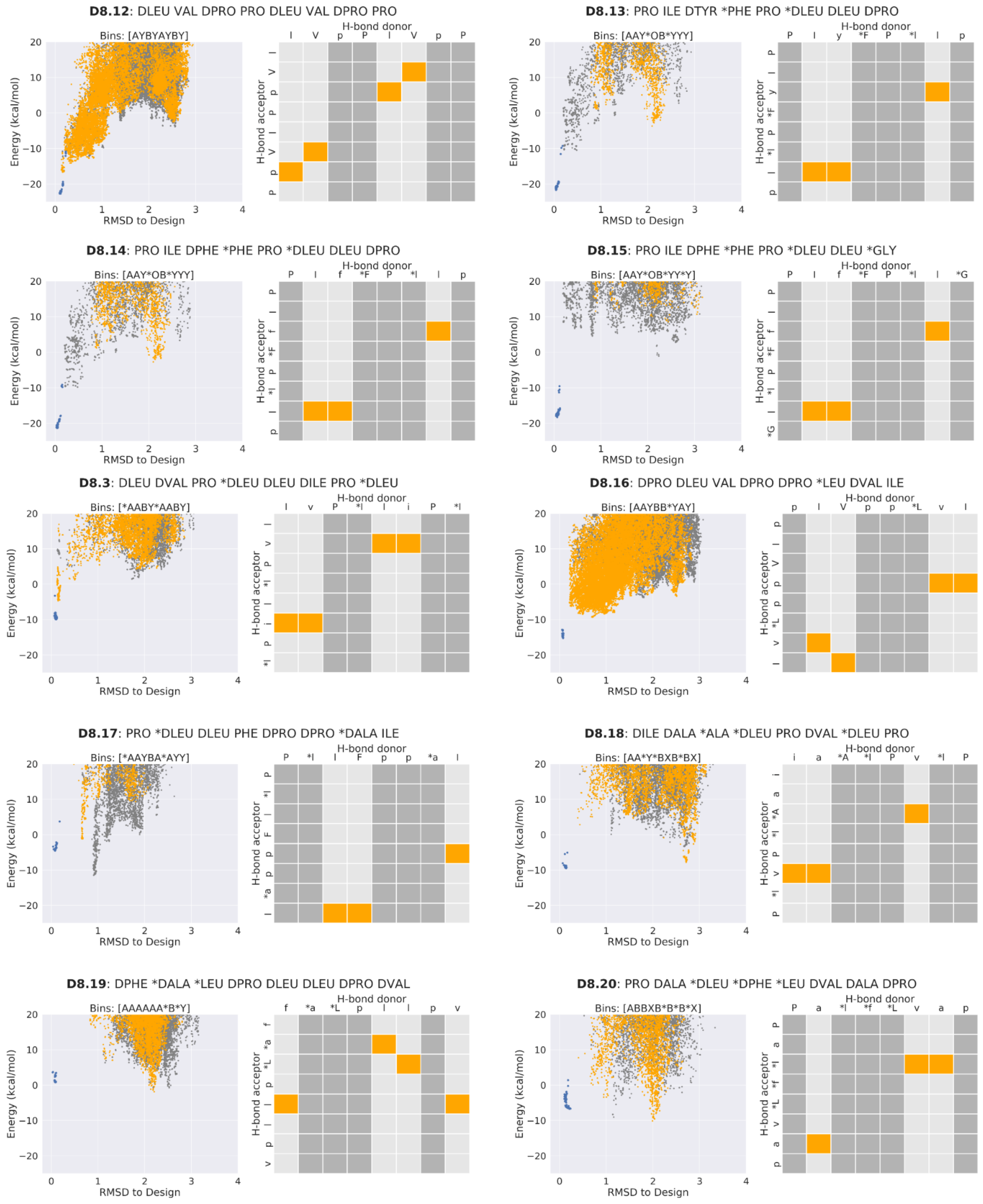

Figure D1.3 (cont.): Structure-energy landscapes and hydrogen-bonding patterns for designed and selected 8-amino acid macrocycles. *(full figure legend described earlier).* Related to Figure 1.

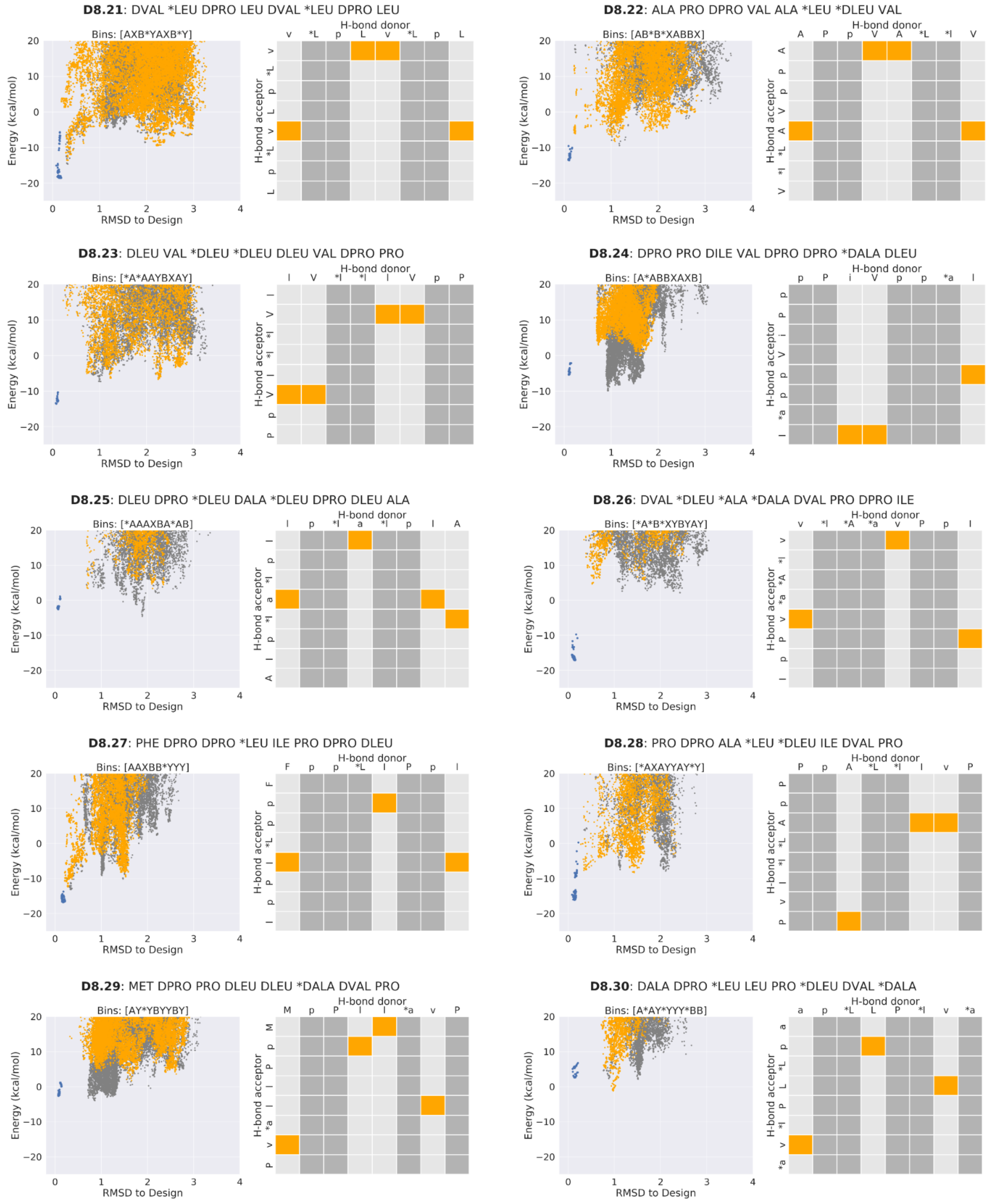

Figure D1.3 (cont.): Structure-energy landscapes and hydrogen-bonding patterns for designed and selected 8-amino acid macrocycles. *(full figure legend described earlier).* Related to Figure 1.

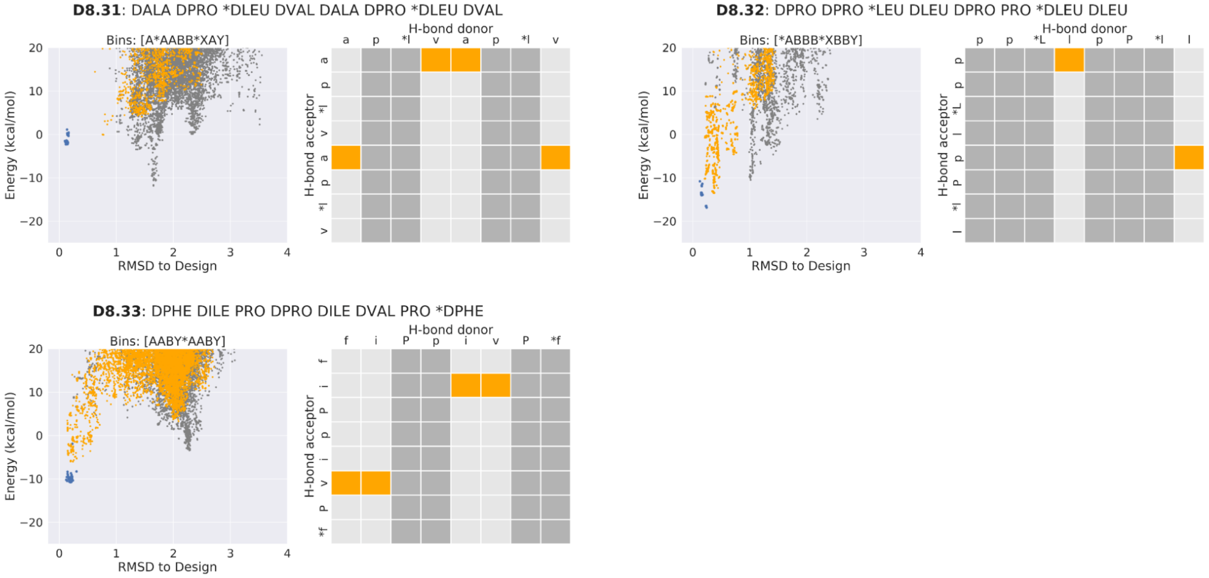

Figure D1.4: Structure-energy landscapes and hydrogen-bonding patterns for designed and selected 9-amino acid macrocycles: Left panels show the torsion bin cluster and predicted conformational landscape for each design. * denotes N-methylated amino acids. Orange points denote an independently predicted structure with no cis-peptide bonds. Grey points represent predicted structures with at least one cis-peptide bond. Blue dots represent the local minimization of the designed macrocycle structure. For hydrogen bonding graphs, the orange boxes highlight the designed intramolecular hydrogen bonds. Amino acids without a backbone hydrogen bond donor (proline, D-proline, N-methylated amino acids) are marked by darker grey columns. Related to Figure 3.

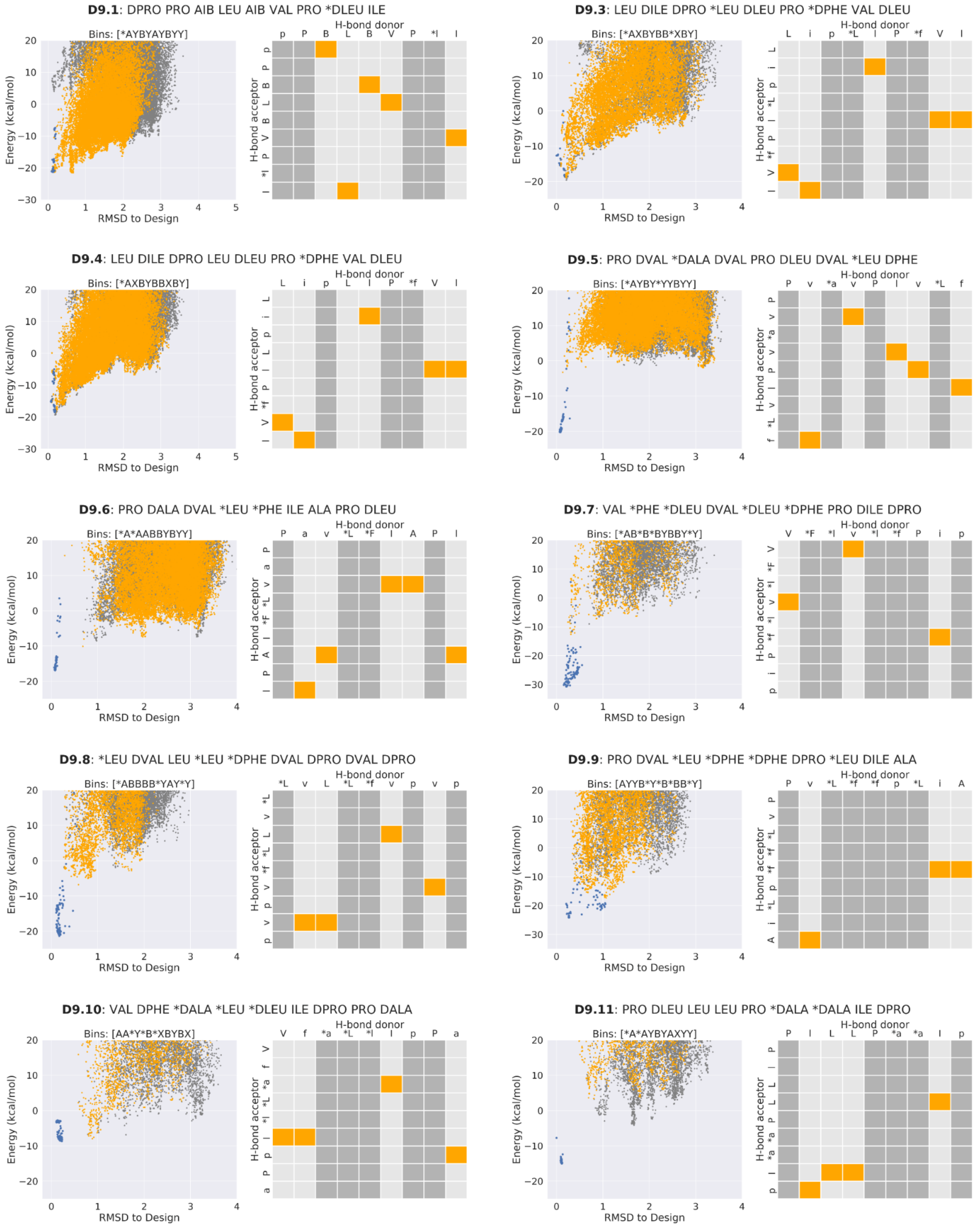

Figure D1.4 (cont.): Structure-energy landscapes and hydrogen-bonding patterns for designed and selected 9-amino acid macrocycles. *(full figure legend described earlier).* Related to Figure 3.

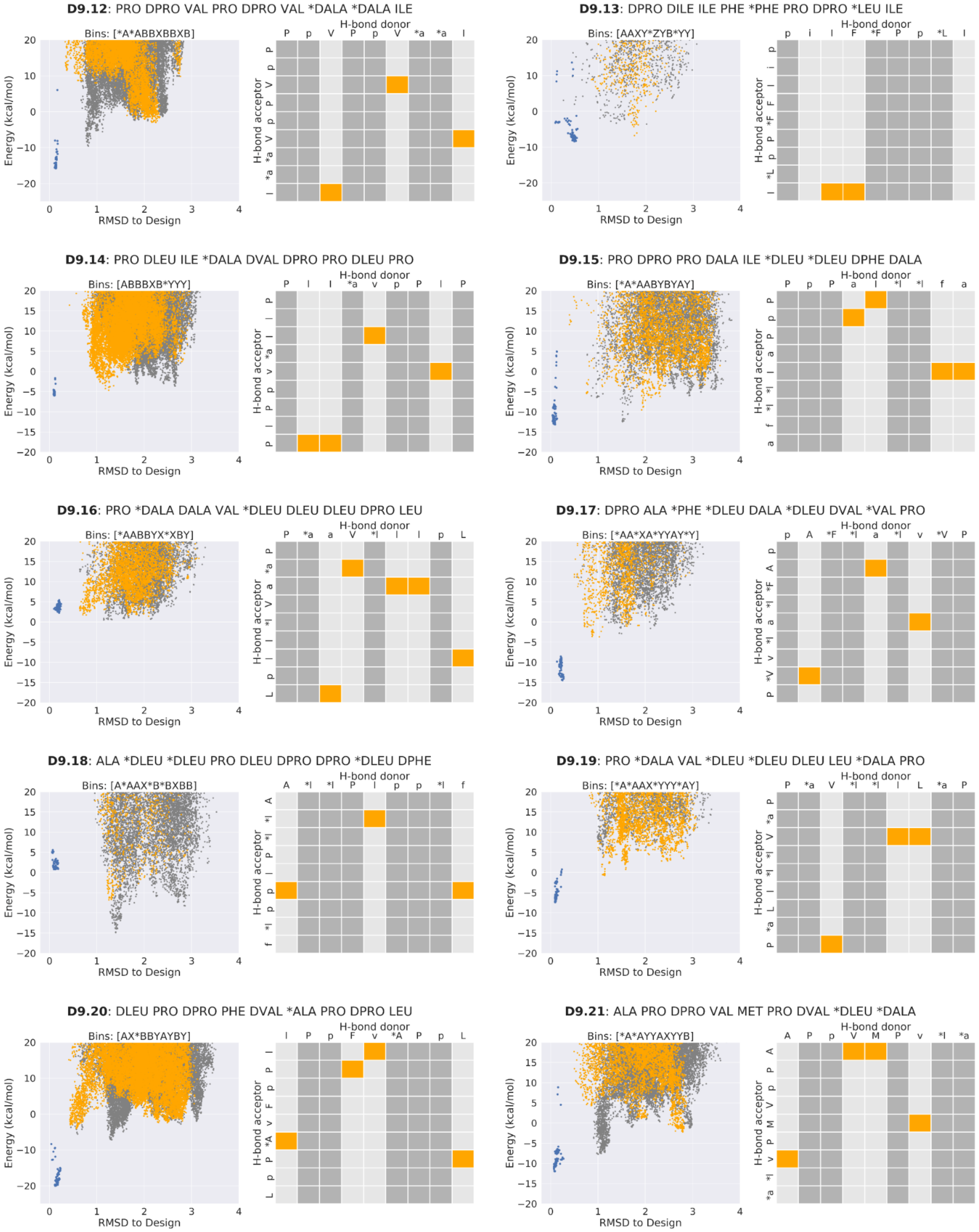

Figure D1.4 (cont.): Structure-energy landscapes and hydrogen-bonding patterns for designed and selected 9-amino acid macrocycles. *(full figure legend described earlier).* Related to Figure 3.

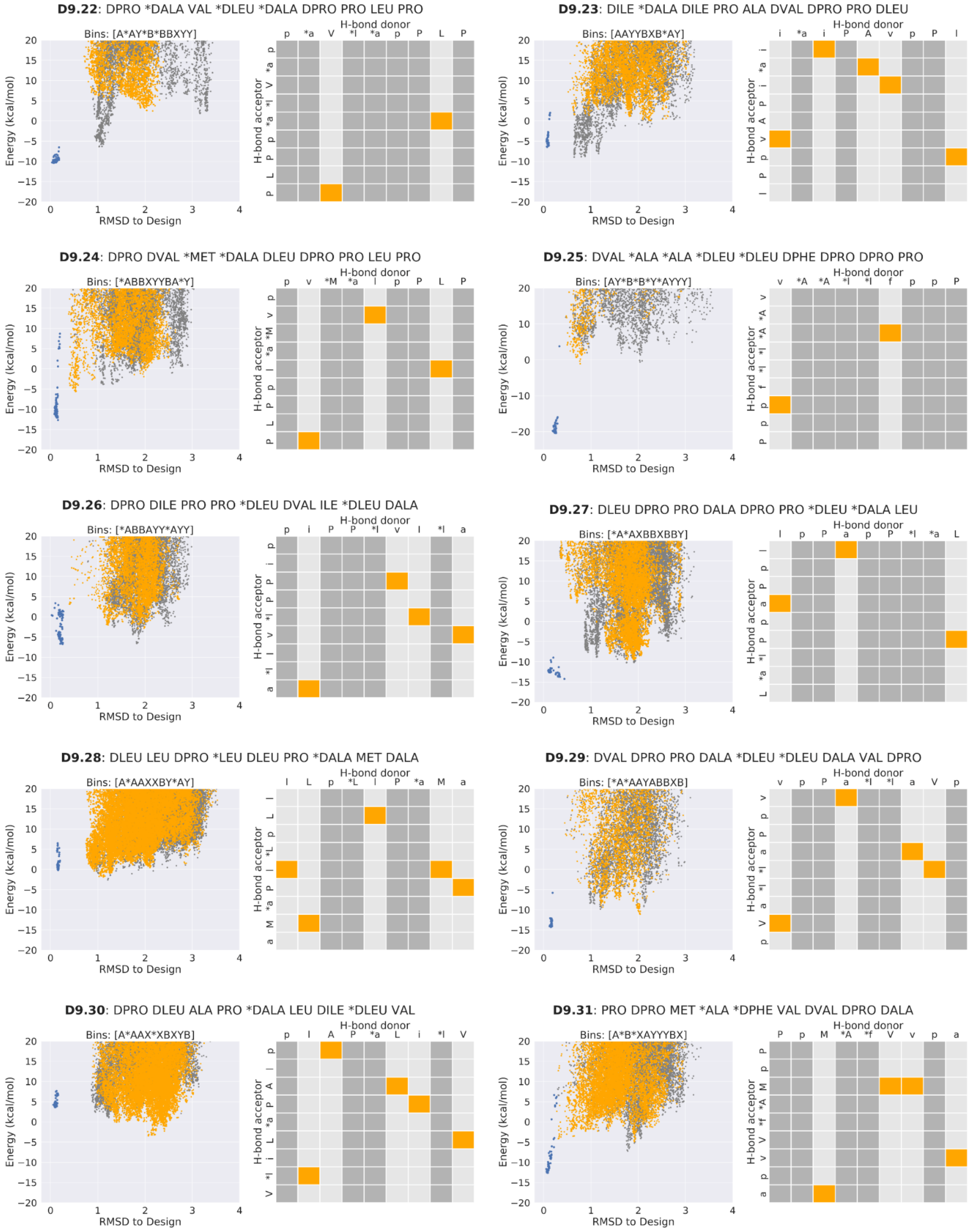

Figure 1.4 (cont.): Structure-energy landscapes and hydrogen-bonding patterns for designed and selected 9-amino acid macrocycles. *(full figure legend described earlier).* Related to Figure 3.

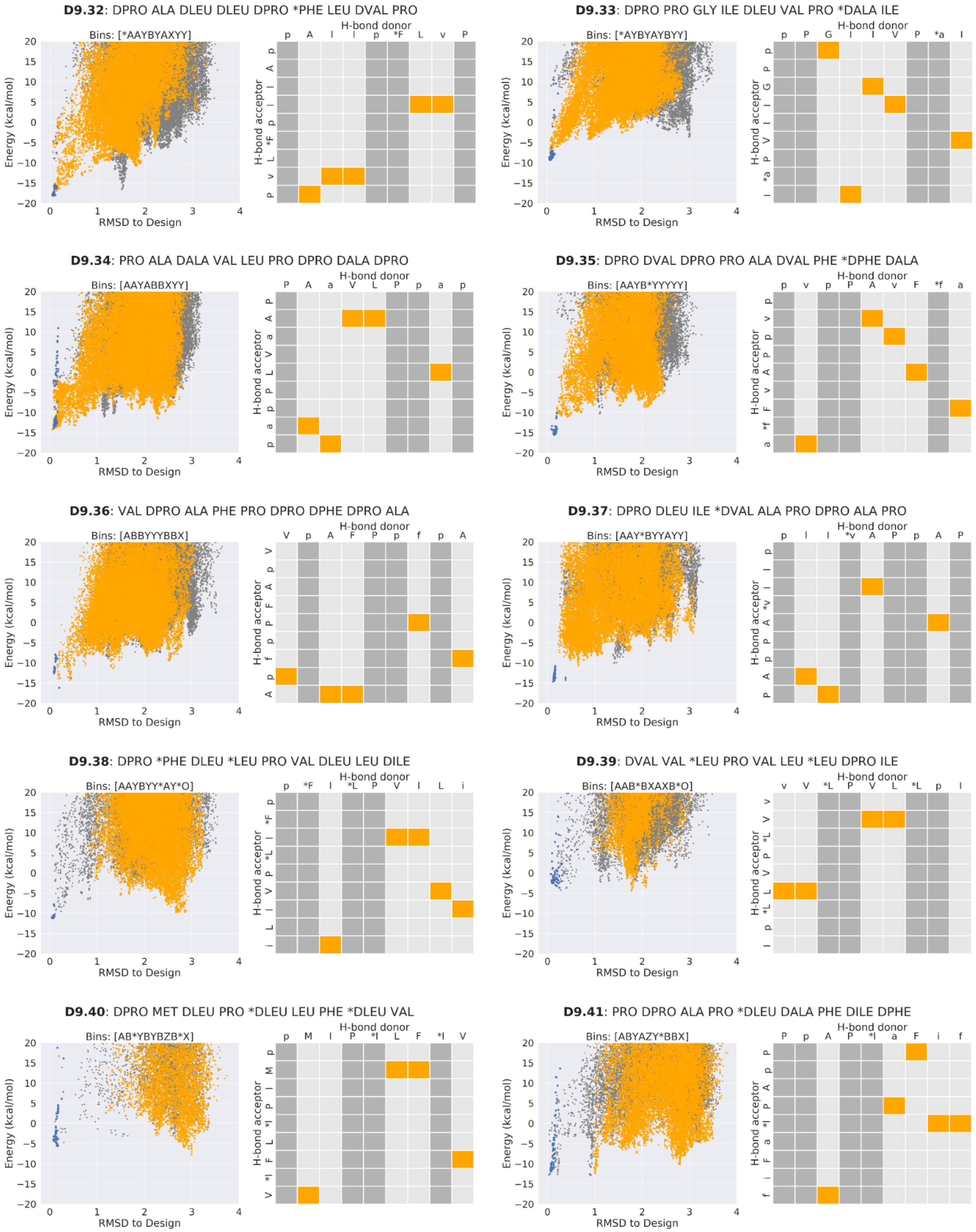

Figure 1.4 (cont.): Structure-energy landscapes and hydrogen-bonding patterns for designed and selected 9-amino acid macrocycles. *(full figure legend described earlier).* Related to Figure 3.

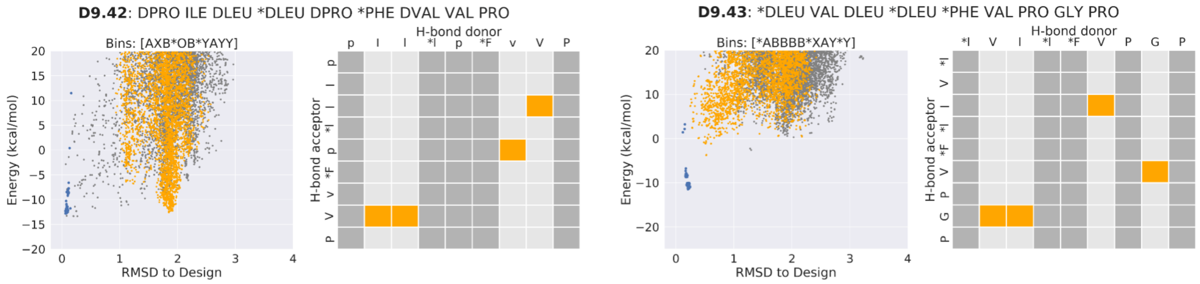

Figure D1.5: Structure-energy landscapes and hydrogen-bonding patterns for designed and selected 10-amino acid macrocycles: Left panels show the torsion bin cluster and predicted conformational landscape for each design. * denotes N-methylated amino acids. Orange points denote an independently predicted structure with no cis-peptide bonds. Grey points represent predicted structures with at least one cis-peptide bond. Blue dots represent the local minimization of the designed macrocycle structure. For hydrogen bonding graphs, the orange boxes highlight the designed intramolecular hydrogen bonds. Amino acids without a backbone hydrogen bond donor (proline, D-proline, N-methylated amino acids) are marked by darker grey columns. Related to Figure 3.

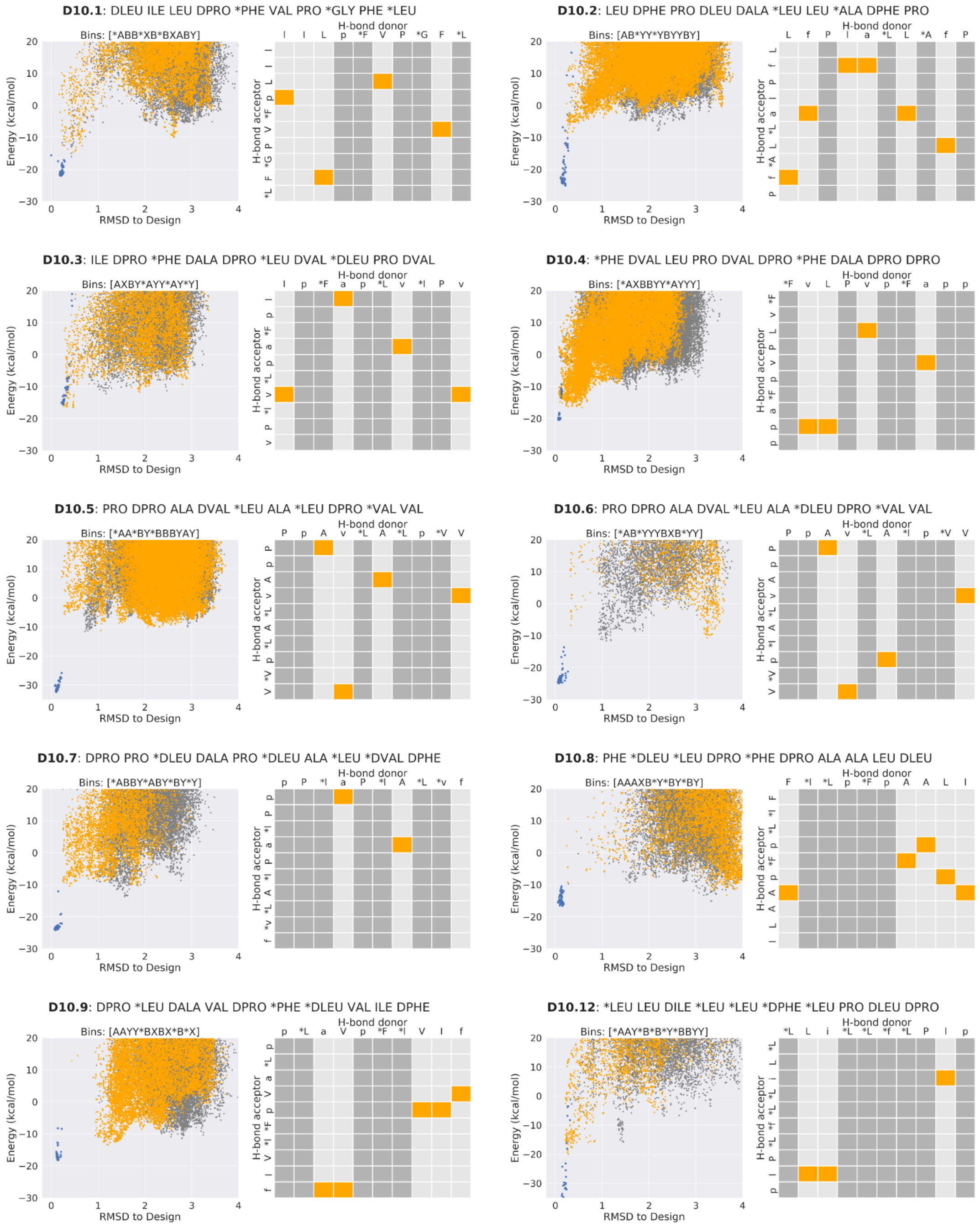

Figure D1.5 (cont.): Structure-energy landscapes and hydrogen-bonding patterns for designed and selected 10-amino acid macrocycles. *(full figure legend described earlier).* Related to Figure 3.

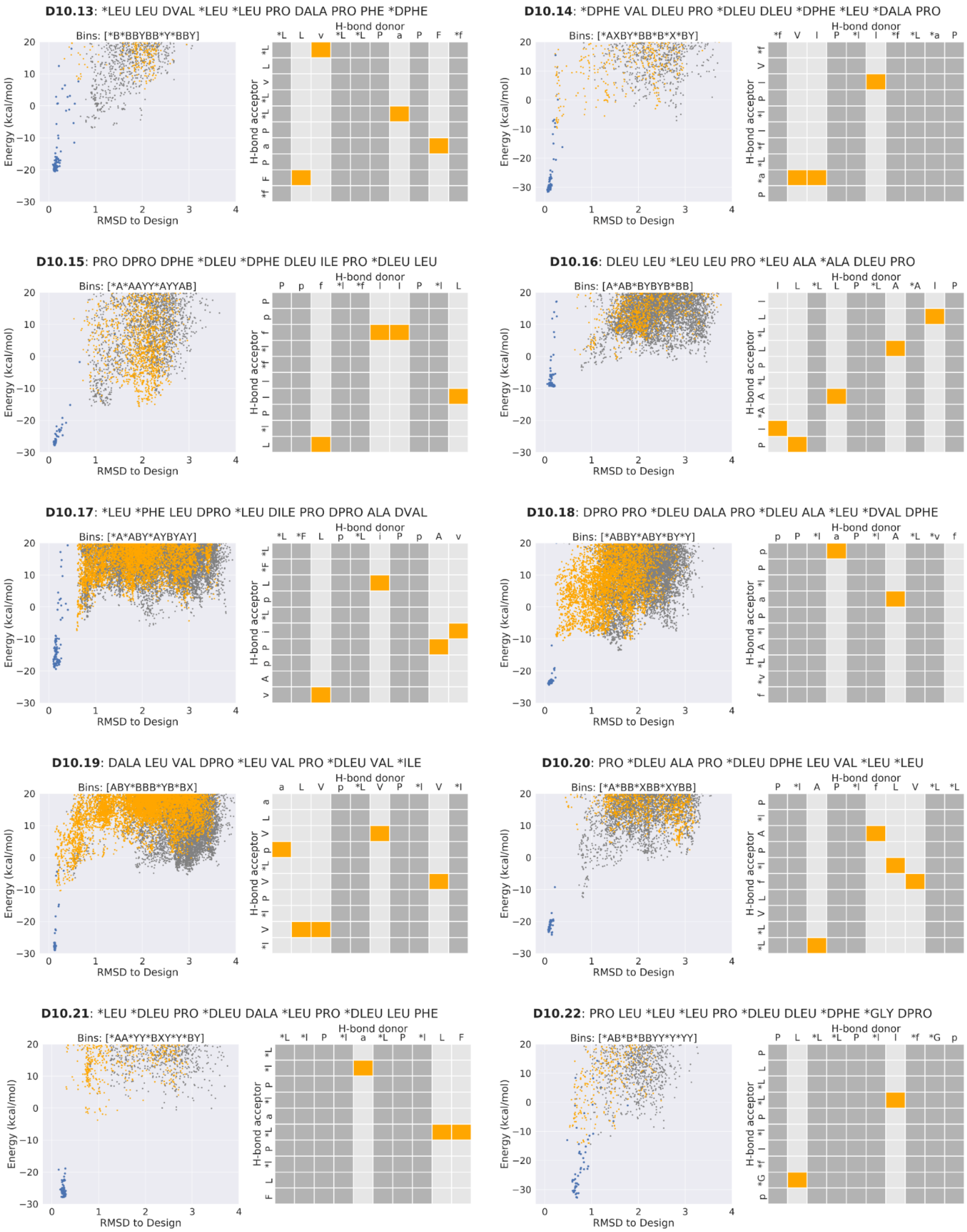

Figure D1.5 (cont.): Structure-energy landscapes and hydrogen-bonding patterns for designed and selected 10-amino acid macrocycles. *(full figure legend described earlier).* Related to Figure 3.

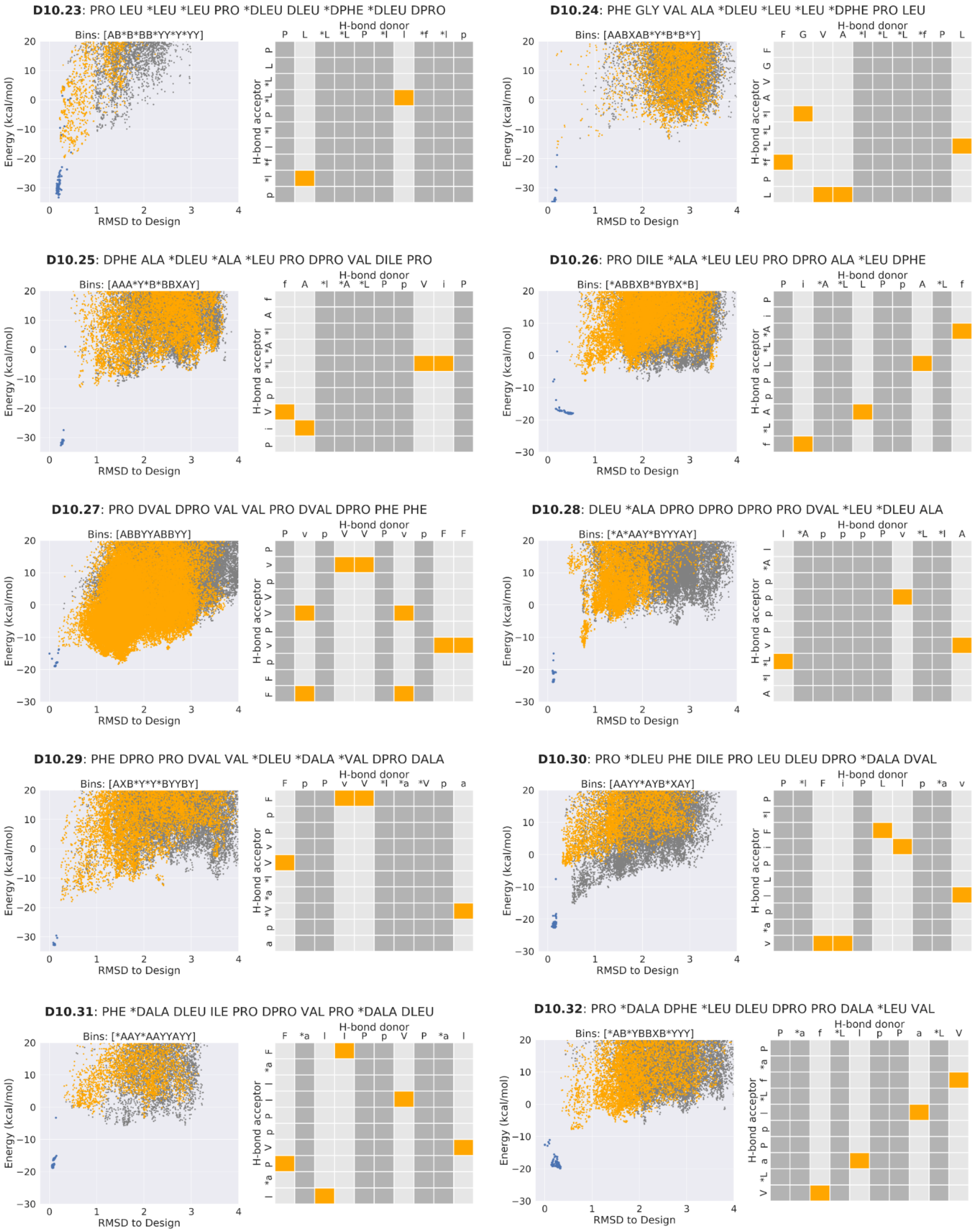

Figure D1.5 (cont.): Structure-energy landscapes and hydrogen-bonding patterns for designed and selected 10-amino acid macrocycles. *(full figure legend described earlier).* Related to Figure 3.

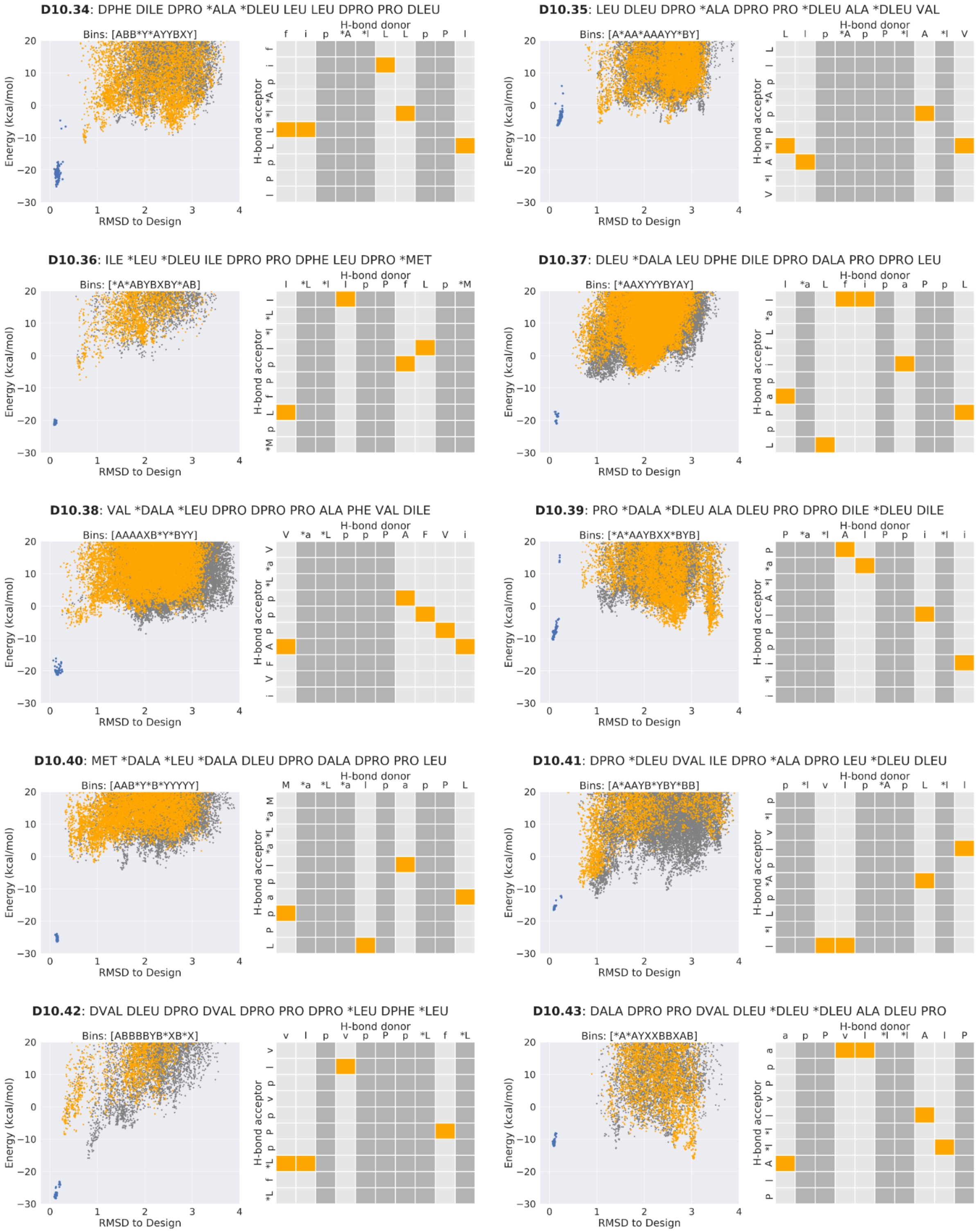

Figure D1.5 (cont.): Structure-energy landscapes and hydrogen-bonding patterns for designed and selected 10-amino acid macrocycles. *(full figure legend described earlier).* Related to Figure 3.

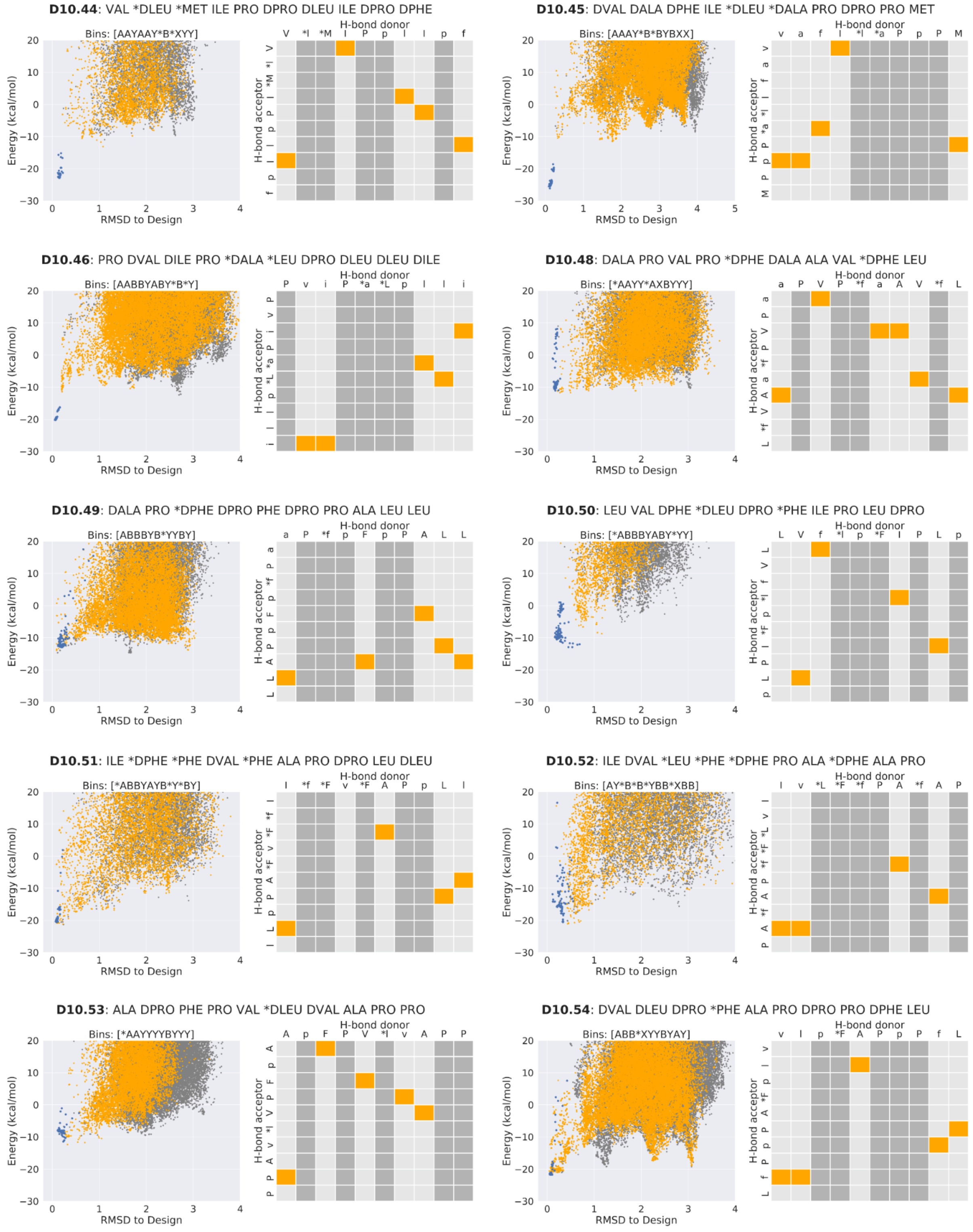

Figure D1.5 (cont.): Structure-energy landscapes and hydrogen-bonding patterns for designed and selected 10-amino acid macrocycles. *(full figure legend described earlier).* Related to Figure 3.

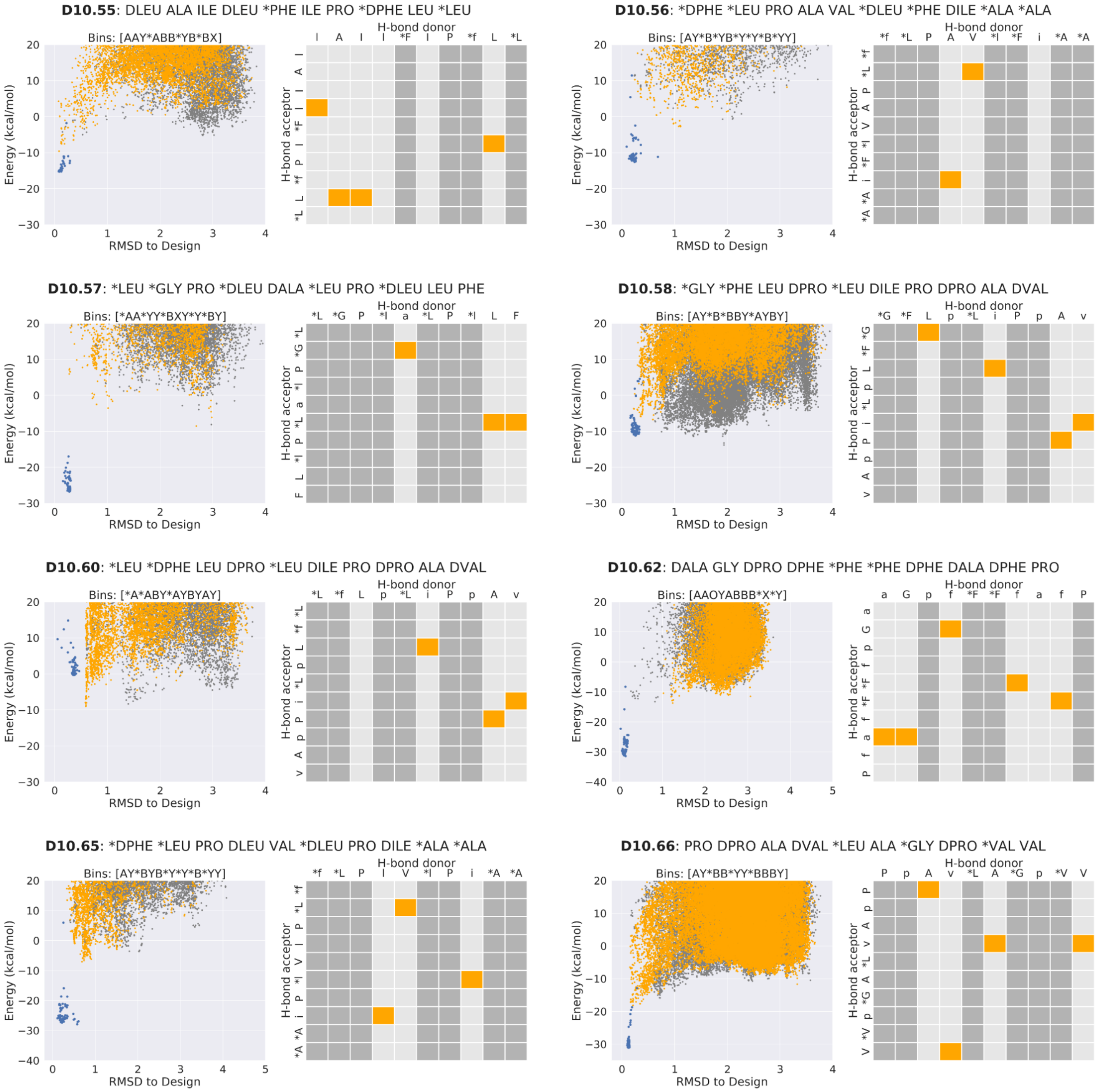

Figure 1.6: Structure-energy landscapes and hydrogen-bonding patterns for designed and selected 11-amino acid macrocycles: Left panels show the torsion bin cluster and predicted conformational landscape for each design. * denotes N-methylated amino acids. Orange points denote an independently predicted structure with no cis-peptide bonds. Grey points represent predicted structures with at least one cis-peptide bond. Blue dots represent the local minimization of the designed macrocycle structure. For hydrogen bonding graphs, the orange boxes highlight the designed intramolecular hydrogen bonds. Amino acids without a backbone hydrogen bond donor (proline, D-proline, N-methylated amino acids) are marked by darker grey columns. Related to Figure 3.

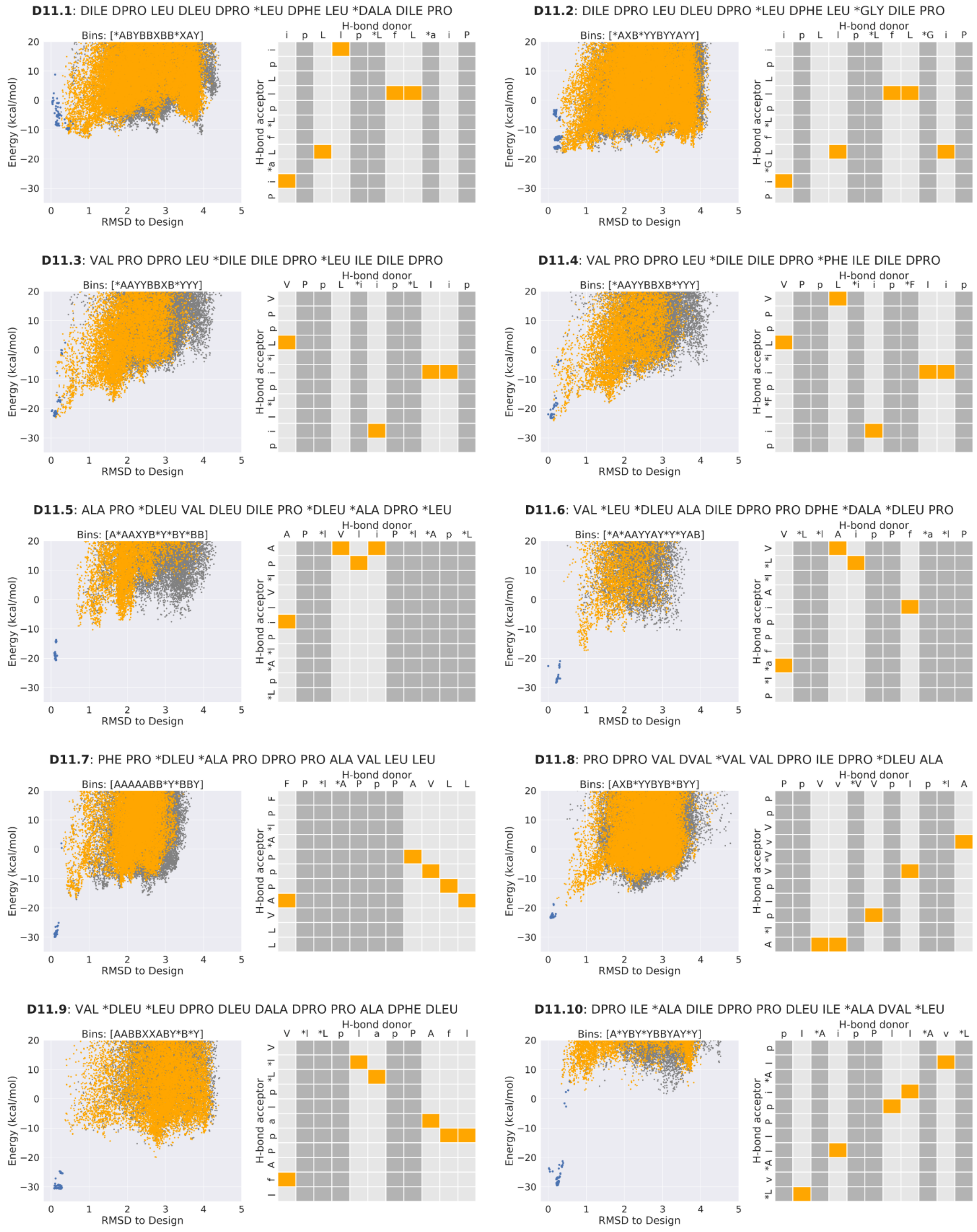

Figure D1.6 (cont.): Structure-energy landscapes and hydrogen-bonding patterns for designed and selected 11-amino acid macrocycles. *(full figure legend described earlier).* Related to Figure 3.

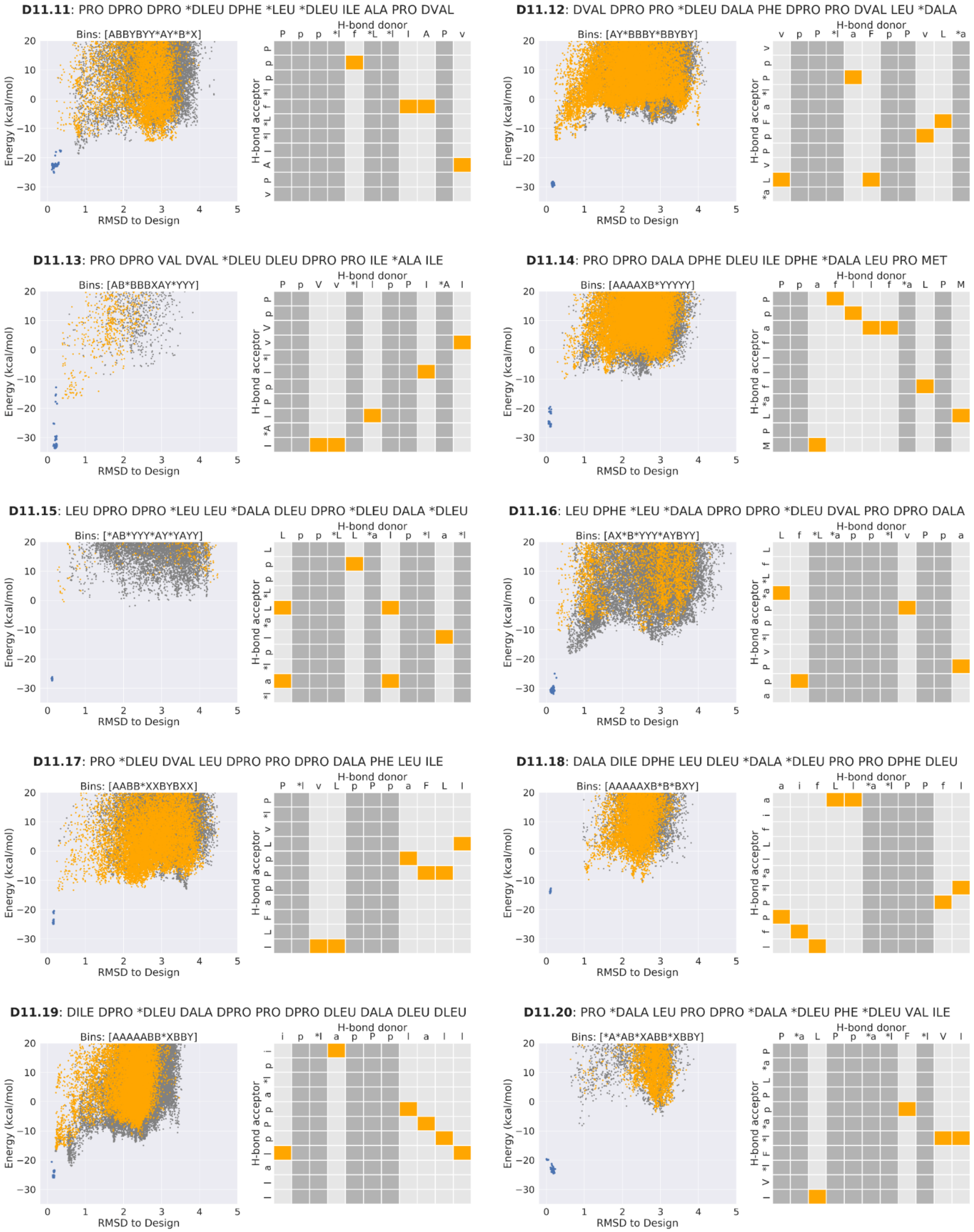

Figure 1.6 (cont.): Structure-energy landscapes and hydrogen-bonding patterns for designed and selected 11-amino acid macrocycles. *(full figure legend described earlier).* Related to Figure 3.

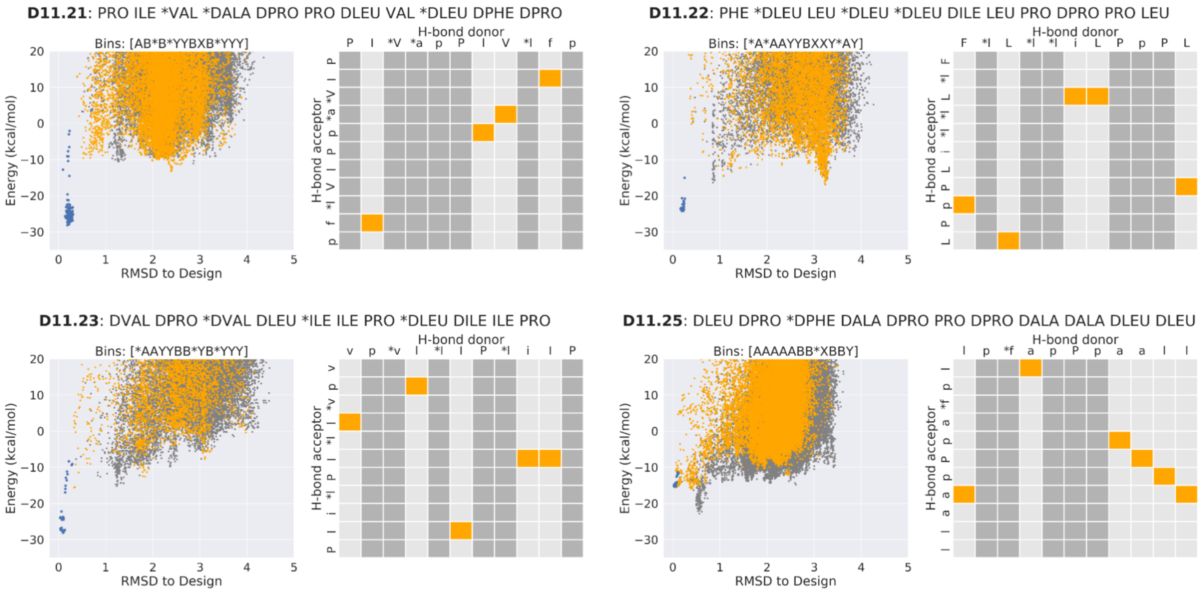

Figure S1.7: Structure-energy landscapes and hydrogen-bonding patterns for designed and selected 12-amino acid macrocycles: Left panels show the torsion bin cluster and predicted conformational landscape for each design. * denotes N-methylated amino acids. Orange points denote an independently predicted structure with no cis-peptide bonds. Grey points represent predicted structures with at least one cis-peptide bond. Blue dots represent the local minimization of the designed macrocycle structure. For hydrogen bonding graphs, the orange boxes highlight the designed intramolecular hydrogen bonds. Amino acids without a backbone hydrogen bond donor (proline, D-proline, N-methylated amino acids) are marked by darker grey columns. Related to Figure 3.

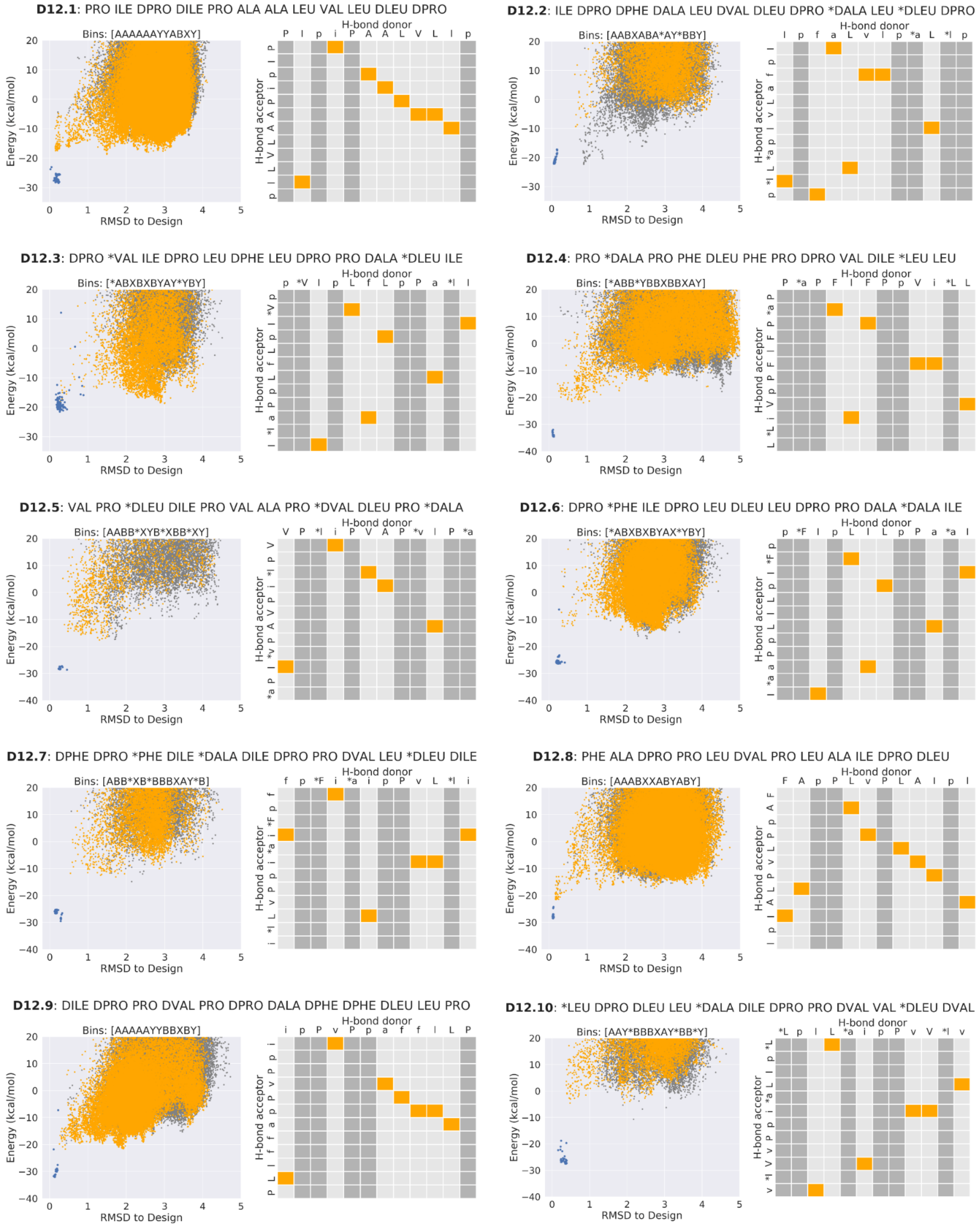

Supplement: Data S1. Structure prediction plots for all designs, related to Figures 1 and 3 [file mmc5.pdf]
