# Supplementary material for: Accurate de novo design of membrane-traversing macrocycles
Source: Cell. 2022 Sep 15;185(19):3520–3532.e26. doi: 10.1016/j.cell.2022.07.019 (PMC9490236; doi:10.1016/j.cell.2022.07.019)
Supplement: Data S4. Oral bioavailability data, related to Figure 5 [file mmc8.pdf]

Accurate *de novo* design of membrane-traversing macrocycles

Supplemental Data File 4: Oral Bioavailability Data. Related to Figure 5**.**

Table 5.1: Pharmacokinetic data for D8.3.p1: Mean plasma levels after PO, SQ, and IV dosing in Balb/C female mice. Related to Figure 5.

		D8.3.p1 Mean plasma levels (μM)			
	**(min)**	PO	SQ	IV	
	**5**			1.07 ± 0.07	
	**15**	0.19 ± 0.04	0.61 ± 0.13	0.53 ± 0.11	
	**30**	0.19 ± 0.03	0.78 ± 0.28	0.27 ± 0.09	
	**60**	0.12 ± 0.04	0.9 ± 0.05	0.06 ± 0.04	
	**120**	0 ± 0.01	0.78 ± 0.09		
	**240**		0.34 ± 0.17		
	**360**		0.13 ± 0.12		
	**1440**		0.01 ± 0.00		
Time of max concentration	**Tmax**	25 ± 0.01	50 ± 17.32		***(min)***
Max concentration	**Cmax**	0.21± 0.02	0.95 ± 0.12		***(μM)***
Initial concentration	**C0**			1.53 ± 0.07	***(μM)***
Volume of distribution @ steady state	**VSS**			0.72 ± 0.09	***(L/kg)***
Volume of distribution during elimination phase	**VZ**			0.78 ± 0.17	***(L/kg)***
Area-under-the-curve to last measureable concentration	**AUC0-t**	10.93 ± 4.89	223.75 ± 133.45	25.44 ± 3.73	***(min*μmol/L)***
Area-under-the-curve extrapolated to infinity	**AUC0-∞**	14.30 ± 3.71	246.92 ± 99.85	26.62 ± 3.96	***(min*μmol/L)***
Half-life	**T1/2**	29.15 ± 14.12	154.61 ± 83.41	12.08 ± 2.13	***(min)***
Apparent clearance	**Cl/F**	8.34E-03	5.02E-04		***(L/min)***
Plasma clearance	**CLP**			41.78 ± 6.42	***(mL/min/kg)***
Mean residence time	**MRT**			14.60 ± 1.29	***(min)***
Oral bioavailability	**F**	10.74 ± 2.79			***%***

Table 5.2: Plasma levels (μM) of D8.3.p1 after PO dosing (5 mg/kg) in Balb/C female mice. Related to Figure 5.

(min)	M1	M2	M3	AVG	
15	0.22	0.2	0.14	0.19	
30	0.17	0.23	0.19	0.19	
60	0.06	0.18	0.12	0.12	
120	0	0.01	0	0	
240	0	0	0	0	
360	0	0	0	0	
1440	0	0	0	0	
**Tmax**	15	30	30	25	*(min)*
**Cmax**	0.22	0.23	0.19	0.21	*(μM)*
**AUC0-t**	8.03	16.58	8.18	10.93	*(min*μmol/L)*
**AUC0-∞**	10.04	16.85	16.01	14.3	*(min*μmol/L)*
**T1/2**	23.33	18.86	45.25	29.15	*(min)*
**Cl/F**	1.12E-02	6.71E-03	7.06E-03	8.34E-03	*(L/min)*

Table 5.3: Plasma levels (μM) of D8.3.p1 after SQ dosing (5 mg/kg) in Balb/C female mice. Related to Figure 5.

(min)	M1	M2	M3	AVG	
15	0.51	0.7	ND	0.61	
30	0.54	1.08	0.71	0.78	
60	0.84	0.92	0.93	0.9	
120	0.72	0.73	0.88	0.78	
240	0.23	0.26	0.53	0.34	
360	0.15	0	0.23	0.13	
1440	0	0	0.02	0.01	
**Tmax**	60	30	60	50	*(min)*
**Cmax**	0.84	1.08	0.93	0.95	*(μM)*
**AUC0-t**	159	157.5	354.75	223.75	*(min*μmol/L)*
**AUC0-∞**	183.06	195.71	361.99	246.92	*(min*μmol/L)*
**T1/2**	111.2	101.86	250.78	154.61	*(min)*
**Cl/F**	6.17E-04	5.77E-04	3.12E-04	5.02E-04	*(L/min)*

Table 5.4: Plasma levels (μM) of D8.3.p1 after IV dosing (1 mg/kg) in Balb/C female mice. Related to Figure 5.

(min)	M1	M2	M3	AVG	
5	1.11	0.99	1.11	1.07	
15	0.53	0.42	0.63	0.53	
30	0.26	0.19	0.37	0.27	
60	0.1	0.04	0.03	0.06	
120	0	0	0	0	
240	0	0	0	0	
360	0	0	0	0	
**T1/2**	14.5	10.5	11.24	12.08	***(min)***
**C0**	1.61	1.52	1.47	1.53	***(μM)***
**VSS**	0.79	0.75	0.62	0.72	***(L/kg)***
**VZ**	0.89	0.87	0.59	0.78	***(L/kg)***
**AUC0-t**	26.32	21.35	28.66	25.44	***(min*μmol/L)***
**AUC0-∞**	28.69	22.06	29.12	26.62	***(min*μmol/L)***
**CLP**	37.62	49.17	38.55	41.78	***(mL/min/kg)***
**MRT**	15.45	13.12	15.23	14.6	***(min)***

Table 5.5: Pharmacokinetic data for D10.1: Mean plasma levels after PO, SQ, and IV dosing in Balb/C female mice. Related to Figure 5.

		D10.1 Mean plasma levels (µM)			
	**(min)**	PO	SQ	IV	
	**5**			21.00 ± 5.99	
	**15**	2.58 ± 0.59	2.53 ± 1.18	7.58 ± 1.95	
	**30**	2.92 ± 0.63	3.55 ± 1.67	4.92 ± 2.11	
	**60**	1.78 ± 0.31	3.93 ± 1.78	2.19 ± 0.29	
	**120**	0.72 ± 0.15	ND	1.03 ± 0.06	
	**240**	0.16 ± 0.03	ND	0.44 ± 0.03	
	**360**	0.07 ± 0.06	2.29 ± 0.15	0.31 ± 0.09	
	**1440**		0.01 ± 0.02		
Time of max concentration	**Tmax**	30 ± 0.00	150 ± 182.48		***(min)***
Max concentration	**Cmax**	2.92 ± 0.63	4.14 ± 1.51		***(µM)***
Initial concentration	**C0**			37.77 ± 21.43	***(µM)***
Volume of distribution @ steady state	**VSS**			0.25 ± 0.08	***(L/kg)***
Volume of distribution during elimination phase	**VZ**			0.32 ± 0.07	***(L/kg)***
Area-under-the-curve to last measureable concentration	**AUC0-t**	270.40 ± 55.52	1879.5 ± 1501.5	720.90 ± 121.67	***(min*µmol/L)***
Area-under-the-curve extrapolated to infinity	**AUC0-∞**	279.94 ± 55.31	2867.2 ± 1016.5	751.94 ± 110.39	***(min*µmol/L)***
Half-life	**T1/2**	60.29 ± 9.35	225.38 ± 48.70	67.38 ± 6.78	***(min)***
Apparent clearance	**Cl/F**	7.85E-04	7.96E-05		***(L/min)***
Plasma clearance	**CLP**			3.26 ± 0.37	***(mL/min/kg)***
Mean residence time	**MRT**			59.54 ± 10.01	***(min)***
Oral bioavailability	**F**	7.48 ± 1.79			***%***

Table 5.6: Plasma levels (μM) of D10.1 after PO dosing (12.2 mg/kg) in Balb/C female mice. Related to Figure 5.

(min)	M1	M2	M3	AVG	
15	2.22	3.26	2.26	2.58	
30	2.49	3.64	2.64	2.92	
60	1.57	2.14	1.64	1.78	
120	0.54	0.8	0.81	0.72	
240	0.13	0.18	0.17	0.16	
360	0	0.1	0.1	0.07	
1440	0	0	0	0	
**Tmax**	30	30	30	30	*(min)*
**Cmax**	2.49	3.64	2.64	2.92	*(μM)*
**AUC0-t**	216.96	327.78	266.45	270.4	*(min*μmol/L)*
**AUC0-∞**	226.62	337.03	276.18	279.94	*(min*μmol/L)*
**T1/2**	49.88	63.05	67.95	60.29	*(min)*
**Cl/F**	9.44E-04	6.35E-04	7.75E-04	7.85E-04	*(L/min)*

Table 5.7: Plasma levels (μM) of D10.1 after SQ dosing (12.2 mg/kg) in Balb/C female mice. Related to Figure 5.

(min)	M1	M2	M3	AVG	
15	1.39	2.44	3.75	2.53	
30	1.81	3.69	5.15	3.55	
60	1.87	4.88	5.03	3.93	
120	ND	ND	ND	ND	
240	ND	ND	ND	ND	
360	2.4	ND	2.19	2.29	
1440	0	0.03	0	0.01	
**Tmax**	360	60	30	150	*(min)*
**Cmax**	2.4	4.88	5.15	4.14	*(μM)*
**AUC0-t**	730.93	3578.6	1329.09	1879.54	*(min*μmol/L)*
**AUC0-∞**	NA	3585.94	2148.41	2867.17	*(min*μmol/L)*
**T1/2**	NA	190.94	259.81	225.38	*(min)*
**Cl/F**	NA	5.97E-05	9.96E-05	7.96E-05	*(L/min)*

Table 5.8: Plasma levels (μM) of D10.1 after IV dosing (2.45 mg/kg) in Balb/C female mice. Related to Figure 5.

(min)	M1	M2	M3	AVG	
5	15.45	20.19	27.35	21	
15	9.12	8.25	5.39	7.58	
30	4.05	3.37	7.33	4.92	
60	1.91	2.17	2.49	2.19	
120	1.1	1	1	1.03	
240	0.47	0.41	0.45	0.44	
360	0.37	0.36	0.21	0.31	
**T1/2**	72.41	70.07	59.67	67.38	***(min)***
**C0**	20.11	31.58	61.61	37.77	***(μM)***
**VSS**	0.32	0.28	0.16	0.25	***(L/kg)***
**VZ**	0.36	0.35	0.24	0.32	***(L/kg)***
**AUC0-t**	634.82	667.79	860.1	720.9	***(min*μmol/L)***
**AUC0-∞**	673.47	704.18	878.17	751.94	***(min*μmol/L)***
**CLP**	3.46	3.48	2.83	3.26	***(mL/min/kg)***
**MRT**	68.66	61.13	48.84	59.54	***(min)***

Table 5.9: Plasma concentration (ng/mL) of D11.3 after IV (1.00 mg/kg) dose administration in male Swiss Albino mice. Related to Figure 5.

Time (h)	M1 -	M2-	M3-	Mean	Std Dev	% CV
0.08	3319.8	4091.53	3825.09	**3745.47**	391.98	10.47
0.25	3305.02	3779.15	2973.54	**3352.57**	404.9	12.08
0.5	1656.03	2566.56	1814.39	**2012.33**	486.47	24.17
1	931.71	1218.22	1283.47	**1144.47**	187.12	16.35
3	887.86	328.77	318.62	**511.75**	325.76	63.66
5	240.02	271.55	289.17	**266.91**	24.9	9.33
8	399.46	124.26	182.53	**235.42**	145.02	61.6
24	17.65	30.97	34.16	**27.59**	8.76	31.74
Dose (mg/kg)	1	1	1	**1**	0	0
C0 (ng/mL)	-	-	-	**3957.56**	-	-
T1/2 (h)	-	-	-	**5.58**	-	-
AUC0-last (ng·h/mL)	-	-	-	**6965.11**	-	-
AUC0-inf (ng·h/mL)	-	-	-	**7187.15**	-	-
AUCExtra(%)	-	-	-	**3.09**	-	-
Cl (ml/min/kg)	-	-	-	**2.32**	-	-
Vd (L/kg)	-	-	-	**1.12**	-	-
MRT0-last (h)	-	-	-	**4.64**	-	-
Rsq	-	-	-	**0.9904**	-	-

Table 5.10: Plasma concentration (ng/mL) of D11.3 after PO (10.00 mg/kg) dose administration in male Swiss Albino mice. Related to Figure 5.

Time (h)	M1 -	M2-	M3-	Mean	Std Dev	% CV
0.08	47.49	76.49	84.94	**69.64**	19.64	28.21
0.25	454	804.64	992.29	**750.31**	273.23	36.42
0.5	1880.71	2858.02	2832.99	**2523.91**	557.17	22.08
1	2950.38	3417.67	2525.44	**2964.5**	446.28	15.05
3	3249.94	3660.57	3578.88	**3496.46**	217.37	6.22
5	3313.72	2427.94	1866.9	**2536.19**	729.46	28.76
8	1232.5	1764.28	1439.78	**1478.85**	268.03	18.12
24	177.35	48.47	59.29	**95.04**	71.49	75.22
Dose (mg/kg)	10	10	10	**10**	0	0
Cmax (ng/mL)	-	-	-	**3496.46**	-	-
Tmax (h)	-	-	-	**3**	-	-
AUC0-last (ng·h/mL)	-	-	-	**28242.38**	-	-
AUC0-inf (ng·h/mL)	-	-	-	**28793.58**	-	-
AUCExtra(%)	-	-	-	**1.91**	-	-
MRT0-last (h)	-	-	-	**6.32**	-	-
F (%)	-	-	-	**40.06**	-	-
Rsq	-	-	-	**1**	-	-

Table 5.11: Plasma concentration (ng/mL) of D11.3 after SC (5.00 mg/kg) dose administration in male Swiss Albino mice. Related to Figure 5.

Time (h)	M1 -	M2-	M3-	Mean	Std Dev	% CV
**PD**	0	0	0	**0**	0	#DIV/0!
0.08	12.41	9.86	8.17	**10.15**	2.13	21.04
0.25	60.87	22.1	22.16	**35.04**	22.37	63.83
0.5	22.46	23.29	70.26	**38.67**	27.36	70.75
1	52.72	45.05	34.59	**44.12**	9.1	20.63
3	141.22	103.85	139.25	**128.11**	21.03	16.42
5	117.42	136.91	188.23	**147.52**	36.58	24.8
8	153.34	93.4	96.35	**114.36**	33.79	29.54
24	57.62	60.78	33.12	**50.51**	15.14	29.98
Dose (mg/kg)	5	5	5	**5**	0	0
Cmax (ng/mL)	-	-	-	**147.52**	-	-
Tmax (h)	-	-	-	**5**	-	-
AUC0-last (ng·h/mL)	-	-	-	**2122.81**	-	-
AUC0-inf (ng·h/mL)	-	-	-	**3047.15**	-	-
AUCExtra(%)	-	-	-	**30.33**	-	-
MRT0-last (h)	-	-	-	**10.71**	-	-
F (%)	-	-	-	**8.48**	-	-
Rsq	-	-	-	**0.9933**	-	-

Table 5.12: Plasma levels for D11.2 after IV, SQ, and PO dosing (1 mg/kg) in male SD rats. Related to Figure 5.

Route	Subject	T1/2z	Tmax	Cmax	C0	AUClast	AUC∞	Vz	CL	MRT∞	Vss	%F
		hr	hr	ng/mL	ng/mL	h*ng/mL	h*ng/mL	L/kg	L/hr/kg	hr	L/kg	
IV	R1	2.08	0.0833	499	626	642	674	4.46	1.48	2.18	3.23	NA
IV	R2	2.05	0.0833	519	669	702	738	4.00	1.35	2.34	3.17	NA
IV	R3	2.74	0.0833	796	1234	858	926	4.27	1.08	2.51	2.72	NA
**IV**	**Mean**	**2.29**	**0.0833**	**605**	**843**	**734**	**780**	**4.24**	**1.31**	**2.35**	**3.04**	**NA**
IV	SD	0.389	0.0000	166	339	111	131	0.231	0.206	0.169	0.283	NA
PO	R4	2.42	0.500	31.3	NA	66.8	81.3	NA	NA	3.38	NA	12.1
PO	R5	2.06	0.250	35.2	NA	89.3	95.9	NA	NA	3.02	NA	13.0
PO	R6	2.53	0.500	28.2	NA	59.1	75.8	NA	NA	3.86	NA	8.18
**PO**	**Mean**	**2.33**	**0.416**	**31.6**	**NA**	**71.7**	**84.3**	**NA**	**NA**	**3.42**	**NA**	**11.1**
PO	SD	0.246	0.144	3.51	NA	15.7	10.4	NA	NA	0.422	NA	2.55
SQ	R7	4.52	4.00	131	NA	1040	1080	NA	NA	6.81	NA	160
SQ	R8	5.00	4.00	99.4	NA	741	777	NA	NA	7.64	NA	105
SQ	R9	3.71	4.00	95.1	NA	841	853	NA	NA	6.14	NA	92.1
**SQ**	**Mean**	**4.41**	**4.00**	**109**	**NA**	**874**	**903**	**NA**	**NA**	**6.86**	**NA**	**119**
SQ	SD	0.654	0.00	19.6	NA	152	158	NA	NA	0.753	NA	36.2
